# Bone loss with aging is independent of gut microbiome in mice

**DOI:** 10.1038/s41413-024-00366-0

**Published:** 2024-11-11

**Authors:** Xiaomeng You, Jing Yan, Jeremy Herzog, Sabah Nobakhti, Ross Campbell, Allison Hoke, Rasha Hammamieh, R. Balfour Sartor, Sandra Shefelbine, Melissa A. Kacena, Nabarun Chakraborty, Julia F. Charles

**Affiliations:** 1 Department of Orthopaedic Surgery, Brigham and Women’s Hospital, Harvard Medical School, Boston, MA 02115 USA; 2 National Gnotobiotic Rodent Resource Center, Center for Gastrointestinal Biology and Disease, University of North Carolina at Chapel Hill, Chapel Hill, NC 27599 USA; 3 Department of Mechanical and Industrial Engineering, Northeastern University, Boston, MA 02115 USA; 4 The Geneva Foundation, Walter Reed Army Institute of Research, Silver Spring, MD 20910 USA; 5 Medical Readiness Systems Biology, Walter Reed Army Institute of Research, Silver Spring, MD 20910 USA; 6 ORISE, Walter Reed Army Institute of Research, Silver Spring, MD 20910 USA; 7 Department of Orthopaedic Surgery, Indiana University School of Medicine, Indianapolis, IN 46202 USA; 8 Richard L. Roudebush VA Medical Center, Indianapolis, IN 46202 USA; 9 Division of Rheumatology, Brigham and Women’s Hospital, Harvard Medical School, Boston, MA 02115 USA

**Keywords:** Bone, Metabolomics

## Abstract

Emerging evidence suggests a significant role of gut microbiome in bone health. Aging is well recognized as a crucial factor influencing the gut microbiome. In this study, we investigated whether age-dependent microbial change contributes to age-related bone loss in CB6F1 mice. The bone phenotype of 24-month-old germ-free (GF) mice was indistinguishable compared to their littermates colonized by fecal transplant at 1-month-old. Moreover, bone loss from 3 to 24-month-old was comparable between GF and specific pathogen-free (SPF) mice. Thus, GF mice were not protected from age-related bone loss. 16S rRNA gene sequencing of fecal samples from 3-month and 24-month-old SPF males indicated an age-dependent microbial shift with an alteration in energy and nutrient metabolism potential. An integrative analysis of 16S predicted metagenome function and LC-MS fecal metabolome revealed an enrichment of protein and amino acid biosynthesis pathways in aged mice. Microbial S-adenosyl methionine metabolism was increased in the aged mice, which has previously been associated with the host aging process. Collectively, aging caused microbial taxonomic and functional alteration in mice. To demonstrate the functional importance of young and old microbiome to bone, we colonized GF mice with fecal microbiome from 3-month or 24-month-old SPF donor mice for 1 and 8 months. The effect of microbial colonization on bone phenotypes was independent of the microbiome donors’ age. In conclusion, our study indicates age-related bone loss occurs independent of gut microbiome.

## Introduction

Osteoporosis is characterized by a low bone mass and micro-architectural deterioration of bone tissue, leading to compromised bone strength and increased risk for fractures.^1^ It is a common chronic metabolic bone disease, affecting 200 million individuals worldwide.^1^ Aging is a well-recognized risk factor for osteoporosis and osteoporotic fractures. Approximately 10 million Americans aged over 50 have osteoporosis and an estimated 50% women and 20% men will suffer an osteoporotic fracture in their lifetime.^2^ With the rapid growth of aged population, the prevalence of osteoporosis and osteoporotic fractures is continuingly increasing, placing a heavy burden on economic cost and health care systems.^2^

Current treatments for osteoporosis, including both antiresorptive agents (e.g bisphosphonates, denosumab) to inhibit bone resorption or osteoanabolic agents (e.g Teriparatide) to stimulate bone formation, are effective at reducing fracture risk.^1^ However, concerns over side effects, particularly with long term use, and as well as other factors (e.g medication cost, access to medical care, lack of education about treatment) reduce adherence and contribute to poor persistence with osteoporosis therapy, leading to a significant number of patients remaining at risk for osteoporotic fractures.^3–6^ These factors have increased interest in non-pharmacologic interventions to improve bone health, including whether the gut microbiome could be leveraged for therapeutic benefit.

Recent studies suggest that the gut microbiome, the collection of microorganisms that reside in the host gastrointestinal (GI) tract, participates in the modulation of bone health. Colonization of gut microbiome in germ-free (GF) mice has been shown to promote bone growth by inducing IGF-1.^7^ On the other hand, depletion of gut microbes caused by antibiotics leads to impaired tissue mechanical properties and reduced bone strength.^8,9^ Moreover, in various preclinical models, it has been shown that bone loss resulting from sex steroid deficiency,^10^ glucocorticoid treatments^11^ and continuous PTH treatments^12^ is microbiome-dependent. Supplementation with probiotics (e.g *Lactobacillus*) prevented impaired bone growth due to chronic undernutrition,^13^ and exhibit beneficial effects on the bone loss induced by antibiotics,^14^ as well as in several preclinical osteoporosis models.^10,11,15^ These findings clearly suggest an important role of the gut microbiome in regulating bone mass, bone quality, and bone strength. Thus, the gut microbiome is emerging as a promising target for the prevention and treatment of age-related bone loss and osteoporosis.

It is well documented that the human gut microbiome diversifies with age.^16^ In early life, the gut microbiome is characterized by a relatively low diversity which is mainly dominated by Proteobacteria and Actinobacteria.^17^ The composition of the gut microbiome originally diversifies after weaning, develops into an adult-like microbial community, and reaches a relatively stable status in adulthood.^16^ With advancing age, the gut microbiome further undergoes a transition away from its composition in young adulthood.^18–22^ Our previous meta-analysis across 6 publicly available sequencing datasets revealed an age-dependent increase in gut microbiome α-diversity and as well as alterations in predicted metagenomic functions related to carbohydrate metabolism in C57BL/6 mice.^23^

However, it remains unclear whether these documented age-related microbial alterations directly affect bone loss with aging. In this study, we investigated whether bone loss with aging is dependent on gut microbiome in CB6F1 mice. Specifically, we examined whether the presence of gut microbiome is required for age-associated bone loss by comparing the bone phenotypes of GF mice with their colonized littermates at 24-month of age. We further compared bone loss with aging from young (3-month-old) to old (24-month-old) in both GF mice and conventionally raised specific pathogen free (SPF) mice. Fecal samples from young and old SPF mice were characterized by 16S rRNA gene sequencing and LC-MS to identify age-related alterations in microbial compositions and functional consequences. Finally, bone phenotypes were compared between the gnotobiotic CB6F1 mice colonized with fecal microbiome from young and old donors.

## Results

### GF mice are not protected from trabecular bone loss with aging in either sex

**Fig. 1:**
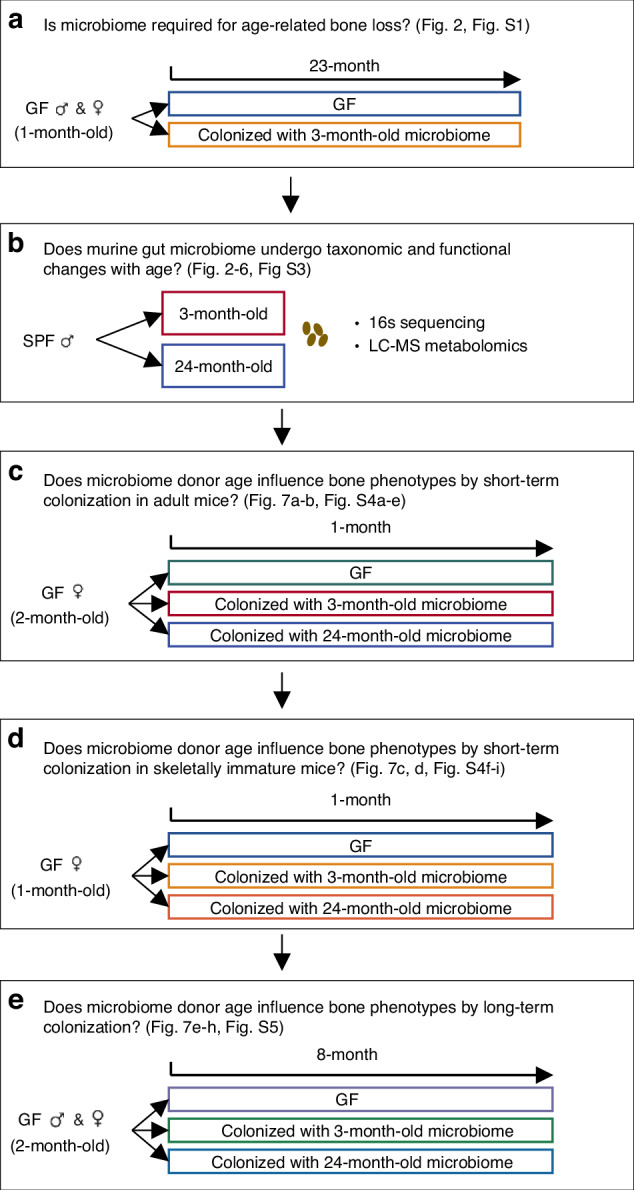
The workflow of study design. **a** 1-month-old CB6F1 germ-free (GF) mice were randomly assigned to 2 experimental groups: remain GF or colonization with microbiome from 3-month-old donors for 23 months. **b** Fecal samples were collected from 3-month-old and 24-month-old conventionally raised specific pathogen free (SPF) mice for gut microbiota characterization by 16S rRNA gene sequencing and LC-MS. 2-month-old CB6F1 GF mice were randomly assigned to remain GF or be colonized with fecal microbiome from 3-month-old or 24-month-old donors for (**c**) 1 month or (**e**) 8 months. **d** 1-month-old CB6F1 GF mice were randomized to remain GF or be colonized with microbiome from 3-month-old or 24-month-old donors for 1 month

**Fig. 2:**
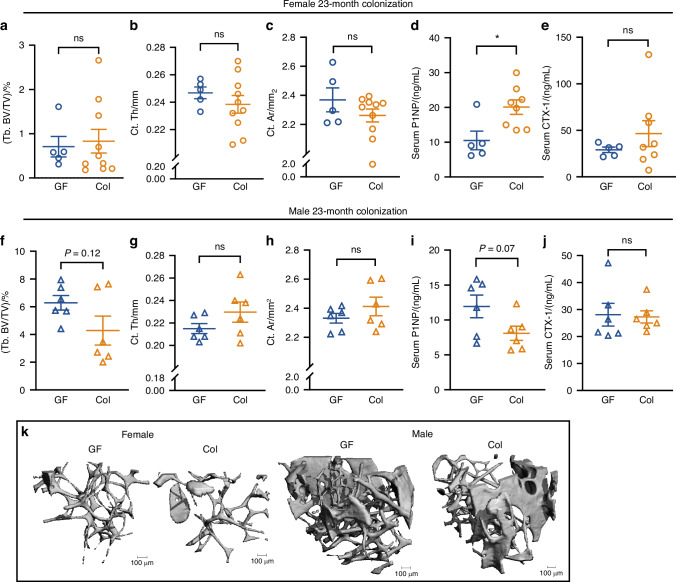
The presence of gut microbiome is not required for age-related trabecular bone loss. Trabecular bone volume fraction (Tb. BV/TV), cortical thickness (Ct. Th), cortical area (Ct. Ar), serum P1NP and CTX-1 of GF and colonized mice in both (**a–e**) female and (**f–j**) male mice are shown. **k** Representative 3D images of femur trabecular bone are shown. Data are represented as mean ± SEM. Unpaired *t* test or Mann-Whitney U Test was performed. ns, not statistically significant. For female, GF, *n* = 5; Col, *n* = 10. For male GF, *n* = 6; Col, *n* = 6. 2 data in (**d**) and (**e**) were missing due to insufficient sample collection

To understand whether the gut microbiome is required for age-related bone loss, we randomized 1-month-old GF CB6F1 littermates to remain GF or be colonized and examined the bone phenotype at 24-months of age in both sexes (Fig. 1a). No significant differences in trabecular bone volume fraction (Tb. BV/TV), cortical thickness (Ct. Th), or cortical area (Ct. Ar) were observed between the GF and colonized mice in either sex (Fig. 2a–c, f–h). Trabecular parameters were similar in colonized females (Fig. S1a–c). However, we cannot rule out an impact of microbiome on microarchitecture in males, as trabecular number (Tb. N) and trabecular spacing (Tb. Sp) differed significantly between colonized and GF male mice (Fig. S1f, h). Neither periosteal perimeter (Ps. Pm) or tissue mineral density (Ct. TMD) was changed by colonization (Fig. S1d, e, i, j), Therefore, GF mice demonstrated similar trabecular and cortical bone mass level compared to their colonized littermates at 24-month-old in both females and males. The presence of the gut microbiome was not required for bone loss with aging.

Bone turnover was assessed by measurement of serum bone formation and resorption markers, P1NP and CTX-1. P1NP was significantly increased in colonized female mice compared to GF female mice (*P* < 0.01, Fig. 2d), though colonization did not change serum P1NP in males (Fig. 2i) nor CTX-1 in either sex (Fig. 2e, j).

Additionally, the bone phenotypes of GF mice and conventionally raised SPF mice were assessed at 3 and 24 months of age in both sexes (Fig. S2a). In both GF and SPF female mice, Tb. BV/TV decreased from ~10% (GF: 10.27% ± 0.52%, SPF: 10.44% ± 0.73%) to less than 1% (GF: 0.56% ± 0.14%, SPF: 0.84% ± 0.16%) (Fig. S2b), with corresponding changes in other trabecular bone parameters (Fig. S2c–e). In males, Tb. BV/TV decreased from ~20% at 3-month (GF: 23.93% ± 1.01%, SPF: 19.60% ± 0.82%) to ~6% at 24-month (GF: 6.28% ± 0.52%, SPF: 6.49% ± 0.64%) of age (Fig. S2l), with corresponding changes in other trabecular bone parameters (Fig. S2m–o). Cortical area (Ct. Ar), periosteal perimeter (Ps. Pm) and tissue mineral density (Ct. TMD) showed an increase with aging in both female and male mice under both SPF and GF conditions (Fig. S2g–i, q–s).

Although cortical thickness is typically thought to decrease with age, published studies report increases, decreases, or no changes in femur diaphyseal cortical thickness (Ct. Th) with aging in mice, with variations observed across sexes and genetic backgrounds.^24–28^ In this study we found Ct. Th increased with aging in female GF and SPF CB6F1 mice (Fig. S2f), decreased with aging in male SPF mice but was unchanged with aging in GF males (Fig. S2p).

Quantitative backscatter scanning electron microscopy (qbSEM) was then applied to assess tissue mineralization properties of a subset of bones from the female cohort. Whereas microCT assesses structural properties (how much bone), qbSEM assesses the amount of mineral in the bone. The gray-level mode of the histogram of backscatter intensities of the tibial cross-section, which indicates amount of mineralization, was increased with age in both GF and SPF female mice (Fig. S2j). The FWHM did not differ by age (data not shown). Cortical porosity measured by SEM, which includes both lacunar and vascular space, decreased with age in both female groups (Fig. S2k). Taken together, GF mice demonstrated comparable bone loss as observed in SPF mice, thus GF mice are not protected from trabecular bone loss with aging in either sex.

### Age-dependent divergence in microbial ecosystem between the young and old male mice

Our previous meta-analysis across 6 publicly available datasets revealed an age-dependent shift in microbial diversities in C57BL/6 mice.^23^ To confirm that CB6F1 mice also undergo changes in microbial community in response to aging, 16S rRNA sequencing was performed to characterize gut microbiome from young and old SPF CB6F1 male mice. In total, an average of 156 610 reads per sample were generated.

**Fig. 3:**
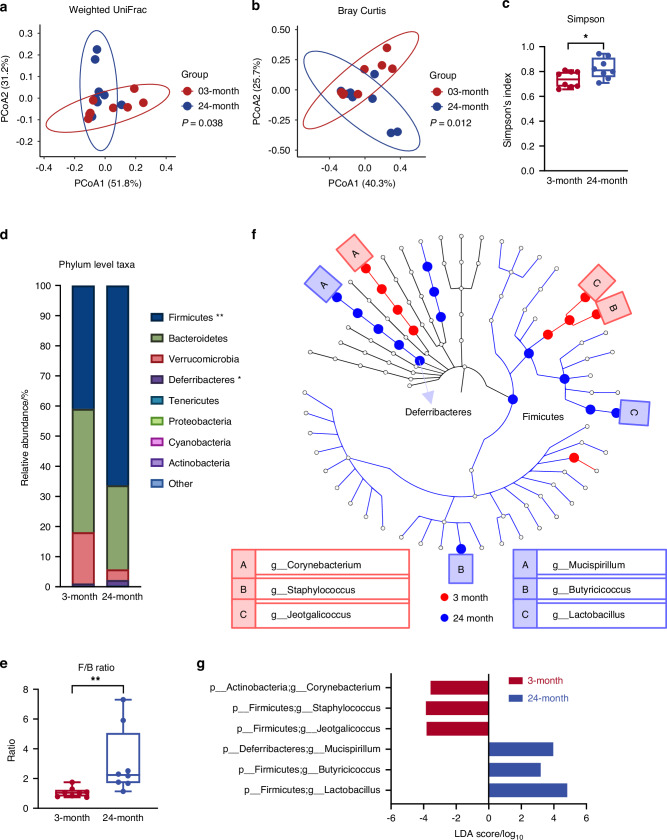
Distinct microbial compositions between young and old male mice. Principal coordinate analysis (PCoA) plots of (**a**) weighted UniFrac distance and (**b**) Bray Curtis distance were used to visualize microbial β-diversities. Significance was determined by permutational multivariate analysis of variance (PERMANOVA) with 999 permutations, (**a**, **b**) showing a significant microbial community shift between the young (red dots) and old (blue dots) mice. **c** α-diversity was measured by Simpson index. **d** Mean relative abundance of taxa at phylum level. **e** Firmicutes to Bacteroidetes (F/B) ratio was calculated. **f** Cladogram of Linear discriminant Effect Size (LEfSe) analysis. **g** At the genus level, 6 discriminant taxa were identified by LEfSe analysis. Mann Whitney U test was performed for (**c–e**). For (**d**), *P < 0.05, **P < 0.01. 3-month, *n* = 8; 24-month, *n* = 8

Principal coordinate analysis was performed on the β-diversity distance matrices, which assessed the microbial communities’ dissimilarity between samples. Quantitative matrices which consider taxa abundance (weighted UniFrac and Bray-Curtis,) and qualitative matrices which consider presence/absence patterns of taxa (unweighted UniFrac and Jaccard) were calculated, respectively. Accordingly, a significant age-dependent shift of gut microbiome was found between the young and old mice based on the weighted UniFrac and Bray-Curtis distance matrices (PERMANOVA with 999 permutations, *P* = 0.038 for weighted UniFrac and *P* = 0.012 for Bray-Curtis, Fig. 3a, b). However, no statistical differences were found based on the unweighted UniFrac and Jaccard distance matrices (Fig. S3a). Therefore, age-dependent microbial structure divergence is predominantly driven by the changes in taxa abundance rather than the presence/absence patterns of taxa.

The α-diversity indices Shannon, Simpson and Chao1 were calculated after rarefaction (Fig. S3b) to estimate the microbial richness (number of taxa) and evenness (distribution of taxa relative abundances). The Simpson index was significantly increased in the old mice (Fig. 3c), while no alteration of Shannon and Chao1 indices were found between the young and old mice (Fig. S3c). Given that the calculation of Simpson index places more weight on microbial evenness than richness in comparison to the other two indices, our results suggested that an age-dependent increase of microbial α-diversity was likely due to a greater evenness in the microbiome of aged mice. Collectively, CB6F1 male mice showed altered microbial diversities in response to aging similar to what we previously reported in C57BL/6 mice.^23^

### Distinct microbial compositions between young and old male mice

At the phylum level, Firmicutes and Bacteroidetes were the two most abundant taxa in both young and old male mice, constituting 51.5%–99.8% amplicon sequence variants (ASVs) identified in the 16 mice fecal samples (Fig. 3d and Table S1). The relative abundance of Firmicutes was significantly increased in old mice (young: 40.93% ± 3.03% vs. old: 66.30% ± 4.45%, Fig. 3d and Table S1), leading to an increased ratio of Firmicutes to Bacteroidetes (F/B ratio) in the old mice (young: 1.07% ± 0.12% vs. old: 3.10% ± 1.79%, Fig. 3e). The increased relative abundance of Firmicutes in old mice was mostly attributed to age-dependent increases of *Lactobacillus* (young: 9.91% ± 1.21%, old: 24.12% ± 3.69%) and *Butyricicoccus* (young: 0.04% ± 0.01%, old: 0.16% ± 0.05%) at genus level (Table S2 and Fig. S3d). Both taxa were identified as discriminant taxa for the microbiome from the old mice by LefSe analysis (Fig. 3f, g and Fig. S3e).

The relative abundance of subdominant taxon Deferribacteres was also significantly increased in the old mice (young: 0.78% ± 0.08%, old: 1.84% ± 1.55%, *P* = 0.038, Fig. 3d and Table S4). This was primarily driven by variation of *Mucispirillum* at the genus level (young: 0.78% ± 0.08%, old: 1.84% ± 1.55%, Table S2 and Fig. S3d), which was also identified as a significant discriminant taxon for the old mice microbiome by LefSe analysis (Fig. 3f, g and Fig. S3e). On the other hand, 3 taxa at the genus level including *Jeotgalicoccus, Staphylococcus* and *Corynebacterium* were significantly enriched in the young mice (Table S2 and Fig. S3d) and were identified as significant discriminant taxa for the young mice microbiome by LefSe analysis (Fig. 3f, g and Fig. S3e). Taken together, the young and old CB6F1 male mice demonstrated distinct microbial communities.

### Age-dependent variation in predicted energy generation and nutrient metabolisms by microbial populations

The metagenomic function was predicted by PICRUSt2 based on 16S rRNA gene sequencing data. In total, 104 pathways were significantly altered between the young and old male mice, of which 44 pathways were down-regulated while 60 pathways were up-regulated in old mice compared to young mice (Table S3).

**Fig. 4:**
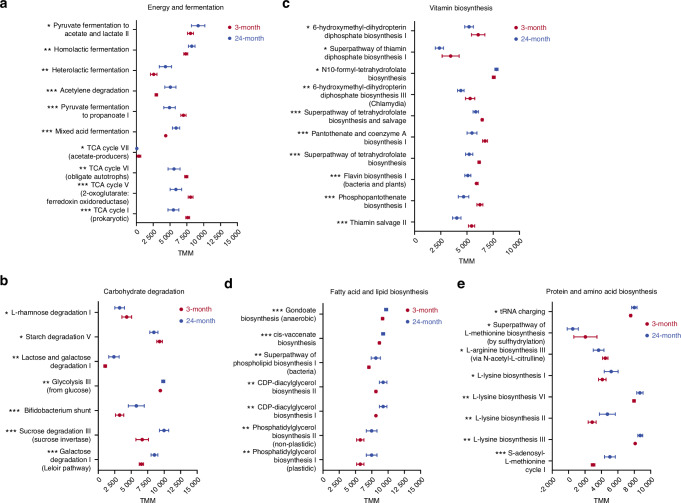
PICRUSt2 predictions of microbial metagenome functions based on 16S rRNA gene. Metagenomic function was predicted using Phylogenetic Investigation of Communities by Reconstruction of Unobserved States (PICRUSt). Young and old mice demonstrated different microbial predicted metagenomic functions in (**a**) energy and fermentation, (**b**) carbohydrate degradation, (**c**) vitamin biosynthesis, (**d**) fatty acid and lipid biosynthesis, and (**e**) protein and amino acid biosynthesis. Data are TMM (trimmed mean of *M* values) normalized and represented as mean with 95% confidence intervals. Unpaired *t*-test or Mann Whitney *U* test was performed. *P < 0.05, **P < 0.01, ***P < 0.001. 3-month, *n* = 8; 24-month, *n* = 8

Old mice had lower levels of four TCA cycle-related pathways, indicating reduced energy potential in their microbiome. The pyruvate fermentation to propanoate pathway was also lower in old mice (Fig. 4a). Conversely, several microbial fermentation pathways producing lactate, acetate, and ethanol were higher in old mice (Fig. 4a), suggesting potential differences in microbial fermentation products between young and old mice.

Microbial carbohydrate degradation also changed significantly in response to aging (Fig. 4b). Old mice had increased mono- and di-saccharide degradation pathways, including lactose, galactose, glucose, and sucrose degradation. Young mice, on the other hand, showed an increase in starch and rhamnose degradation (Fig. 4b).

Significant underrepresentation of microbial B vitamin biosynthesis and metabolism (Fig. 4c) including thiamine (B1), riboflavin (B2), pantothenate (B5), and folate (B9) was also seen in old mice. Conversely, microbial phospholipid biosynthesis and unsaturated fatty acid biosynthesis were significantly higher in the old mice compared to the young mice (Fig. 4d). Moreover, microbial tRNA charging involved in protein biosynthesis, lysine biosynthesis and S-adenosyl-L-methionine (SAM) cycle were enriched in old mice (Fig. 4e). Taken together, aging significantly affected predicted microbial energy generation and nutrient metabolism.

### Distinct fecal metabolomic profiles between the young and old male mice

Microbial metabolic compounds are widely thought to mediate host-microbiome interactions. Given that both compositional and functional alterations in response to aging were observed, we next performed LC-MS to characterize the fecal metabolome and confirm that these aging associated changes in microbiome result in metabolic functional consequences.

**Fig. 5:**
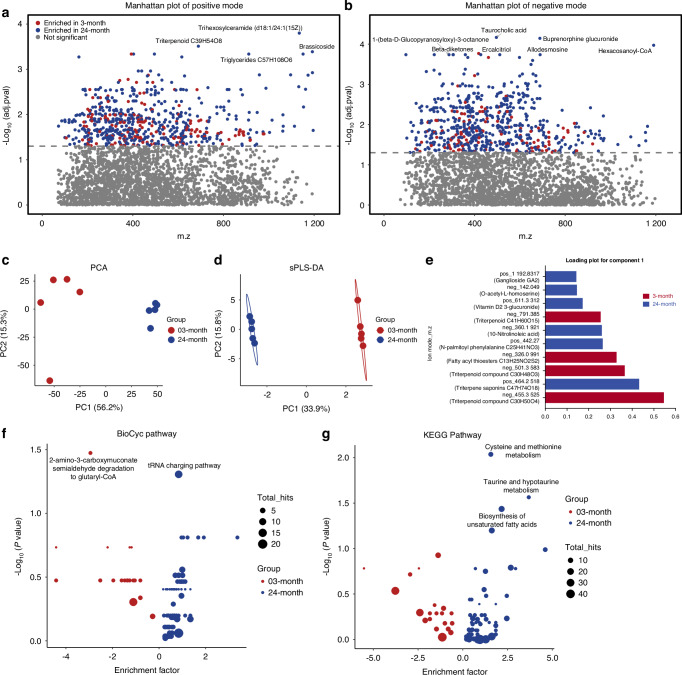
Distinct fecal metabolite profiles between young and old mice. Manhattan plots were used to visualize the significantly differentially expressed spectral features obtained from MS (**a**) positive mode and (**b**) negative mode. Red dots and blue dots represent spectral features significantly enriched in the young and old mice, respectively (adjusted *P* < 0.05). **c** Principal component analysis (PCA) plot shows two clearly separate clusters according to fecal donor age. **d** Supervised sparse partial least square discriminant analysis (sPLS-DA) was performed to select most discriminative features to classify young and old mouse fecal metabolomes. The optimal complexity of the sPLS-DA model was assessed by five-fold cross-validation. The top 10 spectral features were selected for loading plot. **e** Candidate metabolites associated with these spectral features are annotated in brackets. Bubble plots were used to visualize the pathway enrichment analysis performed by Metaboanalyst 5.0 using the (**f**) *Mus musculus* BioCyc and (**g**) *Mus musculus* KEGG databases. X-axis represented enrichment factor (the ratio of the number of significant metabolic features to the expected number within the pathway). Y-axis represented -log_10_ (*P* value). Size of the bubbles represents the total metabolic features hitting within the pathway. Significantly enriched pathways were annotated (*P* < 0.05). 3-month, *n* = 5; 24-month, *n* = 5

A total of 3 069 metabolic features from positive ion mode and 2 877 metabolic features from negative ion mode were annotated by HMDB in 10 fecal samples (5 per age group). 1 288 metabolic features were significantly differentially expressed, of which 1 012 metabolic features were significantly increased and 276 were significantly decreased in the fecal samples from the old mice compared to the young mice (Fig. 5a, b).

A PCA plot showed two clearly separate clusters according to the age of the mice, indicating distinct metabolomic profiles between the young and old mice fecal samples (Fig. 5c). Supervised sparse partial least discriminant analysis (sPLS-DA) was further applied (Fig. 5d). As assessed by five-fold cross-validation, the optimal complexity of the model was found to be one component with 10 features selected, resulting in 0% classification error rate (Fig. 5e). 4 of the 10 features responsible for discrimination were enriched in young mice while the remaining 6 features were enriched in old mice (Fig. 5e). The 10 LC-MS peaks annotated with all potential HMDB IDs and compounds are listed in Table S4.

Pathway enrichment analysis was performed on differentially expressed metabolic features by MetaboAnalyst 5.0 to further elucidate microbial metabolic alterations in response to aging. Consistent with the results of the predicted metagenomic functions, pathway enrichment analysis also showed significant increases in unsaturated fatty acid biosynthesis and tRNA charging pathways in the metabolome of old mice. Moreover, cysteine and methionine metabolism and taurine and hypotaurine metabolism were also significantly enriched in old mice (Fig. 5f, g). On the other hand, young mice featured increases in pathways involved in tryptophan degradation (2-amino-3-carboxymuconate semialdehyde degradation to glutaryl-CoA, Fig. 5f).

### Correlation of fecal metabolites and 16S predicted metagenome

The metabolites derived from 16S predicted pathways were annotated and compared to the LC-MS fecal metabolome (Table S5,S6). 152 metabolic features from fecal metabolome matching 16S predicted pathways were identified, of which 59 metabolic features were identified as significantly altered in response to aging in analysis of both metabolome and predicted metagenome (Table S7 and Fig. S3f).

**Fig. 6:**
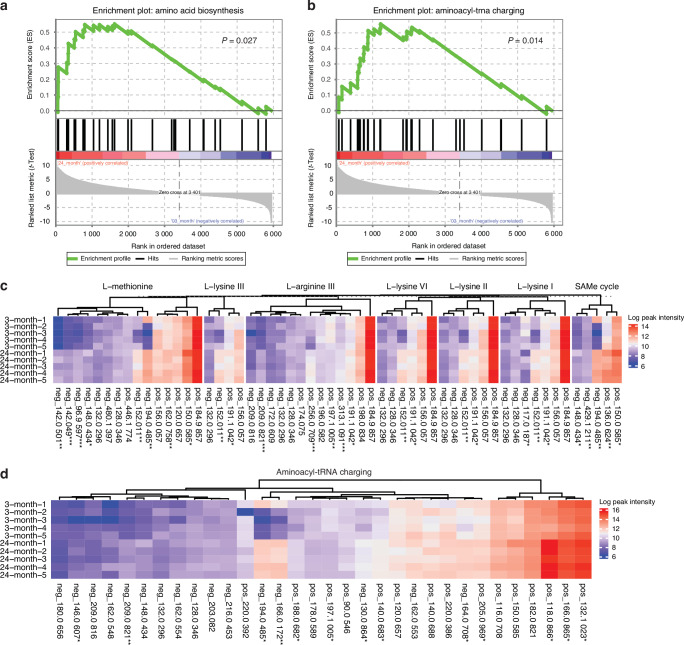
Integrative analysis of 16S microbiome and LC-MS metabolome. Integrative analysis of 16S microbiome and LC-MS metabolome was performed using a customized gene set enrichment analysis (GSEA) method. Metabolites obtained by LC-MS were significantly enriched in (**a**) amino acid biosynthesis and (**b**) aminoacyl-tRNA charging using the predicted metagenome of 16S rRNA gene dataset (*P* < 0.05). Peak intensity of metabolites involved in (**c**) amino acid biosynthesis and (**d**) aminoacyl-tRNA charging was log transformed and visualized by heatmap, with each row representing a unique sample and each column representing a LC-MS peak. For (**c**, **d**), *P < 0.05, **P < 0.01, ***P < 0.001. For LC-MS, 3-month, *n* = 5; 24-month, *n* = 5. For 16S, 3-month, *n* = 8; 24-month, *n* = 8

An integrative analysis of 16S predicted metagenome and LC-MS metabolome was performed by a customized gene set enrichment analysis (GSEA). Metabolites obtained by LC-MS were first classified into 16S predicted pathways at a higher superclass level. GSEA was then performed to test whether the pathways representing this set of metabolites were significantly enriched between young and old mice. Amino acid biosynthesis and aminoacyl-tRNA charging were significantly enriched in old mice by integrative analysis (Fig. 6a–d), consistent with the 16S predicted functional analysis (Fig. 4e) and the LC-MS pathway enrichment analysis (Fig. 5f, g). Together, these data suggest that alterations in microbial protein and amino acids biosynthesis could be a distinguishing feature for aging in CB6F1 male mice.

### Effect of microbial colonization on bone phenotypes of germ-free mice is independent of age of the fecal donors

We previously showed that the effect of microbial colonization on bone homeostasis varied depending on the duration of colonization. Short-term colonization of gut microbiome resulted in a reduction in trabecular bone mass whereas prolonged colonization promoted longitudinal bone growth.^7^ In this study, we were interested in whether the effect of microbial colonization on bone phenotypes varied according to the age of fecal donors. Accordingly, GF mice were reconstituted with fecal microbiome from young (3-month-old) or old (24-month-old) male donor mice. Fecal and femur samples were collected and assessed after short-term (1 month) and long-term (8 months) colonization.

The fecal microbiome of the recipient mice was analyzed by qRT-PCR to investigate whether age-related microbial divergence persisted in the gnotobiotic recipients. Mice colonized with microbiome from 24-month-old donors exhibited higher total bacterial abundance and a significantly increased abundance of Bacteroidetes compared to those colonized with microbiome from 3-month-old donors, both in the short-term and long-term colonization cohorts (Fig. S3g, h). Additionally, Deferribacteres, Actinobacteria, and γ/δ-Proteobacteria also had similar trend of alteration between mice colonized with microbiome from different ages of donors in both cohorts (Fig. S3g, h). However, *Lactobacillus*, the primary genus driving the increased Firmicutes in the old donor mice, showed no or very low abundance in the colonized mice with either young or old donors’ microbiome, indicating microbial loss during FMT (data not shown). Consequently, there was no difference in Firmicutes between the colonized mice from different donor ages (Fig. S3g, h). Despite the microbial loss, microbial divergence persisted post-FMT in the gnotobiotic mice colonized with microbiomes from donors of different ages.

**Fig. 7:**
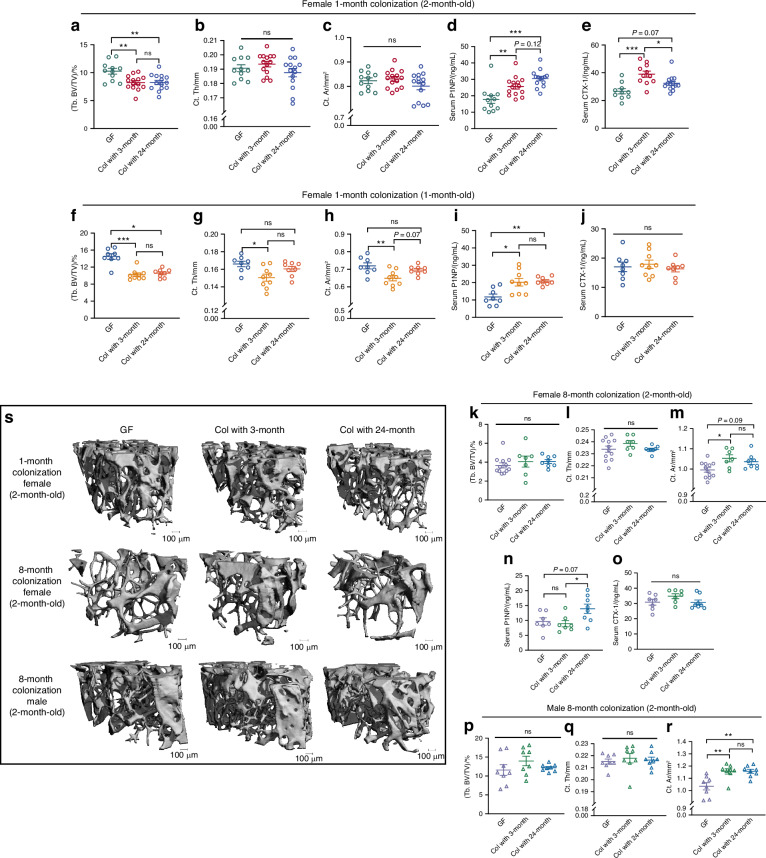
Effect of colonization on bone phenotype of germ-free mice is independent of age of donor microbiome. Effect of colonization for 1 month on trabecular bone volume fraction (Tb. BV/TV), cortical thickness (Ct. Th), cortical area (Ct. Ar), serum P1NP and CTX-1 in young adult (2-month-old, **a–e**) and skeletal immature (1-month-old, **f–j**) females is shown. Consequences of 8 months colonization on Tb. BV/TV, Ct. Th, and Ct. Ar are shown for (**k**–**m**) female and (**p**–**r**) male mice. **n**, **o** Serum P1NP and CTX-1 is shown in female after 8 months colonization. **s** Representative 3D images of femur trabecular bone are shown. Data are represented as mean ± SEM. One-way ANOVA or Kruskal-Wallis test with Tukey post hoc was performed. *P < 0.05, **P < 0.01, ***P < 0.001, ns, not statistically significant. For (**a–e**): GF, *n* = 11; Col with 3-month, *n* = 14; Col with 24-month, *n* = 14. For (**f–j**): GF = 8; Col with 3-month, *n* = 9; Col with 24-month, *n* = 8. For (**k–o**): GF, *n* = 12; Col with 3-month, *n* = 7; Col with 24-month, *n* = 8. For (**p–r**): *n* = 8 per group. 4 data in (**e**) and 5 data in (**n**) and (**o**) were missing due to insufficient sample collection

After 1 month colonization, Tb. BV/TV exhibited a significant decrease in female colonized mice when compared to GF mice. This reduction was observed regardless of whether they were colonized with microbiome from 3-month-old or 24-month-old donors. (GF:10.26% ± 0.52%, 3-month: 8.22% ± 0.34%, 24-month: 8.25% ± 0.38%, Fig. 7a), indicating an overall acute catabolic effect of microbiome colonization on bone mass.

However, no significant differences were seen between female mice colonized with young compared to old donors’ microbiome. Distal femur bone parameters, including bone volume fraction (Tb. BV/TV), trabecular number (Tb. N), trabecular thickness (Tb. Th), trabecular spacing (Tb. Sp), cortical thickness (Ct. Th), cortical area (Ct. Ar), periosteal perimeter (Ps. Pm) and tissue mineral density (Ct. TMD) were similar between groups (Fig. 7a–c and Fig. S4a–e). We also observed no impact of microbial donor age in a second independent cohort in which female GF mice were colonized at 1-month-old to test whether differences would be more apparent during a period of rapid skeletal growth for recipient mice (Fig. 7f–h and Fig. S4f–j).

We previously found that short-term colonization of microbiome in GF mice increased P1NP.^7^ Consistent with prior results, serum P1NP in this study showed a significant increase by microbial colonization in GF females in both short-term cohorts colonized at 1 month and 2 months old (Fig. 7d, i). However, donor age had no significant impact on serum P1NP. Serum CTX-1 was significantly increased in 2-month-old GF female mice colonized with microbiomes from young donors, but not from old donors (Fig. 7e). No alteration of serum CTX-1 was found when 1-month-old GF mice were colonized with microbiome from either age of donors (Fig. 7j). While systemic bone catabolism may be affected by the age of the donor and the age at colonization, short-term colonization with either young or old donors’ microbiome resulted in a similar effect on bone phenotypes in female mice.

Next, we investigated whether a prolonged period of microbial colonization from donors of different ages would result in distinct bone phenotypes. Here we examined the impact of fecal donor age on both male and female GF mice. Similar to the results of the short-term colonization, no significant difference was observed in bone phenotypes between mice colonized with microbiome from young compared to old donors. Results were similar in male and female mice (Fig. 7k–m, p–r and Fig. S5). Serum P1NP was significantly increased in GF female mice colonized with microbiome from old donors, but microbiome from young donors had no effect on P1NP (Fig. 7n), nor was there a difference in serum CTX-1 between GF and colonized mice, regardless of donor age (Fig. 7o).

Taken together, our findings show acute loss of femoral trabecular bone volume fraction after microbial colonization of the gut with recovery after 8 months post-transplant. The age of the fecal donor does not affect the impact of microbial colonization of GF mice on bone phenotypes. However, we cannot rule out that donors age, the colonization age and the colonization period may differently affect systemic bone metabolism.

## Discussion

In this study, we investigated the role of age-related alterations in gut microbiome in bone loss with aging. We used CB6F1 mice, a strain with a relatively long lifespan frequently used in aging studies. To examine the importance of microbiome for age-related bone loss, we compared age-related bone loss in GF mice and their colonized littermates. At 24-months of age, GF mice demonstrated similar trabecular bone mass as compared to their colonized littermates. Moreover, bone loss from 3 to 24-month-old was comparable between GF and specific pathogen-free (SPF) mice, indicating GF mice were not protected from age-related bone loss. Thus, our study showed that bone loss with aging is independent of gut microbiome in CB6F1 mice.

The gut microbiome of the aged CB6F1 mice demonstrated increased α-diversity and distinct fecal microbial structures (β-diversity) compared to that of the young mice. To determine whether the changes in the composition of gut microbiome corresponded to alterations in microbial functions, we performed microbial metagenomic functional predictions by PICRUSTs and metabolomic profiling by LC-MS. As expected, distinct microbial predicted functions and metabolic consequences were observed between young and old mice. Notably, both the individual datasets and the integrative analysis revealed enrichment of tRNA charging pathways, responsible for loading tRNAs with amino acids for use in protein synthesis, in aged mice. This raises the possibility that increased amino acid flux into microbial protein synthesis ultimately affects host amino acid balance in the aged CB6F1 mice.^29^ The S-adenosyl-L-methionine (SAMe) cycle was also enriched in aged mice. SAMe serves as a methyl donor for DNA and protein methylation, which is critical in the regulation of many biological processes. Microbial SAMe synthesis is implicated in the host aging process by its essential function in metformin-induced extension of lifespan in *Caenorhabditis elegans* (*C. elegans*).^30^ Collectively, an age-dependent divergence in microbial protein and amino acid metabolism potentially plays a role in the host nitrogen balance and aging process.

Despite microbial compositional and functional alteration with aging, GF mice reconstituted with the microbiome of young and old donors exhibited comparable bone phenotypes after colonization for either 1 or 8 months, suggesting that the effect of colonization on bone phenotypes was independent of fecal donor age. It is important to note that the current fecal microbiome transfer techniques cannot ensure the successful transfer of all the taxa identified by 16S rRNA gene sequencing as 16S rRNA gene sequencing identifies both live and dead microbial populations. Moreover, some microbes may be lost during the FMT procedure. Furthermore, recipient host and environmental factors could lead to microbiome drift post-FMT. In the current study, the brand of chow diet fed to recipient mice differed from the donor diet, and may also contribute to microbiome drift post-FMT. However, physical phenotypes, such as obesity and skeletal maturation, can be transferred from donors to recipients by fecal transplantation, indicating the capacity of gnotobiotic mice to successfully maintain specific microbiome associated with donors’ phenotypes.^31,32^ Gut microbiome development and final composition are largely influenced by the pioneering colonizers, known as “priority effects”.^33^ Such “microbial priority effect” may overweigh other factors to determine the microbial effect on host health. For example, FMT from calorie restricted (CR) mice to diet induced obese (DIO) mice demonstrate metabolic improvement despite continued exposure to high fat diet.^34^ In this study, we used qRT-PCR to confirm the success of FMT, a technique commonly used to quantify microbial load and profile gut microbiome community.^35^ Despite the loss of some microbes and the potential microbial drift, the microbial divergence persisted between the recipient mice colonized with different ages of donors’ microbiome throughout the duration of our experiment.

While we chose to use the same batch of pooled male fecal samples for colonizing GF mice in order to control for potential confounding due to interindividual variations in microbiome, this did limit our ability to investigate sex differences of donors’ microbiome on the consequences of FMT, the potential of sex-dependent difference for microbiome drift after FMT, and their impact on bone phenotypes. However, this is a minor limitation as gut microbial functions have been shown to be highly conserved between female and male mice despite taxonomic differences,^36^ and our previous meta-analysis found comparable age-related microbial functional changes between female and male C57BL/6 mice.^23^

Although we find that age-related bone loss in mice occurs in the absence of a gut microbiome, this does not rule out an important role for microbiome to either positively or negatively impact bone loss under particular conditions, for example ovariectomy, or to impact bone material properties.^8,9,37^ Furthermore, unlike mice in which diet is tightly controlled, in real-life situations, aged individuals are likely to encounter changes in diet, nutrient supplementations, and medication intake. These factors can influence gut microbial composition and function, which in turn can impact host bone phenotypes. For example, gut microbiome dysbiosis induced by low glycemic diet and antibiotics resulted in a reduction in bone tissue strength in aged mice.^8^ Additionally, *Lactobacillus* supplementation attenuated age-related bone loss in both animal studies and human trials.^38,39^ Various human aging studies indicate multiple gut microbiome patterns of aging exist, resulting from the variations in health conditions, diet, physical activities, medication intake, and housing environments.^18–22^ It remains intriguing to investigate whether the modifications to gut microbiome by external interventions could impact age-related bone loss.

In summary, our study demonstrated age-related changes in the compositions and functions of the gut microbiome in CB6F1 mice. However, age-related microbial alteration did not appear to play a significant role in the process of bone loss with aging in our gnotobiotic mouse model. It is important to note that the age-related microbial changes in humans are more complex than those observed in mice. Future research may benefit from using humanized gnotobiotic mice models to investigate the potential effects of the different human aging microbial patterns on age-related bone loss.

## Materials and methods

### Animals

CB6F1 germ-free mice were generated from female BALB/c and male C57BL/6 mice and housed in germ-free Trexler isolators (Alpha-dri paper-based bedding). GF mice were transferred to individually ventilated cages at a BSL2 cubicle when fecal microbiome colonization occurred (3-5 mice per cage, Andersons ¼” Bed-o’Cobs corncob bedding). The housing environmental conditions were maintained at constant room temperature (22 °C ± 10%), air humidity (50% ± 20%), and a light/dark cycle of 12 h. The gnotobiotic mice experiments were conducted in the National Gnotobiotic Rodent Resource Center, University of North Carolina at Chapel Hill.

3-month-old and 24-month-old CB6F1 conventional SPF mice were obtained from the National Institute on Aging (NIA) and housed under SPF conditions (Alpha Chip softwood pine bedding) at constant room temperature (22 °C ± 10%), air humidity (50% ± 20%), and a light/dark cycle of 12 h. The SPF conventional mice study was conducted in the Brigham and Women’s Hospital/Harvard Medical School vivarium.

All mice were given ad libitum access to autoclaved water and chow diet (5053 PicoLab Mouse Diet 20 for SPF mice with 24.5% protein, 13.1% fat and 63.4% carbohydrate by calorie density, and 4.4% crude fiber and 2020SX Envigo for gnotobiotic mice with 24% protein, 16% fat, 60% carbohydrate by calorie density, and 2.7% crude fiber).

For fecal sample collection, each cage housed 4-5 conventional SPF male mice, with a total of 4 cages per age group. Two to four fecal pellets were freshly collected from each individual mice, flash frozen and pooled together within their respective cages and stored at −80 °C until analysis. Two fecal pellets from each cage were randomly selected (a total of 8 fecal samples per age group) for used for 16S rRNA gene sequencing. One to two fecal pellets from each cage were randomly selected (a total of 5 fecal samples per age group) for fecal LC-MS metabolomic analysis. Colonization of GF mice was performed as follows: 10 fecal samples per age group were pooled from 4 cages of SPF donors and homogenized in 5 mL sterile PBS for colonization purpose. Sterile cotton swabs were used to inoculate the mouth and anus with fecal material and wiped on the abdomen of the mouse. Fecal slurry was prepared freshly in an anerobic chamber at the time of colonization.

For fecal microbiome transplant (FMT) experiments, 4 independent cohorts of mice were utilized (Fig. 1). 1-month-old GF mice were randomly assigned to 2 experimental groups: remain GF (a) or colonization with microbiome from 3-month-old donors for 23 months (b) (Cohort 1, Fig. 1a).

In a second cohort (Fig. 1c), 2-month-old CB6F1 GF mice were randomly assigned to remain GF (a) or be colonized with fecal microbiome from 3-month-old (b) or 24-month-old donors (c) for 1 month.

The third cohort (Fig. 1d) tested the effect of colonization prior to skeletal maturity by comparing 1-month-old CB6F1 GF mice randomized to remain GF (a) or be colonized with microbiome from 3-month-old (b) or 24-month-old donors (c) for 1 month.

Cohort 4 (Fig. 1e) was set up identically to cohort 2 (Fig. 1c), but the duration of colonization was extended to 8 months. Cohort 1 and cohort 4 were performed in both female and male mice. Cohort 2 and cohort 3 were performed in female mice.

In addition to the four FMT cohorts, bone phenotypes of 3-month-old and 24-month-old GF mice were compared to age-matched conventionally raised SPF mice (Fig. S2).

All animal procedures were approved by the Institutional Animal Care and Use Committee, Harvard Medical School, and the University of North Carolina at Chapel Hill for the gnotobiotic experiments. GF mice and colonized mice with 3-month-old microbiome at 2-month-old in Fig. 7 are the same groups of mice that were reported previously.^7^ In the current study, these two groups of mice were reanalyzed and compared to the colonized mice with 24-month-old microbiome that were collected in parallel but not previously reported.

### Micro-CT analysis

Femurs were collected at the time of mice sacrifice and fixed in 4% paraformaldehyde in PBS for 24 h followed by 70% (v/v) ethanol. μCT 35 (Scanco Medical AG) was used to scan femur samples in 70% ethanol using a 7 µm voxel size and the following settings: X-ray tube potential of 55 kVp, intensity of 145 mA, and integration time of 600 ms. Trabecular bone region was selected starting 0.35 mm proximal to the growth plate and extending 1 mm proximally. Cortical bone was assessed at the midshaft (0.6 mm in length). Global threshold values of 495.03 mg HA/cm^3^ for trabecular bone and 760.05 mg HA/cm^3^ for cortical bone were selected based on an historical experience and confirmation by an experienced reader.

### Serum P1NP and CTX-1 measurement

Blood samples were collected by cardiac puncture. Serum samples were separated from blood by using serum separation tubes (BD Medical, NJ, US). Serum PINP and CTX-1 were measured by Rat/Mouse PINP EIA kit (IDS, UK) and RatLaps CTX-1 EIA kit (IDS, UK) following the manufacturer’s protocol.

### Scanning electron micrography

Tibia were dissected, cleaned from soft tissue, fixed in 70% ethanol for 48 h and dehydrated in solutions with increasing concentrations of ethanol (80%, 90%, 100%, 24 h in each solution). Samples were kept in Xylene (Thermo Fisher Scientific, MA, US) for 24 h and destabilized methyl methacrylate (MMA, Sigma-Aldrich, MO, US) for 48 h. Each sample was placed in a glass vial filled with DMMA and 2% benzoyl peroxide (Sigma-Aldrich, MO, US), infiltrated for 48 h and left for 10 days at the room temperature to polymerize. After solidification, glass vials were broken to access the blocks of the PMMA containing bone samples. Each block was cut transversely and mid-diaphysis part of the bone, using a low speed saw (IsoMet 1000 Precision Saw, Buehler, Braunschweig, Germany) equipped with a 500 micron thick diamond blade (Buehler, Braunschweig, Germany). Surface of each sample was polished with succeeding finer grades of the alumina powder (1 mm, 0.3 mm and 0.05 mm) and cleaned using an ultrasound bath between the polishing. To eliminate electron charging, samples were coated with carbon and connected to the edge of the sample holder at three locations with carbon adhesion. Samples were scanned using a digital electron microscope with a four-quadrant semiconductor backscatter detector (Ziess Evo, Oberkochen, Germany). Imaging was performed at a 20 kV accelerating voltage, saturated filament current, 1.5 nA probe current measured with a Faraday cup and at a working distance of 12 mm. Magnification settings, store resolution and the scan speed were kept consistent between different imaging sessions, such that the pixel size was always 1.5 mm for every BSE image. For calibrating backscattered signal, pure carbon and aluminum standards (Micro-Analysis, Huntingdon, UK) were scanned at the same imaging condition as the bone during every imaging session. To increase the dynamic range of the bone mineralization at different parts of the sample, brightness and contrast were adapted such that gray level number of carbon and aluminum were as close as possible to 25 and 225, respectively. Calibration standards were scanned before and after each specimen. In a post-processing analysis in MATLAB (Mathworks, MA, US) and to account for variations of the electron beam during the scan, gray level number of the standards were averaged between these two scans and used for calibrating the bone specimen. Eventually, gray level number of the bone and phantom were expanded linearly such that carbon and aluminum were 25 and 225, respectively. From each gray-scale image, a histogram of graylevel numbers was generated. Two parameters were evaluated from the histograms including mode and full width at half maximum (FWHM). Mode represents the most common degree of mineralization in the bone (peak of the histogram) and FWHM indicates the heterogeneity in mineralization. Data reproducibility was checked by scanning the same sample in different imaging sessions and obtaining identical data. A custom MATLAB code (Mathworks, MA, US) was used for quantifying matrix porosity from the graylevel qbSE images. In this analysis pores with diameter greater than 15 micron were considered as vascular and smaller pores were categorized as lacunar porosity.

### Fecal DNA extraction and 16S rRNA gene sequencing

Total genomic DNA was extracted from fecal pellets with MO BIO PowerFecal DNA isolation kit according to the manufacture’s protocol with the addition of a bead-beating step. Fecal DNA concentration was measured by Quant-IT dsDNA high sensitivity assay. PCR was performed to amplify 16S rRNA gene V4 region with universal bacterial/archaeal primers (515 F: AATGATACGGCGACCACCGAGATCTACACNNNNNNNNTATGGTAATTGTGTGCCAGCMGCCGCGGTAA and 806 R: CAAGCAGAAGACGGCATACGAGATNNNNNNNNAGTCAGTCAGCCGGACTACHVGGGTWTCTAAT). The size of DNA amplicons was analyzed on an Agilent Technologies 2100 bioanalyzer trace. DNA concentration of the aggregated library was measured by the Quant-IT dsDNA high sensitivity assay. The DNA in the library was denatured by NaOH and diluted to 7.5 pmol·L^−1^ with HT buffer provided in the Illumina kit. 600 μL of the denatured and diluted library with 20% phiX spike-in (120 μL, 7.5 pmol·L^−1^ of PhiX) was loaded onto the MiSeq V2 reagent cartridge (Illumina) prior to 2 × 250 paired-end sequencing on Illumina Miseq platform. Sequencing was performed at BWH Massachusetts Host - Microbiome Center.

### 16S taxonomic and predicted metagenomic function analyses

Raw FASTQ files were analyzed by QIIME2 v2019.10.^40^ The demultiplexed reads were quality filtered, barcode trimmed, and chimera detected by q2-vsearch plugin. Amplicon sequence variants (ASVs) were assigned by clustering sequence reads with 99% identity against the Greengenes V13-8 database. Diversity metrics (Shannon, Simpson and Chao1 for α-diversities and weighted UniFrac, unweighted UniFac, Bray-Curtis, Jaccard for β-diversities) were calculated by R package phyloseq. Relative abundance of each taxon was calculated by dividing the number of reads by the total reads of the sample. Any unassigned ASVs at the genus level were grouped to the possible family/order level for downstream analysis.

Metagenomic function was predicted using Phylogenetic Investigation of Communities by Reconstruction of Unobserved States (PICRUSt2).^41^ ASV table was normalized by the known/predicted 16S copy number abundance. The functional trait abundance was then predicted within PICRUSt2 followed by trimmed mean of *M*-values (TMM) normalization in R package edgeR^42^ for further statistical analysis.

Linear discriminant analysis effect size (LEfSe) was performed to determine the enriched taxa of each age group and visualized by cladogram by GraPhlAn in the Galaxy web server.^43^ LEfSe couples a univariate nonparametric test for statistical significance with post hoc prioritization by the size of the effect as determined by linear discriminant analysis (LDA). Mann Whitney *U* test was used to analyze all features. *P* < 0.05 with logarithmic LDA score >2 was used for the identification of discriminant features.

The 16S rRNA raw sequencing data has been deposited in the NCBI SRA under accession numbers PRJNA737742. This dataset was previously used as the external verification dataset for previously published random forest models characterizing age-related changes in gut microbiome.^23^

### Fecal microbiome analysis by qRT-PCR

Fecal samples were freshly collected from recipient mice 1-month and 8-month after fecal microbiome transfer (FMT), flash frozen and stored at −80 °C until analysis. Total fecal genomic DNA was extracted by QIAamp PowerFecal Pro DNA Kit (Qiagen) according to the manufacture’s protocol. Total bacteria abundance and specific taxa abundance of Firmicutes, Bacteroidetes, Deferribacteres, Actinobacteria, and γ/δ-Proteobacteria were quantified by quantitative real-time PCR (qRT-PCR) using Fast SYBR green master mix (Applied Biosystems) on a StepOne Plus real-time PCR machine (Applied Biosystems). The following primer sets were used to quantify total bacteria abundance: Uni-340F (ACTCCTACGGGAGGCAGCAGT) and Uni-514R (ATTACCGCGGCTGCTGGC), Firmicutes: Firm-928F (TGAAACTYAAAGGAATTGACG) and Firm-1040R (ACCATGCACCACCTGTC), Bacteroidetes: CBF-798F (CRAACAGGATTAGATACCCT) and CBF-967R (GGTAAGGTTCCTCGCGTAT), Deferribacteres: Defer-1115F (CTATTTCCAGTTGCTAACGG) and Defer-1265R (GAGHTGCTTCCCTCTGATTATG), Actinobacteria: Act-664F (TGTAGCGGTGGAATGCGC) and Act-941R (AATTAAGCCACATGCTCCGCT), γ/δ-Proteobacteria: Gamma-887F (GCTAACGCATTAAGTRYCCCG) and Gamma-1066R (GCCATGCRGCACCTGTCT).^7,35,44^ The primer efficiency of each primer set was determined as previously described.^35^ A standard curve was generated from a series dilution of genomic DNA extracted from an overnight culture of *Lactobacillus plantarum* which was quantified by standard plate count. The total fecal bacteria abundance was calculated from the standard curve with the consideration of input fecal weight. Specific taxa were calculated using the following formula X = [(Eff.uni)^CT.uni^ / (Eff.spec) ^CT.sepc^] × total bacteria abundance, where “X” represents the abundance of the specific taxon of interest, “Eff.uni” is the primer efficiency for the universal bacteria primers (Uni-340F and Uni-514R), “Eff.spec” is the primer efficiency for the specific taxon, “CT.uni” and “CT.spec” are the Ct values obtained from qRT-PCR reactions.

### Fecal LC-MS metabolome analysis

Second aliquot of fecal samples were used for metabolomics assay. Before sample preparation, the sample sequence was randomized to avoid bias. For the metabolomics sample preparation, 150 µL of an extraction solution containing internal standards made up of 5 mL water, 5 mL methanol, 10 µL debrisoquine (1 mg/mL in ddH2O), and 50 µL of 4-Nitrobenzoic acid (1 mg/mL in Methanol) (per 10 mL) was added to the fecal pellet. The samples were vortexed for 15 min then incubated on ice for 20 min. Next, 150 µL of chilled acetonitrile was added, the samples were vortexed, then were incubated at −20 °C for 20 min. Lastly, the samples were centrifuged at 15 493 x g for 20 min at 4 °C and the supernatant was transferred to a MS vial for LC-MS analysis.

A volume of 2 µL of each prepared sample was injected onto a Waters Acquity BEH C18 1.7 μm, 2.1 × 50 mm column for metabolomics using an Acquity UPLC system coupled to a Xevo G2-S quadrupole-time-of-flight mass spectrometer with an electrospray ionization source (UPLC-ESI-QToF-MS) (Waters Corporation, Milford, MA). The mobile phases consisted of 100% water (solvent A), 100% acetonitrile containing 0.1% formic acid (solvent B), and 100% isopropanol with 0.1% formic acid (solvent C).

The solvent flow rate for the metabolomics acquisition was set to 0.4 mL/min with the column set at 60 °C. The LC gradient was as follows: Initial – 95% A, 5% B; 0.5 min – 95% A, 5% B; 8.0 min – 2% A, 98% B; 9.0 min – 11.8% B, 88.2% C; 10.5 min – 11.8% B, 88.2% C; 11.5 min – 50% A, 50% B; 12.5 min – 95% A, 5% B; 13.0 min – 95% A, 5% B.

The column eluent was introduced into the Xevo G2-S mass spectrometer by electrospray operating in either negative or positive electrospray ionization mode. Positive mode had a capillary voltage of 3.00 kV and a sampling cone voltage of 30 V. Negative mode had a capillary voltage of 2.00 kV and had a sampling cone voltage of 30 V. The desolvation gas flow was set to 600 L/h and the desolvation temperature was set to 500 °C. The cone gas flow was 25 L/h and the source temperature was set to 100 °C. The data were acquired in the sensitivity MS mode with a scan time of 0.300 s and an interscan time of 0.014 s. Accurate mass was maintained by infusing Leucine Enkephalin (556.2771 [M + H] + /554.2615 [M-H]-) in 50% aqueous acetonitrile (2.0 ng/mL) at a rate of 10 µL/min via the Lockspray interface every 10 s. The data were acquired in centroid mode with a 50.0 to 1 200.0 m/z mass range for TOF-MS scanning. An aliquot of each sample was pooled and used as a quality control (QC) which represented all metabolites present. This QC sample was run at the beginning of the sequence to condition the column and then injected every 10 samples to check mass accuracy, ensure presence of internal standard, and to monitor shifts in retention time and signal intensities.

### LC-MS data processing and analysis

The untargeted data acquired were first converted to the NetCDF unified data format using the Databridge tool in MassLynx (Waters Corporation, Milford, MA). LC-MS spectral feature were annotated in Human Metabolome Database (HMDB V4.0) (https://hmdb.ca/spectra/ms/search) to curate putative metabolites. Adduct type M + H, M + 2H, M+Na, M + K, and M + NH4 were chosen for positive ion mode. M-H, M+Cl, M + FA-H, and M-H-H2O were chosen for negative ion mode. Molecular weight tolerance was set as 10 ppm. LC-MS spectral features’ abundance values were log transformed. The raw LC-MS data has been deposited in the EMBL-EBI MetaboLights database^45^ with the identifier MTBLS10321.

Unsupervised principle component analysis (PCA) and supervised sparse Partial Least Squares-Discriminant Analysis (sPLS-DA) were performed by MetaboAnalyst 5.0^46^ and visualized by R package ggplot2.^47^ The optimal complexity of the sPLS-DA model was assessed by five-fold cross-validation. The top 10 variables for component 1 of sPLS-DA model were selected for loading plot.

Pathway enrichment analysis was performed by MetaboAnalyst 5.0. Annotated LC-MS peaks were ranked by p value of t-test with 0.01 as cutoff value using Mummichog algorithm.^48^ Molecular weight tolerance was set as 10 ppm. Adduct type M + H, M + 2H, M+Na, M + K, M + NH4, M-H, M+Cl, M + FA-H, and M-H-H2O were selected. Mus musculus BioCyc and Mus musculus KEGG databases were used for pathway libraries. Enriched pathways were visualized with bubble plot by ggplot2.

### Integrated 16S microbiome and LC-MS metabolome analysis

Metabolites derived from significantly altered 16S predicted pathways (*P* < 0.05) were annotated using Metacyc (V23.5), HMDB (V4.0), and KEGG (V93.0) (Table S5). The conversion between chemical name, HMDB ID and KEGG ID was performed by online bioinformatics tool Chemical Translation Service (https://cts.fiehnlab.ucdavis.edu/batch). If there was no hit, a second search was performed in HMDB database and KEGG database, respectively (Table S6). The metabolites derived from 16S predicted pathway were then compared to the fecal metabolites from LC-MS dataset to identify common metabolites associated with LC-MS peaks (Table S7).

The integrative analysis of 16S sequencing dataset and LC-MS dataset was performed by a customized gene set enrichment analysis (GSEA) to determine whether metabolites involved in 16S predicted pathways were enriched in LC-MS metabolomics dataset.^49^ Most 16S metagenome-linked predicted pathways contained only a small number of metabolites that could be annotated with LC-MS spectral features, which might result in less power to detect significance. Thus, 16S predicted pathways were classified into a higher superclass pathways according to the Metacyc database (V23.5). The superclass pathways with their associated LC-MS spectral features were used as the customized metabolite sets. The LC-MS peak intensity dataset was used as expression dataset. The metric *t* Test (which uses the difference of means scaled by the standard deviation and number of samples) was selected for ranking. The minimum size of the metabolite set was set as 10 and default settings were used for other parameters.

### Statistical analysis

Data were represented as mean ± standard error of mean (SEM) or mean with 95% confidence intervals (95% CI) as indicated in the text. Microbial β-diversities were compared by PERMANOVA with 999 permutations using “adonis” function in R package Vegan.^50^ Unpaired *t* test (parametric data) or Mann–Whitney U test (non-parametric data) was used for two group comparisons including the microbiome α-diversities, taxa relative abundance, LC-MS peak intensities, inflammatory biomarkers, and micro-CT data. One-way ANOVA (parametric data) or Kruskal-Wallis test (non-parametric data) with Tukey post hoc was used for three group comparisons of the micro-CT data. Two-way ANOVA with Tukey post hoc was used for the micro-CT data with two independent variables (age and colonization). If the data did not meet the assumptions of two-way ANOVA, Mann–Whitney U test was used. Benjamini-Hochberg (BH) adjusting was applied for multiple comparisons. * indicates *P* < 0.05. ** indicates *P* < 0.01. *** indicates *P* < 0.001. ns indicates not statistically significant.

## Supplementary information

Supplementary figures and tables
