# Supplementary material for: Bone loss with aging is independent of gut microbiome in mice
Source: Bone Res. 2024 Nov 11;12:65. doi: 10.1038/s41413-024-00366-0 (PMC11551211; doi:10.1038/s41413-024-00366-0)
Supplement: Supplementary file 1 — Supplementary figures and tables [file 41413_2024_366_MOESM1_ESM.pdf]

Supplementary figure legends

Fig. S1 Femur bone parameters of germ-free (GF) mice and colonized (Col) mice at 24 months old.

The comparison between GF and colonized mice of trabecular number (Tb. N), trabecular thickness (Tb. Th), trabecular spacing (Tb. Sp), cortical periosteal perimeter (Ps. Pm) and cortical tissue mineral density (Ct. TMD) is shown for females (A-E) and males (F-J) at 24-month-old. Unpaired t test or Mann-Whitney U test was performed. *, $p<0.05$, **, $p<0.01$, ns, not statistically significant.

Fig. S2 GF mice were not protected from bone loss with aging.

Study design (A). Trabecular bone volume fraction (Tb. BV/TV), trabecular number (Tb. N), trabecular thickness (Tb. Th), trabecular spacing (Tb. Sp), cortical thickness (Ct. Th), cortical area (Ct. Ar), cortical periosteal perimeter (Ps. Pm), cortical tissue mineral density (Ct. TMD) are shown for females (B-I) and males (L-S). Gray-level (J) and porosity (K) of GF and SPF females are shown. Representative 3D images of femur trabecular bone are shown (T). Data are represented as mean±SEM. Statistical analysis was performed to compare bone phenotypes between 3-month-old and 24-month-old mice under GF and SPF conditions, respectively, using unpaired t test or Mann-Whitney U Test. No statistical analysis was performed to directly compare GF and SPF mice given potential confounding factors including differences in genetic backgrounds that mice were generated from different inbred colonies that would experience distinct genetic drift, and differences in housing environments. *, $p<0.05$, ***, $p<0.001$, ns, not statistically significant.

Fig. S3 Distinct fecal microbiomes between young and old mice

Principal coordinate analysis (PCoA) plots of unweighted UniFrac distance and Jaccard distance (A). Rarefaction curve is shown in (B). α-diversities based on Chao1 and Shannon indices (C). Relative abundance of taxa at genus level (D). Age-specific discriminant taxa at all level identified by LEfSe analysis (E). Venn plot of common differentially expressed metabolites of predicted metagenome of 16s dataset and metabolome dataset (F). Fecal microbiome analysis by qRT-PCR in recipient mice after 1-month (G) and 8-month colonization (H). Mann-Whitney U test or unpaired t test was performed. *, $p<0.05$.

Fig.S4 The effect of 1-month microbial colonization on bone parameters is independent of donors' age

Effect of colonization for 1 month on trabecular number (Tb. N), trabecular thickness (Tb. Th), trabecular spacing (Tb. Sp), cortical periosteal perimeter (Ps. Pm) and cortical tissue mineral density (Ct. TMD) in young adult (2-month-old, A-E) and skeletal immature (1-month-old, F-J) females is shown. One-way ANOVA or Kruskal-Wallis test with Tukey post hoc was performed. *, $p<0.05$, **, $p<0.01$, ***, $p<0.001$, ns, not statistically significant

Fig.S5 The effect of 8-month microbial colonization on bone parameters is independent of donors' age.

Effect of colonization for 8 months on trabecular number (Tb. N), trabecular thickness (Tb. Th), trabecular spacing (Tb. Sp), cortical periosteal perimeter (Ps. Pm) and cortical tissue mineral density (Ct. TMD) is shown in female (A-E) and male (F-J) mice. One-way ANOVA or Kruskal-Wallis test with Tukey post hoc was performed. ns, not statistically significant

Fig. S1

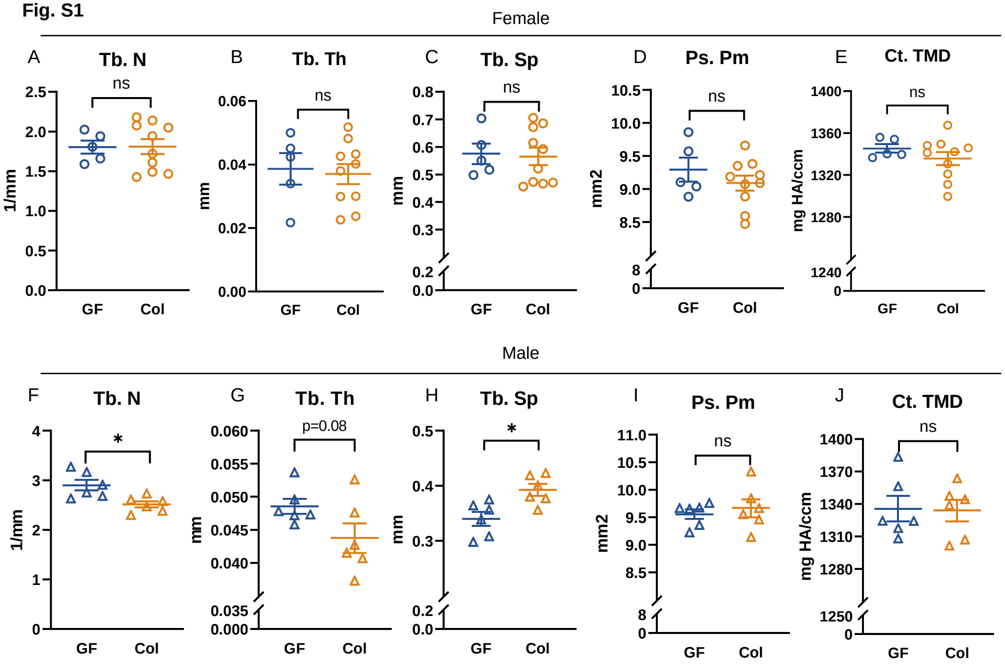

Female
A
Tb. N
ns
1/mm
2.5
2.0
1.5
1.0
0.5
0.0
GF
Col
B
Tb. Th
ns
mm
0.06
0.04
0.02
0.00
GF
Col
C
Tb. Sp
ns
mm
0.8
0.7
0.6
0.5
0.4
0.3
0.2
0.0
GF
Col
D
Ps. Pm
ns
mm2
10.5
10.0
9.5
9.0
8.5
8
0
GF
Col
E
Ct. TMD
ns
mg HA/ccm
1400
1360
1320
1280
1240
0
GF
Col
Male
F
Tb. N
*
1/mm
4
3
2
1
0
GF
Col
G
Tb. Th
p=0.08
mm
0.060
0.055
0.050
0.045
0.040
0.035
0.000
GF
Col
H
Tb. Sp
*
mm
0.5
0.4
0.3
0.2
0.0
GF
Col
I
Ps. Pm
ns
mm2
11.0
10.5
10.0
9.5
9.0
8.5
8
0
GF
Col
J
Ct. TMD
ns
mg HA/ccm
1400
1370
1340
1310
1280
1250
0
GF
Col

Fig. S2

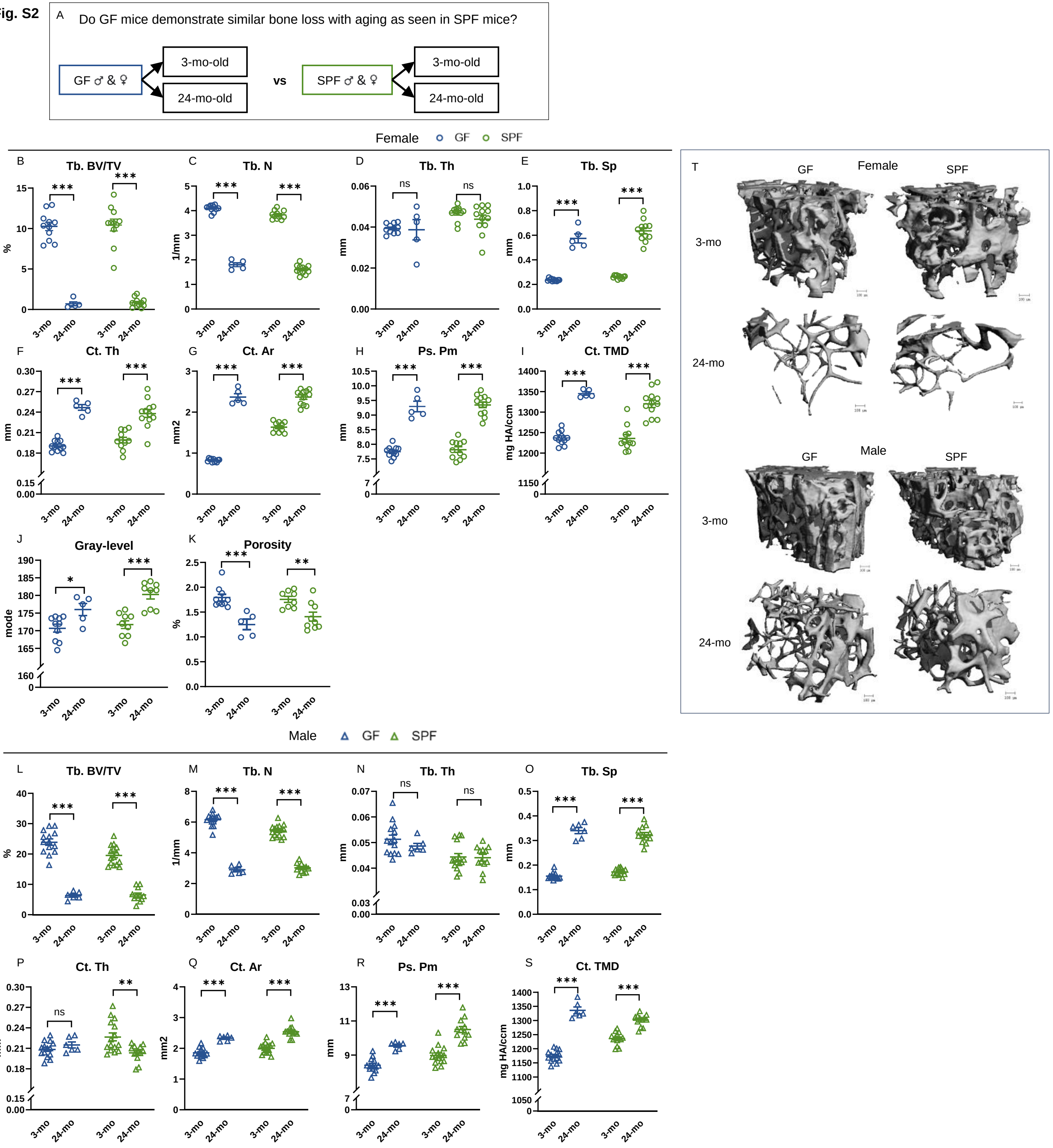
A
Do GF mice demonstrate similar bone loss with aging as seen in SPF mice?
GF ♂ & ♀
3-mo-old
24-mo-old
vs
SPF ♂ & ♀
3-mo-old
24-mo-old
Female GF SPF
B Tb. BV/TV
C Tb. N
D Tb. Th
E Tb. Sp
F Ct. Th
G Ct. Ar
H Ps. Pm
I Ct. TMD
J Gray-level
K Porosity
%
1/mm
mm
mm2
mg HA/ccm
mode
3-mo
24-mo
***
**
*
ns
T
Female
GF
SPF
3-mo
24-mo
Male
GF
SPF
3-mo
24-mo
Male GF SPF
L Tb. BV/TV
M Tb. N
N Tb. Th
O Tb. Sp
P Ct. Th
Q Ct. Ar
R Ps. Pm
S Ct. TMD

Fig. S3

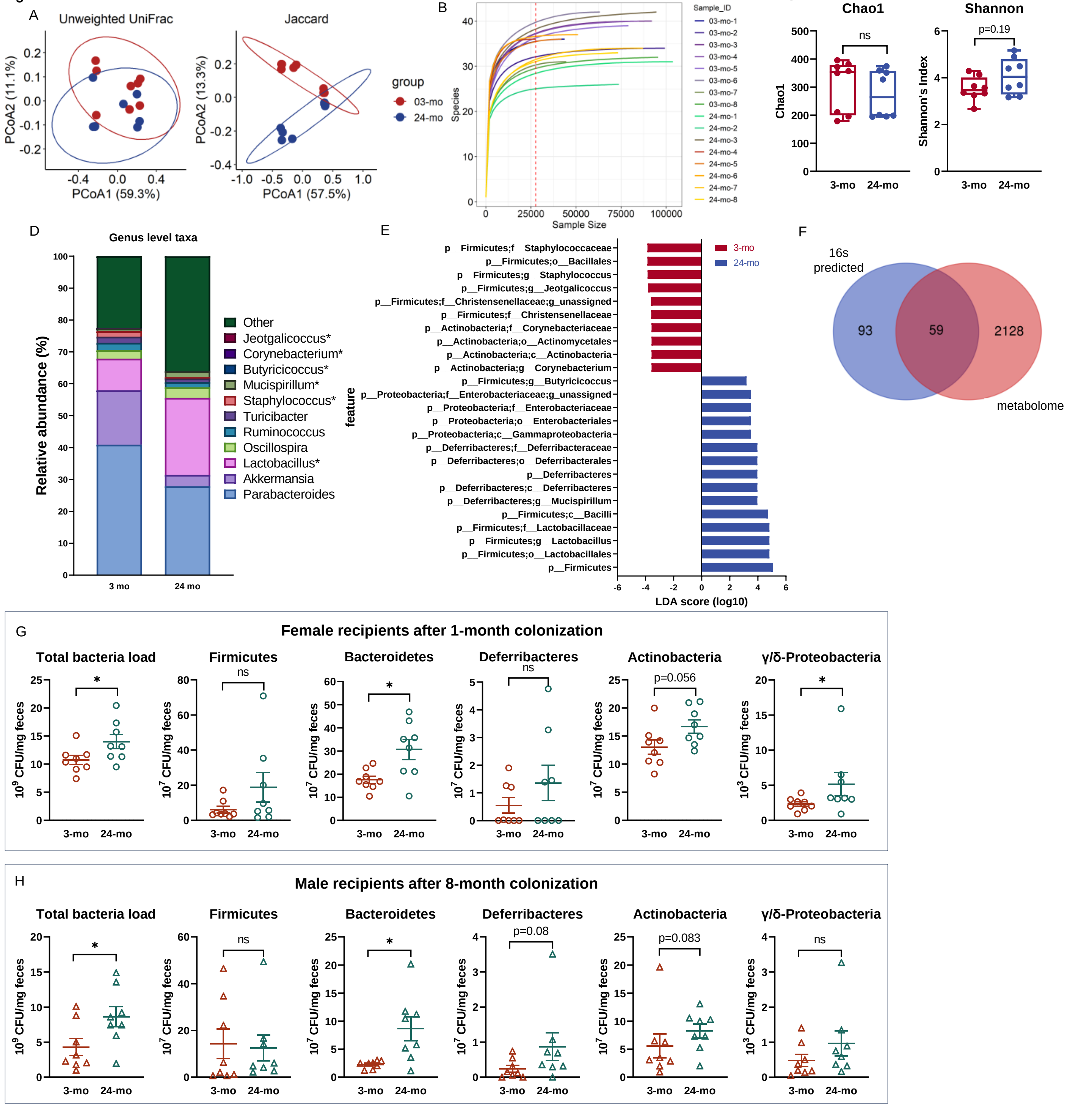

A
Unweighted UniFrac
Jaccard
PCoA1 (59.3%)
PCoA2 (11.1%)
PCoA1 (57.5%)
PCoA2 (13.3%)
group
03-mo
24-mo
B
Rarefaction Curve
Species
Sample Size
Sample_ID
C
Chao1
Shannon
ns
p=0.19
Shannon's Index
3-mo
24-mo
D
Genus level taxa
Relative abundance (%)
Other
Jeotgalicoccus*
Corynebacterium*
Butyricicoccus*
Mucispirillum*
Staphylococcus*
Turicibacter
Ruminococcus
Oscillospira
Lactobacillus*
Akkermansia
Parabacteroides
3 mo
24 mo
E
feature
LDA score (log10)
p__Firmicutes;f__Staphylococcaceae
p__Firmicutes;o__Bacillales
p__Firmicutes;g__Staphylococcus
p__Firmicutes;g__Jeotgalicoccus
p__Firmicutes;f__Christensenellaceae;g_unassigned
p__Firmicutes;f__Christensenellaceae
p__Actinobacteria;f__Corynebacteriaceae
p__Actinobacteria;o__Actinomycetales
p__Actinobacteria;c__Actinobacteria
p__Actinobacteria;g__Corynebacterium
p__Firmicutes;g__Butyricicoccus
p__Proteobacteria;f__Enterobacteriaceae;g_unassigned
p__Proteobacteria;f__Enterobacteriaceae
p__Proteobacteria;o__Enterobacteriales
p__Proteobacteria;c__Gammaproteobacteria
p__Deferribacteres;f__Deferribacteraceae
p__Deferribacteres;o__Deferribacterales
p__Deferribacteres
p__Deferribacteres;c__Deferribacteres
p__Deferribacteres;g__Mucispirillum
p__Firmicutes;c__Bacilli
p__Firmicutes;f__Lactobacillaceae
p__Firmicutes;g__Lactobacillus
p__Firmicutes;o__Lactobacillales
p__Firmicutes
F
16s predicted
metabolome
93
59
2128
G
Female recipients after 1-month colonization
Total bacteria load
Firmicutes
Bacteroidetes
Deferribacteres
Actinobacteria
γ/δ-Proteobacteria
*
ns
p=0.056
H
Male recipients after 8-month colonization
p=0.08
p=0.083

Fig. S4

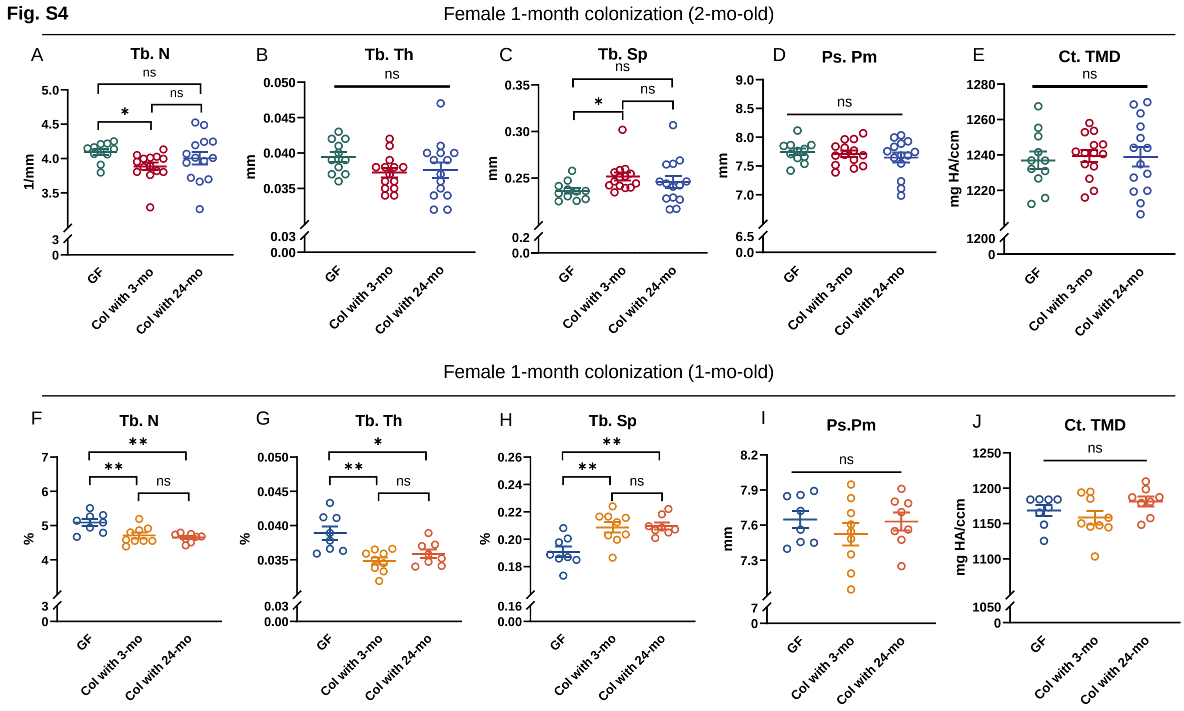

Female 1-month colonization (2-mo-old)
A Tb. N
B Tb. Th
C Tb. Sp
D Ps. Pm
E Ct. TMD
1/mm
mm
mm
mm
mg HA/ccm
ns
*
GF
Col with 3-mo
Col with 24-mo
Female 1-month colonization (1-mo-old)
F Tb. N
G Tb. Th
H Tb. Sp
I Ps.Pm
J Ct. TMD
%
%
%
mm
mg HA/ccm
**

Fig. S5

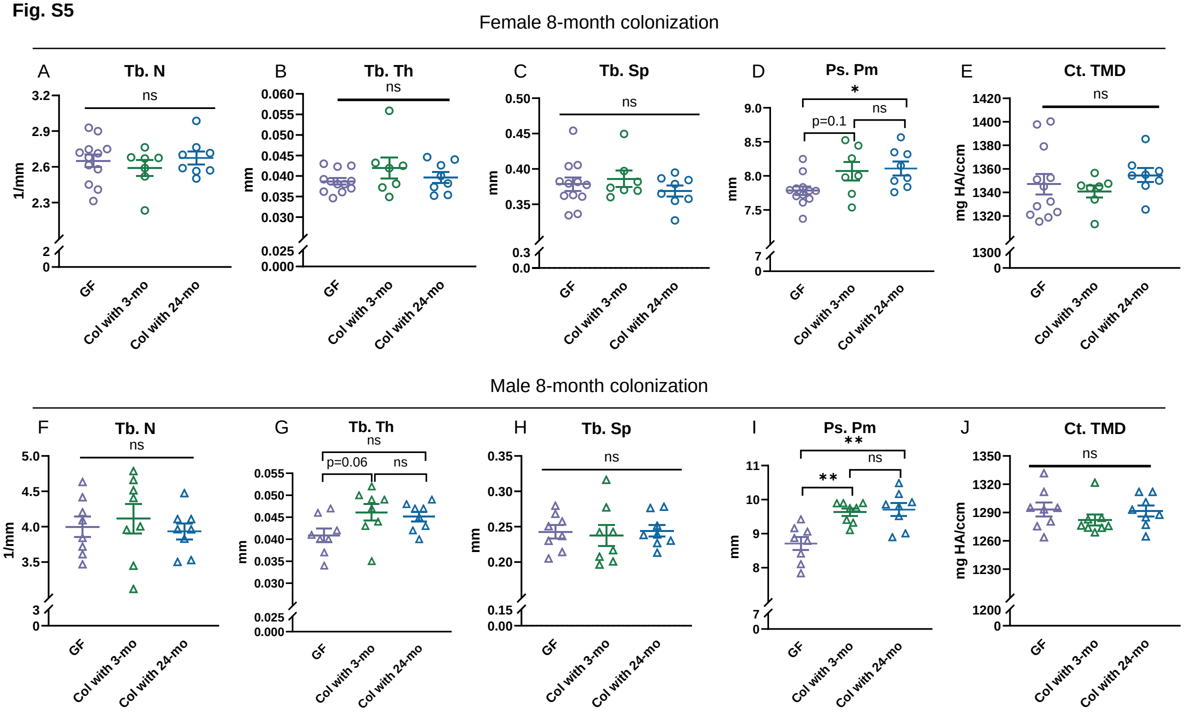

Female 8-month colonization
A Tb. N
B Tb. Th
C Tb. Sp
D Ps. Pm
E Ct. TMD
1/mm
mm
mg HA/ccm
ns
*
p=0.1
GF
Col with 3-mo
Col with 24-mo
Male 8-month colonization
F Tb. N
G Tb. Th
H Tb. Sp
I Ps. Pm
J Ct. TMD
p=0.06
**

Table S1. Relative abundance of taxa at the phylum level (mean±SEM %)

	3-mo	24-mo	p value
Bacteroidetes	40.94±4.82	27.9±4.02	0.105
Cyanobacteria	0±0	0±0	0.721
Deferribacteres	0.78±0.08	1.84±1.54	0.038
Firmicutes	40.93±3.03	66.3±4.45	0.001
Proteobacteria	0.01±0	0.02±0.01	0.442
Tenericutes	0.26±0.06	0.36±0.09	0.382
Verrucomicrobia	17.04±7.04	3.57±2.91	0.161
Unassigned	0.04±0.02	0.01±0.01	0.161

Table S2. Relative abundance of taxa at the genus level (mean±SEM %)

	3-mo	24-mo	p value
Acidaminococcus	0±0	0±0	0.959
Adlercreutzia	0.01±0	0.01±0	0.574
Aerococcus	0.01±0	0.01±0.01	0.645
Akkermansia	17.04±7.04	3.57±2.91	0.161
Anaerostipes	0.04±0.01	0.09±0.06	0.878
Bacteroides	0.01±0	0.01±0	0.442
Blautia	0±0	0±0	0.721
Butyricicoccus	0.04±0.01	0.16±0.05	0.038
Clostridium	0.07±0.01	0.12±0.04	1.000
Coprobacillus	0±0	0±0	0.382
Coprococcus	0.53±0.08	1.14±0.33	0.279
Corynebacterium	0.03±0.01	0±0	0.038
Defluviitalea	0±0	0±0	0.382
Dehalobacterium	0.05±0.01	0.04±0.01	1.000
Dorea	0.17±0.02	0.14±0.02	0.382
Enterococcus	0±0	0±0	0.234
Helicobacter	0±0	0±0	1.000
Jeotgalicoccus	0.02±0.01	0±0	0.010
Lactobacillus	9.91±1.21	24.12±3.68	0.010
Mucispirillum	0.78±0.08	1.84±1.54	0.038
Oscillospira	2.72±0.43	3.3±0.58	0.721
Parabacteroides	40.93±4.83	27.89±4.02	0.105
[Prevotella]	0±0	0±0	1.000
rc4-4	0.03±0.02	0.24±0.22	0.798
Roseburia	0±0	0±0	0.442
Staphylococcus	1.75±0.42	0.43±0.13	0.038
Turicibacter	1.92±1.13	1.12±0.74	0.878
c__Bacilli;unassigned	0±0	0±0	0.328
f__[Mogibacteriaceae];unassigned	0.03±0.01	0.04±0.01	0.574
f__Christensenellaceae;unassigned	0.03±0.01	0±0	0.000
f__Enterobacteriaceae;unassigned	0±0	0.01±0	0.105
f__Erysipelotrichaceae;unassigned	0.06±0.02	0.06±0.02	1.000
f__Lachnospiraceae;g__[Ruminococcus]	0.69±0.06	0.84±0.2	0.721
f__Lachnospiraceae;unassigned	6.54±0.83	11.19±2.2	0.161
f__mitochondria;unassigned	0.01±0	0.01±0	0.878
f__Peptococcaceae;unassigned	0.01±0	0.02±0.01	0.721
f__Peptostreptococcaceae;unassigned	0±0	0±0	0.878
f__Ruminococcaceae;g__Ruminococcus	1.56±0.42	0.8±0.07	0.234
f__Ruminococcaceae;unassigned	0.61±0.18	1.04±0.14	0.083
f__Streptococcaceae;unassigned	0±0	0.01±0	0.645
o__Clostridiales;unassigned	14.13±1.65	21.37±4.4	0.234
o__Lactobacillales;unassigned	0±0	0±0	0.328
o__RF39;unassigned	0.26±0.06	0.36±0.09	0.382
o__Streptophyta;unassigned	0±0	0±0	0.721
Unassigned	0.01±0	0.01±0.01	0.161

Table S3. Differentially expressed 16S predicted metagenomic pathways (mean±SEM TMM)

superclass	pathway	description	3-mo	24-mo	p value
Amine and Polyamine Biosynthesis	POLYAMINSYN3-PWY	superpathway of polyamine biosynthesis II	547.93±42.49	739.53±72.09	0.042
Amine and Polyamine Biosynthesis	POLYAMSYN-PWY	superpathway of polyamine biosynthesis I	509.73±40.22	701.65±74.94	0.046
Amine and Polyamine Biosynthesis	ARG+POLYAMINE-SYN	superpathway of arginine and polyamine biosynthesis	914.91±64.66	1220.23±121.45	0.049
Amine and Polyamine Degradation	PWY0-41	allantoin degradation IV (anaerobic)	64.27±11.77	28.46±3.27	0.019
Amine and Polyamine Degradation	GLCMANNANAUT-PWY	superpathway of N-acetylglucosamine, N-acetylmannosamine and N-acetylneuraminate degradation	5878.6±161.2	8771.52±270.13	0.000
Amino Acid Biosynthesis	PWY-6151	S-adenosyl-L-methionine cycle I	2980.73±93.52	5028.79±278.35	0.000
Amino Acid Biosynthesis	PWY-2942	L-lysine biosynthesis III	8104.79±68.49	8727.34±124.48	0.001
Amino Acid Biosynthesis	PWY-2941	L-lysine biosynthesis II	2867.57±206.84	4721.62±410.48	0.002
Amino Acid Biosynthesis	PWY-5097	L-lysine biosynthesis VI	7966.61±65.83	8682.32±161.02	0.002
Amino Acid Biosynthesis	DAPLYSINESYN-PWY	L-lysine biosynthesis I	4103.15±190.8	5181.79±357.95	0.023
Amino Acid Biosynthesis	PWY-5154	L-arginine biosynthesis III (via N-acetyl-L-citrulline)	4466±138.11	3649.85±275.23	0.024
Amino Acid Biosynthesis	PWY-5345	superpathway of L-methionine biosynthesis (by sulfhydrylation)	2042.71±596.07	502.12±286.16	0.042
Amino Acid Degradation	HISDEG-PWY	L-histidine degradation I	3595.88±291.12	2478.19±301.99	0.019
Aminoacyl-tRNA Charging	TRNA-CHARGING-PWY	tRNA charging	7559.19±45.97	8007.08±136.54	0.013
Aromatic Compound Degradation	PWY-5180	toluene degradation I (aerobic) (via o-cresol)	86.2±26.04	0±0	0.013
Aromatic Compound Degradation	PWY-5182	toluene degradation II (aerobic) (via 4-methylcatechol)	86.2±26.04	0±0	0.013
C1 Compound Utilization and Assimilation	P42-PWY	incomplete reductive TCA cycle	9887.75±233.41	7362.77±534.04	0.002
C1 Compound Utilization and Assimilation	RUMP-PWY	formaldehyde oxidation I	906.54±81.57	659.11±76.77	0.044
Carbohydrate Biosynthesis	PWY-6478	GDP-D-glycero-α-D-manno-heptose biosynthesis	5065.75±126.48	3649.59±204.62	0.000
Carbohydrate Biosynthesis	UDPNAGSYN-PWY	UDP-N-acetyl-D-glucosamine biosynthesis I	3216.19±111.44	5206.46±282.92	0.000
Carbohydrate Biosynthesis	OANTIGEN-PWY	O-antigen building blocks biosynthesis (E. coli)	3966.93±134.33	5653.13±248.37	0.000
Carbohydrate Biosynthesis	PWY-5659	GDP-mannose biosynthesis	6559.1±137.89	5188.69±240.67	0.000
Carbohydrate Biosynthesis	GLYCOGENSYNTH-PWY	glycogen biosynthesis I (from ADP-D-Glucose)	6954.36±162.37	8685.39±318.59	0.001
Carbohydrate Biosynthesis	PWY-7332	superpathway of UDP-N-acetylglucosamine-derived O-antigen building blocks biosynthesis	3334.62±78.47	4334.93±188.99	0.001
Carbohydrate Biosynthesis	PWY-1269	CMP-3-deoxy-D-manno-octulosonate biosynthesis I	4596.85±144.39	2902.18±321.35	0.001
Carbohydrate Biosynthesis	PWY-7323	superpathway of GDP-mannose-derived O-antigen building blocks biosynthesis	4964.94±192.48	3760.29±261.89	0.003
Carbohydrate Biosynthesis	GLUCONEO-PWY	gluconeogenesis I	7635.12±99.12	8071.24±80.07	0.004
Carbohydrate Degradation	PWY-6317	galactose degradation I (Leloir pathway)	6549.58±138.2	8531.88±217.01	0.000
Carbohydrate Degradation	PWY-621	sucrose degradation III (sucrose invertase)	6664.25±401.83	9988.1±296.9	0.000
Carbohydrate Degradation	P124-PWY	Bifidobacterium shunt	3240.07±266.26	5773.71±513.84	0.001
Carbohydrate Degradation	ANAGLYCOLYSIS-PWY	glycolysis III (from glucose)	9417.42±48.2	9861.95±97.05	0.002
Carbohydrate Degradation	LACTOSECAT-PWY	lactose and galactose degradation I	1060.74±88.87	2379.03±332.07	0.005
Carbohydrate Degradation	PWY-6737	starch degradation V	9303.94±177.24	8438.69±260.64	0.017
Carbohydrate Degradation	RHAMCAT-PWY	L-rhamnose degradation I	4323.71±311.32	3248.4±310.98	0.028
Cell Wall Biosynthesis	TEICHOICACID-PWY	teichoic acid (poly-glycerol) biosynthesis	1654.54±131.63	2817.26±331.07	0.010
Cell Wall Biosynthesis	PWY-6470	peptidoglycan biosynthesis V (β-lactam resistance)	520.46±147.11	127.91±40.47	0.033
Cell Wall Biosynthesis	PWY-5265	peptidoglycan biosynthesis II (staphylococci)	563.04±166.12	137.49±42.48	0.038
Cell Wall Biosynthesis	PWY-6471	peptidoglycan biosynthesis IV (Enterococcus faecium)	3456.95±200.46	5568.04±272.54	0.000
Cell Wall Biosynthesis	PWY-6387	UDP-N-acetylmuramoyl-pentapeptide biosynthesis I (meso-diaminopimelate containing)	7288.73±118.48	8320.06±127.7	0.000
Cell Wall Biosynthesis	PEPTIDOGLYCANSYN-PWY	peptidoglycan biosynthesis I (meso-diaminopimelate containing)	7258.89±99.67	8199.45±121.42	0.000
Cell Wall Biosynthesis	PWY-6385	peptidoglycan biosynthesis III (mycobacteria)	7177.41±126.48	8174.36±126.89	0.000
Cell Wall Biosynthesis	PWY-6386	UDP-N-acetylmuramoyl-pentapeptide biosynthesis II (lysine-containing)	7109.31±174.67	8175.58±154.95	0.000
Cell Wall Biosynthesis	PWY0-1586	peptidoglycan maturation (meso-diaminopimelate containing)	3869.37±252.68	7028.88±560.46	0.000
Energy	TCA	TCA cycle I (prokaryotic)	7625.47±124.41	5505.51±343.82	0.000
Energy	PWY-6969	TCA cycle V (2-oxoglutarate:ferredoxin oxidoreductase)	8053.99±150.78	5872.43±361.04	0.000
Energy	PWY-5913	TCA cycle VI (obligate autotrophs)	7413.9±101.86	5599.09±377.18	0.002
Energy	PWY-7254	TCA cycle VII (acetate-producers)	390.63±122.04	78.11±24.12	0.038
Fermentation	FERMENTATION-PWY	mixed acid fermentation	4364.42±96.88	5908.16±228.44	0.000
Fermentation	P108-PWY	pyruvate fermentation to propanoate I	6993.48±165.57	4927.9±362.65	0.000
Fermentation	P161-PWY	acetylene degradation	2969.23±83.56	5055.81±351.07	0.000
Fermentation	P122-PWY	heterolactic fermentation	2585.04±203.61	4321.02±381.93	0.002
Fermentation	ANAEROFRUCAT-PWY	homolactic fermentation	7345.71±126.89	8226.48±207.64	0.004
Fermentation	PWY-5100	pyruvate fermentation to acetate and lactate II	8044.99±183.99	9175.87±423	0.035
Fatty Acid and Lipid Biosynthesis	PWY4FS-7	phosphatidylglycerol biosynthesis I (plastidic)	5725.79±237.89	7471.01±347.37	0.001
Fatty Acid and Lipid Biosynthesis	PWY4FS-8	phosphatidylglycerol biosynthesis II (non-plastidic)	5725.79±237.89	7471.01±347.37	0.001
Fatty Acid and Lipid Biosynthesis	PWY-5667	CDP-diacylglycerol biosynthesis I	8131.03±64.2	9243.7±240.9	0.002

Fatty Acid and Lipid Biosynthesis	PWY0-1319	CDP-diacylglycerol biosynthesis II	8131.03±64.2	9243.7±240.9	0.002
Fatty Acid and Lipid Biosynthesis	PHOSLIPSYN-PWY	superpathway of phospholipid biosynthesis I (bacteria)	7050.48±75.15	8115.08±299.67	0.009
Fatty Acid and Lipid Biosynthesis	PWY-5973	cis-vaccenate biosynthesis	8666.58±63.91	9234.93±100.92	0.000
Fatty Acid and Lipid Biosynthesis	PWY-7663	gondoate biosynthesis (anaerobic)	9153.64±77.23	9704.96±100.02	0.001
Inorganic Nutrient Metabolism	PWY-5304	superpathway of sulfur oxidation (Acidianus ambivalens)	10387.66±794.37	7011.3±883.3	0.013
Inorganic Nutrient Metabolism	SULFATE-CYS-PWY	superpathway of sulfate assimilation and cysteine biosynthesis	2773.11±749.97	767.83±426.78	0.040
Lipopolysaccharide Biosynthesis	NAGLIPASYN-PWY	lipid IVA biosynthesis	4453.35±142.39	2799.25±313.74	0.001
Lipopolysaccharide Biosynthesis	PWY-6467	Kdo transfer to lipid IVA III (Chlamydia)	4046.51±129.56	2530.16±283.27	0.001
NAD Metabolism	PYRIDNUCSYN-PWY	NAD biosynthesis I (from aspartate)	5871.84±81.12	4774.15±133.31	0.000
Nucleic Acid Processing	PWY-6700	queuosine biosynthesis	5889.83±139.53	4330.21±269.55	0.000
Nucleoside and Nucleotide Biosynthesis	PWY-7199	pyrimidine deoxyribonucleosides salvage	3115.92±79.78	4223.86±102.87	0.000
Nucleoside and Nucleotide Biosynthesis	PWY-6121	5-aminoimidazole ribonucleotide biosynthesis I	8874.47±76.31	9499.08±66.85	0.000
Nucleoside and Nucleotide Biosynthesis	PWY-6122	5-aminoimidazole ribonucleotide biosynthesis II	9459.26±95.78	10078.08±67.52	0.000
Nucleoside and Nucleotide Biosynthesis	PWY-6277	superpathway of 5-aminoimidazole ribonucleotide biosynthesis	9459.26±95.78	10078.08±67.52	0.000
Nucleoside and Nucleotide Biosynthesis	PWY-7219	adenosine ribonucleotides de novo biosynthesis	8534.07±48	9259.91±141.12	0.001
Nucleoside and Nucleotide Biosynthesis	PWY-6123	inosine-5'-phosphate biosynthesis I	7996.29±74.66	8378.86±56.15	0.001
Nucleoside and Nucleotide Biosynthesis	PWY-6609	adenine and adenosine salvage III	6971.65±156.51	7787.14±148.73	0.002
Nucleoside and Nucleotide Biosynthesis	PWY-7234	inosine-5'-phosphate biosynthesis III	3872.29±128.64	5946.99±506.48	0.004
Nucleoside and Nucleotide Biosynthesis	PWY-7200	superpathway of pyrimidine deoxyribonucleoside salvage	3471.49±92.96	4498.78±253.73	0.004
Nucleoside and Nucleotide Biosynthesis	PWY-5686	UMP biosynthesis	8489.78±39.13	9160.62±167.22	0.005
Nucleoside and Nucleotide Biosynthesis	PWY-7208	superpathway of pyrimidine nucleobases salvage	9360.03±173.77	9979.39±162.31	0.021
Biosynthesis	PWY-7229	novo biosynthesis I	8941.64±115.66	9308.74±124.8	0.049
Nucleoside and Nucleotide Degradation	PWY0-1298	superpathway of pyrimidine deoxyribonucleosides degradation	3298.02±42.88	5089.18±204.7	0.000
Nucleoside and Nucleotide Degradation	PWY0-1296	purine ribonucleosides degradation	4490.58±101.91	6267.99±240.55	0.000
Nucleoside and Nucleotide Degradation	PWY0-1297	superpathway of purine deoxyribonucleosides degradation	4456.28±109.16	5784.04±243.86	0.001
Nucleoside and Nucleotide Degradation	PWY-5695	urate biosynthesis/inosine 5'-phosphate degradation	5701.39±173.05	4108.75±349.01	0.002
Nucleoside and Nucleotide Degradation	PWY-6608	guanosine nucleotides degradation III	4355.85±205.15	3245.94±287.17	0.008
Polyprenyl Biosynthesis	POLYISOPRENSYN-PWY	polyisoprenoid biosynthesis (E. coli)	5984.04±81.63	5120.49±291.71	0.021
Porphyrin Compound Biosynthesis	HEMESYN2-PWY	heme biosynthesis II (anaerobic)	1763.63±138.75	3558.46±353.63	0.001
Porphyrin Compound Biosynthesis	HEME-BIOSYNTHESIS-II	heme biosynthesis I (aerobic)	1616.56±126.68	2691.16±256.09	0.004
Quinol and Quinone Biosynthesis	PWY-5838	superpathway of menaquinol-8 biosynthesis I	2141.22±603.82	582.59±366.62	0.048
Quinol and Quinone Biosynthesis	PWY-5840	superpathway of menaquinol-7 biosynthesis	2116.07±592.58	586.72±369.6	0.050
Secondary Metabolite Biosynthesis	PWY-6703	preQ0 biosynthesis	5818.87±128.37	3987.19±253.62	0.000
Secondary Metabolite Biosynthesis	PWY-5910	superpathway of geranylgeranyldiphosphate biosynthesis I (via mevalonate)	1333.09±101.02	2525.95±274.57	0.003
Secondary Metabolite Biosynthesis	PWY-922	mevalonate pathway I	991.02±78.78	1980.89±233.46	0.003
Secondary Metabolite Biosynthesis	PWY-7237	myo-, chiro- and scillo-inositol degradation	246.52±41.07	134.97±15.55	0.032
Secondary Metabolite Biosynthesis	PWY-7392	taxadiene biosynthesis (engineered)	3238.89±172.51	4530.47±246.03	0.001
Secondary Metabolite Degradation	P562-PWY	myo-inositol degradation I	151.73±26.67	81.63±10.89	0.037
Vitamin Biosynthesis	PWY-6897	thiamin salvage II	5441.88±125.16	4028.94±170.21	0.000
Vitamin Biosynthesis	PANTO-PWY	phosphopantothenate biosynthesis I	6234.04±119.29	4677.05±211.38	0.000
Vitamin Biosynthesis	RIBOSYN2-PWY	flavin biosynthesis I (bacteria and plants)	5905.38±66.7	5080.37±121.36	0.000
Vitamin Biosynthesis	PWY-6612	superpathway of tetrahydrofolate biosynthesis	6161.29±49.7	5213.07±145.71	0.000
Vitamin Biosynthesis	PANTOSYN-PWY	pantothenate and coenzyme A biosynthesis I	6720.6±100.87	5478.56±198.89	0.000
Vitamin Biosynthesis	FOLSYN-PWY	superpathway of tetrahydrofolate biosynthesis and salvage	6453.31±39.82	5838.74±108.97	0.001
Vitamin Biosynthesis	PWY-7539	6-hydroxymethyl-dihydropterin diphosphate biosynthesis III (Chlamydia)	5302.82±188.04	4414.33±131.09	0.002
Vitamin Biosynthesis	1CMET2-PWY	N10-formyl-tetrahydrofolate biosynthesis	7558.72±58.8	7815.74±63.47	0.010
Vitamin Biosynthesis	THISYN-PWY	superpathway of thiamin diphosphate biosynthesis I	3442.08±344.47	2371.24±165.38	0.019
Vitamin Biosynthesis	PWY-6147	6-hydroxymethyl-dihydropterin diphosphate biosynthesis I	6068.8±269.97	5197.37±173.6	0.019

Table S4. Top 10 metabolic peaks for comp1 of sPLS-DA with annotations

ion mode	m.z	rtmed	abs (comp1)	hmdb_id	compound_name	kegg_id	formula	monoisotopic_mass	adduct	adduct_m.z	delta.ppm.
pos	1192.8317	9.76	0.14298	HMDB0004894	Ganglioside GA2 (d18:1/24:1(15Z))	C06135	C62H114N2O18	1174.806665	M+NH4	1192.840488	7
neg	142.049	0.4	0.14546	HMDB0000510	Aminoadipic acid	C00956	C6H11NO4	161.0688078	M-H20-H	142.050418	10
		0.4	0.14546	HMDB0062557	N-Acetylthreonine	n/a	C6H11NO4	161.0688078	M-H20-H	142.050418	10
		0.4	0.14546	HMDB0029423	O-acetyl-L-homoserine	n/a	C6H11NO4	161.0688078	M-H20-H	142.050418	10
		0.4	0.14546	HMDB0062660	N-methyl-L-glutamic Acid	C01046	C6H11NO4	161.0688078	M-H20-H	142.050418	10
		0.4	0.14546	HMDB0061715	L-Glutamic acid 5-methyl ester	n/a	C6H11NO4	161.0688078	M-H20-H	142.050418	10
		0.4	0.14546	HMDB0094716	hydroxybutyrylglycine	n/a	C6H11NO4	161.0688078	M-H20-H	142.050418	10
		0.4	0.14546	HMDB0033747	(±)-2,2'-Iminobispropanoic acid	C03210	C6H11NO4	161.0688078	M-H20-H	142.050418	10
pos	611.3312	3.44	0.17228	HMDB0010344	Vitamin D2 3-glucuronide	n/a	C34H52O7	572.371304	M+K	611.334462	5
		3.44	0.17228	HMDB0033078	Ganoderic acid W	n/a	C34H52O7	572.371304	M+K	611.334462	5
		3.44	0.17228	HMDB0035329	Ganoderic acid Ma	n/a	C34H52O7	572.371304	M+K	611.334462	5
		3.44	0.17228	HMDB0036842	Hydratopyrrhoxanthinol	n/a	C37H48O6	588.3450893	M+Na	611.334307	5
neg	791.385	5.92	0.25483	HMDB0040771	Betavulgaroside II	n/a	C41H60O15	792.3932213	M-H	791.385945	1
		5.92	0.25483	HMDB0033427	Betavulgaroside VII	n/a	C41H62O16	810.4037859	M-H20-H	791.385396	1
neg	360.1921	5.2	0.26043	HMDB0040383	(±)-Pandamarine	n/a	C18H25N3O2	315.1946771	M+FA-H	360.192878	2
		5.2	0.26043	HMDB0005049	10-Nitrolinoleic acid	C13800	C18H31NO4	325.2253085	M+Cl	360.19471	7
		5.2	0.26043	HMDB0033503	Small bacteriocin	n/a	C18H31NO4	325.2253085	M+Cl	360.19471	7
		5.2	0.26043	HMDB0041248	N-[[3-Hydroxy-2-(2-pentenyl)cyclopentyl]acetyl]isoleucine	n/a	C18H31NO4	325.2253085	M+Cl	360.19471	7
pos	442.27	2.7	0.26441	HMDB0062339	N-palmitoyl phenylalanine	n/a	C25H41NO3	403.3086442	M+K	442.271802	4
neg	326.0991	0.43	0.32821	HMDB0031796	Isofenphos	C11002	C15H24NO4PS	345.1163655	M-H20-H	326.097975	3
		0.43	0.32821	HMDB0040693	N2-(gamma-Glutamyl)-4-carboxyphenylhydrazine	n/a	C12H15N3O5	281.1011706	M+FA-H	326.099372	1
		0.43	0.32821	HMDB0006867	S-(3-Methylbutanoyl)-dihydrolipoamide-E	C05119	C13H25NO2S2	291.1326704	M+Cl	326.102072	9
		0.43	0.32821	HMDB0006869	S-(2-Methylbutanoyl)-dihydrolipoamide	C05118	C13H25NO2S2	291.1326704	M+Cl	326.102072	9
neg	501.3583	7.7	0.36576	HMDB0037778	Ganoderiol I	n/a	C31H50O5	502.3658247	M-H	501.358549	0
		7.7	0.36576	HMDB0035119	Isomangiferolic acid	n/a	C30H48O3	456.3603454	M+FA-H	501.358546	0
		7.7	0.36576	HMDB0034964	alpha-Elemolic acid	C08623	C30H48O3	456.3603454	M+FA-H	501.358546	0
		7.7	0.36576	HMDB0040456	Ganoderic acid Z	n/a	C30H48O3	456.3603454	M+FA-H	501.358546	0
		7.7	0.36576	HMDB0034962	epsilon-Bulgarene	n/a	C30H48O3	456.3603454	M+FA-H	501.358546	0
		7.7	0.36576	HMDB0035263	Avenestergenin A1	n/a	C30H48O3	456.3603454	M+FA-H	501.358546	0
		7.7	0.36576	HMDB0040652	3-Hydroxy-28,13-lupanolide	n/a	C30H48O3	456.3603454	M+FA-H	501.358546	0
		7.7	0.36576	HMDB0035775	11-Deoxoglycyrrhetinic acid	n/a	C30H48O3	456.3603454	M+FA-H	501.358546	0
		7.7	0.36576	HMDB0038388	Cyclopassifloic acid B	n/a	C31H52O6	520.3763894	M-H20-H	501.357999	1
		7.7	0.36576	HMDB0034652	Soyasapogenol E	C17420	C30H48O3	456.3603454	M+FA-H	501.358546	0
		7.7	0.36576	HMDB0034655	3-Epikatonic acid	n/a	C30H48O3	456.3603454	M+FA-H	501.358546	0
		7.7	0.36576	HMDB0039690	Glycyrrhetol	n/a	C30H48O3	456.3603454	M+FA-H	501.358546	0
		7.7	0.36576	HMDB0040976	3-Epimasticadienolic acid	n/a	C30H48O3	456.3603454	M+FA-H	501.358546	0
		7.7	0.36576	HMDB0033020	Ganodermanondiol	n/a	C30H48O3	456.3603454	M+FA-H	501.358546	0
		7.7	0.36576	HMDB0036675	3beta-Hydroxy-28,13-ursanolide	n/a	C30H48O3	456.3603454	M+FA-H	501.358546	0
		7.7	0.36576	HMDB0040455	Gibberellin A37 glucosyl ester	n/a	C30H48O3	456.3603454	M+FA-H	501.358546	0
		7.7	0.36576	HMDB0031882	Sandosapogenol	n/a	C30H48O3	456.3603454	M+FA-H	501.358546	0
		7.7	0.36576	HMDB0035315	Cucurbitaxanthin A	n/a	C30H48O3	456.3603454	M+FA-H	501.358546	0
		7.7	0.36576	HMDB0034961	beta-Elemolic acid	n/a	C30H48O3	456.3603454	M+FA-H	501.358546	0
		7.7	0.36576	HMDB0034654	Katonic acid	n/a	C30H48O3	456.3603454	M+FA-H	501.358546	0
		7.7	0.36576	HMDB0036962	3-Epioleanolic acid	n/a	C30H48O3	456.3603454	M+FA-H	501.358546	0
		7.7	0.36576	HMDB0038177	3-Hydroxycycloart-24-en-21-oic acid	n/a	C30H48O3	456.3603454	M+FA-H	501.358546	0
		7.7	0.36576	HMDB0035511	Trametenolic acid B	n/a	C30H48O3	456.3603454	M+FA-H	501.358546	0

		7.7	0.36576	HMDB0002364	Oleanolic acid	C17148	C30H48O3	456.3603454	M+FA-H	501.358546	0
		7.7	0.36576	HMDB0038703	Carissic acid	n/a	C30H48O3	456.3603454	M+FA-H	501.358546	0
		7.7	0.36576	HMDB0036757	Bryonolic acid	n/a	C30H48O3	456.3603454	M+FA-H	501.358546	0
		7.7	0.36576	HMDB0032256	(+-)-Ethyl 3-hydroxy-2-methylbutyrate	n/a	C30H48O3	456.3603454	M+FA-H	501.358546	0
		7.7	0.36576	HMDB0035353	Epoxyganoderiol C	n/a	C30H48O3	456.3603454	M+FA-H	501.358546	0
pos	464.2518	2.69	0.43196	HMDB0039851	Quinoside D	n/a	C47H74O18	926.4875156	M+2H	464.251034	2
		2.69	0.43196	HMDB0040856	Tarasaponin I	n/a	C47H74O18	926.4875156	M+2H	464.251034	2
		2.69	0.43196	HMDB0030976	Tarasaponin II	n/a	C47H74O18	926.4875156	M+2H	464.251034	2
		2.69	0.43196	HMDB0034535	Araloside A	n/a	C47H74O18	926.4875156	M+2H	464.251034	2
		2.69	0.43196	HMDB0030977	Durupcoside A	n/a	C47H74O18	926.4875156	M+2H	464.251034	2
		2.69	0.43196	HMDB0039419	Cynarasaponin H	n/a	C47H74O18	926.4875156	M+2H	464.251034	2
neg	455.3525	7.7	0.54584	HMDB0034505	Soyasapogenol A	C17419	C30H50O4	474.3709101	M-H20-H	455.35252	0
		7.7	0.54584	HMDB0039692	20,24-Epoxy-25,26-dihydroxydammaran-3-one	n/a	C30H50O4	474.3709101	M-H20-H	455.35252	0
		7.7	0.54584	HMDB0035326	Ganoderiol A	n/a	C30H50O4	474.3709101	M-H20-H	455.35252	0
		7.7	0.54584	HMDB0034528	Camelliagenin A	n/a	C30H50O4	474.3709101	M-H20-H	455.35252	0
		7.7	0.54584	HMDB0034644	Priverogenin B	n/a	C30H50O4	474.3709101	M-H20-H	455.35252	0
		7.7	0.54584	HMDB0034683	(3alphaOH,20S,24S)-3,19:20,24-Diepoxydammarane-3,2	n/a	C30H50O4	474.3709101	M-H20-H	455.35252	0
		7.7	0.54584	HMDB0034961	beta-Elemolic acid	n/a	C30H48O3	456.3603454	M-H	455.353069	1
		7.7	0.54584	HMDB0031882	Sandosapogenol	n/a	C30H48O3	456.3603454	M-H	455.353069	1
		7.7	0.54584	HMDB0038703	Carissic acid	n/a	C30H48O3	456.3603454	M-H	455.353069	1
		7.7	0.54584	HMDB0040652	3-Hydroxy-28,13-lupanolide	n/a	C30H48O3	456.3603454	M-H	455.353069	1
		7.7	0.54584	HMDB0039690	Glycyrrhetol	n/a	C30H48O3	456.3603454	M-H	455.353069	1
		7.7	0.54584	HMDB0035263	Avenestergenin A1	n/a	C30H48O3	456.3603454	M-H	455.353069	1
		7.7	0.54584	HMDB0035119	Isomangiferolic acid	n/a	C30H48O3	456.3603454	M-H	455.353069	1
		7.7	0.54584	HMDB0034962	epsilon-Bulgarene	n/a	C30H48O3	456.3603454	M-H	455.353069	1
		7.7	0.54584	HMDB0034652	Soyasapogenol E	C17420	C30H48O3	456.3603454	M-H	455.353069	1
		7.7	0.54584	HMDB0035775	11-Deoxoglycyrrhetinic acid	n/a	C30H48O3	456.3603454	M-H	455.353069	1
		7.7	0.54584	HMDB0038177	3-Hydroxycycloart-24-en-21-oic acid	n/a	C30H48O3	456.3603454	M-H	455.353069	1
		7.7	0.54584	HMDB0002364	Oleanolic acid	C17148	C30H48O3	456.3603454	M-H	455.353069	1
		7.7	0.54584	HMDB0001023	4,4-Dimethylcholesta-8,14,24-trienol	C11455	C29H46O	410.3548661	M+FA-H	455.353067	1
		7.7	0.54584	HMDB0033825	Corbisterol	n/a	C29H46O	410.3548661	M+FA-H	455.353067	1
		7.7	0.54584	HMDB0032882	28-Norcyclomusalenone	n/a	C29H46O	410.3548661	M+FA-H	455.353067	1
		7.7	0.54584	HMDB0034196	(3beta,5alpha,22E,24S)-Stigmasta-7,22,25-trien-3-ol	n/a	C29H46O	410.3548661	M+FA-H	455.353067	1
		7.7	0.54584	HMDB0034964	alpha-Elemolic acid	C08623	C30H48O3	456.3603454	M-H	455.353069	1
		7.7	0.54584	HMDB0036962	3-Epioleanolic acid	n/a	C30H48O3	456.3603454	M-H	455.353069	1
		7.7	0.54584	HMDB0035511	Trametenolic acid B	n/a	C30H48O3	456.3603454	M-H	455.353069	1
		7.7	0.54584	HMDB0040455	Gibberellin A37 glucosyl ester	n/a	C30H48O3	456.3603454	M-H	455.353069	1
		7.7	0.54584	HMDB0036675	3beta-Hydroxy-28,13-ursanolide	n/a	C30H48O3	456.3603454	M-H	455.353069	1
		7.7	0.54584	HMDB0033020	Ganodermanondiol	n/a	C30H48O3	456.3603454	M-H	455.353069	1
		7.7	0.54584	HMDB0040976	3-Epimasticadienolic acid	n/a	C30H48O3	456.3603454	M-H	455.353069	1
		7.7	0.54584	HMDB0032256	(+-)-Ethyl 3-hydroxy-2-methylbutyrate	n/a	C30H48O3	456.3603454	M-H	455.353069	1
		7.7	0.54584	HMDB0033810	Mytilitol	n/a	C29H46O	410.3548661	M+FA-H	455.353067	1
		7.7	0.54584	HMDB0040456	Ganoderic acid Z	n/a	C30H48O3	456.3603454	M-H	455.353069	1
		7.7	0.54584	HMDB0034655	3-Epikatonic acid	n/a	C30H48O3	456.3603454	M-H	455.353069	1
		7.7	0.54584	HMDB0006852	5-Dehydroavenasterol	C15783	C29H46O	410.3548661	M+FA-H	455.353067	1
		7.7	0.54584	HMDB0036312	(3beta,22E,24R)-23-Methylergosta-5,7,22-trien-3-ol	n/a	C29H46O	410.3548661	M+FA-H	455.353067	1
		7.7	0.54584	HMDB0006928	Delta 8,14 -Sterol	C11508	C29H46O	410.3548661	M+FA-H	455.353067	1
		7.7	0.54584	HMDB0039196	Stigmasta-4,6-dien-3-one	n/a	C29H46O	410.3548661	M+FA-H	455.353067	1
		7.7	0.54584	HMDB0034078	22-Dehydroclerosterol	n/a	C29H46O	410.3548661	M+FA-H	455.353067	1

		7.7	0.54584	HMDB0034654	Katonic acid	n/a	C30H48O3	456.3603454	M-H	455.353069	1
		7.7	0.54584	HMDB0035315	Cucurbitaxanthin A	n/a	C30H48O3	456.3603454	M-H	455.353069	1
		7.7	0.54584	HMDB0036757	Bryonolic acid	n/a	C30H48O3	456.3603454	M-H	455.353069	1
		7.7	0.54584	HMDB0035353	Epoxyganoderiol C	n/a	C30H48O3	456.3603454	M-H	455.353069	1

Table S5. Metabolites derived from 16S predicted metagenomic pathways

pathway_id	pathway	compounds
PWY-7199	pyrimidine deoxyribonucleosides salvage	ammonium
PWY-7199	pyrimidine deoxyribonucleosides salvage	H2O
PWY-7199	pyrimidine deoxyribonucleosides salvage	2'-deoxyuridine
PWY-7199	pyrimidine deoxyribonucleosides salvage	ADP
PWY-7199	pyrimidine deoxyribonucleosides salvage	thymidine
PWY-7199	pyrimidine deoxyribonucleosides salvage	ATP
PWY-7199	pyrimidine deoxyribonucleosides salvage	H+
PWY-7199	pyrimidine deoxyribonucleosides salvage	nucleoside diphosphate
PWY-7199	pyrimidine deoxyribonucleosides salvage	dCMP
PWY-7199	pyrimidine deoxyribonucleosides salvage	2'-deoxycytidine
PWY-7199	pyrimidine deoxyribonucleosides salvage	nucleoside triphosphate
PWY-7199	pyrimidine deoxyribonucleosides salvage	7,8-dihydrofolate
PWY-7199	pyrimidine deoxyribonucleosides salvage	dTMP
PWY-7199	pyrimidine deoxyribonucleosides salvage	5,10-methylenetetrahydrofolate
PWY-7199	pyrimidine deoxyribonucleosides salvage	dUMP
GLCMANNANAUT-PWY	acetylmannosamine and N-acetylneuraminate degradation	ammonium
GLCMANNANAUT-PWY	acetylmannosamine and N-acetylneuraminate degradation	β-D-fructofuranose 6-phosphate
GLCMANNANAUT-PWY	acetylmannosamine and N-acetylneuraminate degradation	α-D-glucosamine 6-phosphate
GLCMANNANAUT-PWY	acetylmannosamine and N-acetylneuraminate degradation	acetate
GLCMANNANAUT-PWY	acetylmannosamine and N-acetylneuraminate degradation	D-glucosamine 6-phosphate
GLCMANNANAUT-PWY	acetylmannosamine and N-acetylneuraminate degradation	H2O
GLCMANNANAUT-PWY	acetylmannosamine and N-acetylneuraminate degradation	N-acetyl-D-glucosamine 6-phosphate
GLCMANNANAUT-PWY	acetylmannosamine and N-acetylneuraminate degradation	pyruvate
GLCMANNANAUT-PWY	acetylmannosamine and N-acetylneuraminate degradation	N-acetylneuraminate
GLCMANNANAUT-PWY	acetylmannosamine and N-acetylneuraminate degradation	ADP
GLCMANNANAUT-PWY	acetylmannosamine and N-acetylneuraminate degradation	N-acetyl-D-mannosamine 6-phosphate
GLCMANNANAUT-PWY	acetylmannosamine and N-acetylneuraminate degradation	H+
GLCMANNANAUT-PWY	acetylmannosamine and N-acetylneuraminate degradation	N-acetyl-D-mannosamine
GLCMANNANAUT-PWY	acetylmannosamine and N-acetylneuraminate degradation	ATP
PWY-6317	galactose degradation I (Leloir pathway)	phosphorylated phosphoglucomutase
PWY-6317	galactose degradation I (Leloir pathway)	α-glucose 1,6-bisphosphate
PWY-6317	galactose degradation I (Leloir pathway)	phosphoglucomutase
PWY-6317	galactose degradation I (Leloir pathway)	ADP
PWY-6317	galactose degradation I (Leloir pathway)	H+
PWY-6317	galactose degradation I (Leloir pathway)	ATP
PWY-6317	galactose degradation I (Leloir pathway)	α-D-galactose 1-phosphate
PWY-6317	galactose degradation I (Leloir pathway)	UDP-α-D-galactose
PWY-6317	galactose degradation I (Leloir pathway)	UDP-α-D-glucose
PWY-6317	galactose degradation I (Leloir pathway)	α-D-galactopyranose
PWY-6317	galactose degradation I (Leloir pathway)	β-D-galactopyranose
PWY-6317	galactose degradation I (Leloir pathway)	D-glucopyranose 6-phosphate
PWY-6317	galactose degradation I (Leloir pathway)	α-D-glucopyranose 1-phosphate
PWY-6897	thiamin salvage II	5-(2-hydroxyethyl)-4-methylthiazole
PWY-6897	thiamin salvage II	diphosphate
PWY-6897	thiamin salvage II	4-methyl-5-(2-phosphooxyethyl)thiazole
PWY-6897	thiamin salvage II	thiamine diphosphate
PWY-6897	thiamin salvage II	thiamine phosphate
PWY-6897	thiamin salvage II	H+
PWY-6897	thiamin salvage II	4-amino-2-methyl-5-pyrimidinemethanol
PWY-6897	thiamin salvage II	ADP
PWY-6897	thiamin salvage II	4-amino-2-methyl-5-(diphosphooxymethyl)pyrimidine
PWY-6897	thiamin salvage II	4-amino-2-methyl-5-(phosphooxymethyl)pyrimidine
PWY-6897	thiamin salvage II	ATP
PWY-621	sucrose degradation III (sucrose invertase)	β-D-fructofuranose 6-phosphate
PWY-621	sucrose degradation III (sucrose invertase)	H+
PWY-621	sucrose degradation III (sucrose invertase)	ADP

PWY-621	sucrose degradation III (sucrose invertase)	D-glucopyranose 6-phosphate
PWY-621	sucrose degradation III (sucrose invertase)	ATP
PWY-621	sucrose degradation III (sucrose invertase)	D-glucopyranose
PWY-621	sucrose degradation III (sucrose invertase)	β-D-fructofuranose
PWY-621	sucrose degradation III (sucrose invertase)	sucrose
PWY-621	sucrose degradation III (sucrose invertase)	H2O
N-PWY	NAD biosynthesis I (from aspartate)	L-glutamate
N-PWY	NAD biosynthesis I (from aspartate)	L-glutamine
N-PWY	NAD biosynthesis I (from aspartate)	NAD+
N-PWY	NAD biosynthesis I (from aspartate)	AMP
N-PWY	NAD biosynthesis I (from aspartate)	ammonium
N-PWY	NAD biosynthesis I (from aspartate)	ATP
N-PWY	NAD biosynthesis I (from aspartate)	nicotinate adenine dinucleotide
N-PWY	NAD biosynthesis I (from aspartate)	5-phospho-α-D-ribose 1-diphosphate
N-PWY	NAD biosynthesis I (from aspartate)	β-nicotinate D-ribonucleotide
N-PWY	NAD biosynthesis I (from aspartate)	diphosphate
N-PWY	NAD biosynthesis I (from aspartate)	CO2
N-PWY	NAD biosynthesis I (from aspartate)	phosphate
N-PWY	NAD biosynthesis I (from aspartate)	H2O
N-PWY	NAD biosynthesis I (from aspartate)	quinolinate
N-PWY	NAD biosynthesis I (from aspartate)	glycerone phosphate
N-PWY	NAD biosynthesis I (from aspartate)	hydrogen peroxide
N-PWY	NAD biosynthesis I (from aspartate)	2-iminosuccinate
N-PWY	NAD biosynthesis I (from aspartate)	H+
N-PWY	NAD biosynthesis I (from aspartate)	L-aspartate
N-PWY	NAD biosynthesis I (from aspartate)	oxygen
PWY-6121	5-aminoimidazole ribonucleotide biosynthesis I	2-(formamido)-N1-(5-phospho-β-D-ribosyl)acetamidine
PWY-6121	5-aminoimidazole ribonucleotide biosynthesis I	5-amino-1-(5-phospho-β-D-ribosyl)imidazole
PWY-6121	5-aminoimidazole ribonucleotide biosynthesis I	5-phospho-α-D-ribose 1-diphosphate
PWY-6121	5-aminoimidazole ribonucleotide biosynthesis I	5-phospho-β-D-ribosylamine
PWY-6121	5-aminoimidazole ribonucleotide biosynthesis I	a tetrahydrofolate
PWY-6121	5-aminoimidazole ribonucleotide biosynthesis I	ADP
PWY-6121	5-aminoimidazole ribonucleotide biosynthesis I	an N10-formyltetrahydrofolate
PWY-6121	5-aminoimidazole ribonucleotide biosynthesis I	ATP
PWY-6121	5-aminoimidazole ribonucleotide biosynthesis I	diphosphate
PWY-6121	5-aminoimidazole ribonucleotide biosynthesis I	glycine
PWY-6121	5-aminoimidazole ribonucleotide biosynthesis I	H+
PWY-6121	5-aminoimidazole ribonucleotide biosynthesis I	H2O
PWY-6121	5-aminoimidazole ribonucleotide biosynthesis I	L-glutamate
PWY-6121	5-aminoimidazole ribonucleotide biosynthesis I	L-glutamine
PWY-6121	5-aminoimidazole ribonucleotide biosynthesis I	N1-(5-phospho-β-D-ribosyl)glycinamide
PWY-6121	5-aminoimidazole ribonucleotide biosynthesis I	N2-formyl-N1-(5-phospho-β-D-ribosyl)glycinamide
PWY-6121	5-aminoimidazole ribonucleotide biosynthesis I	phosphate
PWY-6471	faecium)	glucosaminyl)muramoyl-L-alanyl-γ-O-phospho-D-glutamyl-L-lysyl-D-
PWY-6471	faecium)	ammonia
PWY-6471	faecium)	phosphoenolpyruvate
PWY-6471	faecium)	UDP-N-acetyl-α-D-glucosamine-enolpyruvate
PWY-6471	faecium)	NADPH
PWY-6471	faecium)	NADP+
PWY-6471	faecium)	L-alanine
PWY-6471	faecium)	UDP-N-acetyl-α-D-muramate
PWY-6471	faecium)	UDP-N-acetyl-α-D-muramoyl-L-alanine
PWY-6471	faecium)	D-glutamate
PWY-6471	faecium)	UDP-N-acetyl-α-D-muramoyl-L-alanyl-D-glutamate
PWY-6471	faecium)	L-lysine
PWY-6471	faecium)	UDP-N-acetyl-α-D-muramoyl-L-alanyl-γ-D-glutamyl-L-lysine
PWY-6471	faecium)	D-alanyl-D-alanine
PWY-6471	faecium)	UMP
PWY-6471	faecium)	alanine
PWY-6471	faecium)	di-trans,octa-cis-undecaprenyl phosphate
PWY-6471	faecium)	UDP
PWY-6471	faecium)	lysyl- D-alanyl-D-alanine
PWY-6471	faecium)	UDP-N-acetyl-α-D-glucosamine
PWY-6471	faecium)	D-aspartate
PWY-6471	faecium)	diphosphate
PWY-6471	faecium)	AMP
PWY-6471	faecium)	acetylglucosaminyl)muramoyl-L-alanyl-γ-D-isoglutaminyl-N-(β-D-
PWY-6471	faecium)	ammonium
PWY-6471	faecium)	acetylglucosaminyl)muramoyl-L-alanyl-γ-D-isoglutaminyl-N-(β-D-
PWY-6471	faecium)	D-alanine
PWY-6471	faecium)	a peptidoglycan tetramer with a D,D cross-link (Enterococcus faecium)
PWY-6471	faecium)	a peptidoglycan dimer (E. faeciums)

PWY-6471	faecium)	pentapeptide
PWY-6471	faecium)	di-trans,octa-cis-undecaprenyl diphosphate
PWY-6471	faecium)	H+
PWY-6471	faecium)	phosphate
PWY-6471	faecium)	ADP
PWY-6471	faecium)	acetylglucosaminyl)muramoyl-L-alanyl-γ-D-isoglutaminyl-L-lysyl-D-
PWY-6471	faecium)	L-glutamate
PWY-6471	faecium)	L-glutamine
PWY-6471	faecium)	glucosaminyl)muramoyl-L-alanyl-γ-D-glutamyl-L-lysyl-D-alanyl-D-
PWY-6471	faecium)	ATP
PWY-6471	faecium)	H2O
PWY0-1298	degradation	ammonium
PWY0-1298	degradation	H2O
PWY0-1298	degradation	2'-deoxycytidine
PWY0-1298	degradation	uracil
PWY0-1298	degradation	2'-deoxyuridine
PWY0-1298	degradation	thymine
PWY0-1298	degradation	phosphate
PWY0-1298	degradation	thymidine
PWY0-1298	degradation	2-deoxy-α-D-ribose 1-phosphate
PWY0-1298	degradation	D-glyceraldehyde 3-phosphate
PWY0-1298	degradation	2-deoxy-D-ribose 5-phosphate
PWY0-1298	degradation	H+
PWY0-1298	degradation	NADH
PWY0-1298	degradation	acetyl-CoA
PWY0-1298	degradation	acetaldehyde
PWY0-1298	degradation	coenzyme A
PWY0-1298	degradation	NAD+
PWY-6387	biosynthesis I (meso-diaminopimelate containing)	UDP-N-acetyl-α-D-glucosamine
PWY-6387	biosynthesis I (meso-diaminopimelate containing)	phosphoenolpyruvate
PWY-6387	biosynthesis I (meso-diaminopimelate containing)	UDP-N-acetyl-α-D-glucosamine-enolpyruvate
PWY-6387	biosynthesis I (meso-diaminopimelate containing)	NADPH
PWY-6387	biosynthesis I (meso-diaminopimelate containing)	NADP+
PWY-6387	biosynthesis I (meso-diaminopimelate containing)	diaminopimeloyl-D-alanyl-D-alanine
PWY-6387	biosynthesis I (meso-diaminopimelate containing)	L-alanine
PWY-6387	biosynthesis I (meso-diaminopimelate containing)	UDP-N-acetyl-α-D-muramate
PWY-6387	biosynthesis I (meso-diaminopimelate containing)	UDP-N-acetyl-α-D-muramoyl-L-alanine
PWY-6387	biosynthesis I (meso-diaminopimelate containing)	D-glutamate
PWY-6387	biosynthesis I (meso-diaminopimelate containing)	L-glutamate
PWY-6387	biosynthesis I (meso-diaminopimelate containing)	diaminopimelate
PWY-6387	biosynthesis I (meso-diaminopimelate containing)	UDP-N-acetyl-α-D-muramoyl-L-alanyl-D-glutamate
PWY-6387	biosynthesis I (meso-diaminopimelate containing)	meso-diaminopimelate
PWY-6387	biosynthesis I (meso-diaminopimelate containing)	ADP
PWY-6387	biosynthesis I (meso-diaminopimelate containing)	phosphate
PWY-6387	biosynthesis I (meso-diaminopimelate containing)	D-alanyl-D-alanine
PWY-6387	biosynthesis I (meso-diaminopimelate containing)	H+
PWY-6387	biosynthesis I (meso-diaminopimelate containing)	D-alanine
PWY-6387	biosynthesis I (meso-diaminopimelate containing)	ATP
CANSYN-PWY	diaminopimelate containing)	UDP
CANSYN-PWY	diaminopimelate containing)	(1,4)-]-N-acetyl-α-D-muramoyl-L-alanyl-γ-D-glutamyl-meso-2,6-
CANSYN-PWY	diaminopimelate containing)	UMP
CANSYN-PWY	diaminopimelate containing)	meso-2,6-diaminopimeloyl-D-alanyl-D-alanine
CANSYN-PWY	diaminopimelate containing)	di-trans,octa-cis-undecaprenyl phosphate
CANSYN-PWY	diaminopimelate containing)	UDP-N-acetyl-α-D-glucosamine
CANSYN-PWY	diaminopimelate containing)	phosphoenolpyruvate
CANSYN-PWY	diaminopimelate containing)	UDP-N-acetyl-α-D-glucosamine-enolpyruvate
CANSYN-PWY	diaminopimelate containing)	NADPH
CANSYN-PWY	diaminopimelate containing)	NADP+
CANSYN-PWY	diaminopimelate containing)	diaminopimeloyl-D-alanyl-D-alanine
CANSYN-PWY	diaminopimelate containing)	L-alanine
CANSYN-PWY	diaminopimelate containing)	UDP-N-acetyl-α-D-muramate
CANSYN-PWY	diaminopimelate containing)	UDP-N-acetyl-α-D-muramoyl-L-alanine
CANSYN-PWY	diaminopimelate containing)	D-glutamate
CANSYN-PWY	diaminopimelate containing)	L-glutamate
CANSYN-PWY	diaminopimelate containing)	diaminopimelate
CANSYN-PWY	diaminopimelate containing)	UDP-N-acetyl-α-D-muramoyl-L-alanyl-D-glutamate
CANSYN-PWY	diaminopimelate containing)	meso-diaminopimelate
CANSYN-PWY	diaminopimelate containing)	ADP
CANSYN-PWY	diaminopimelate containing)	phosphate
CANSYN-PWY	diaminopimelate containing)	D-alanyl-D-alanine
CANSYN-PWY	diaminopimelate containing)	H+
CANSYN-PWY	diaminopimelate containing)	D-alanine

CANSYN-PWY	diaminopimelate containing)	ATP
PANTO-PWY	phosphopantothenate biosynthesis I	AMP
PANTO-PWY	phosphopantothenate biosynthesis I	diphosphate
PANTO-PWY	phosphopantothenate biosynthesis I	β-alanine
PANTO-PWY	phosphopantothenate biosynthesis I	NADPH
PANTO-PWY	phosphopantothenate biosynthesis I	(R)-pantoate
PANTO-PWY	phosphopantothenate biosynthesis I	NADP+
PANTO-PWY	phosphopantothenate biosynthesis I	a tetrahydrofolate
PANTO-PWY	phosphopantothenate biosynthesis I	2-dehydropantoate
PANTO-PWY	phosphopantothenate biosynthesis I	a 5,10-methylenetetrahydrofolate
PANTO-PWY	phosphopantothenate biosynthesis I	3-methyl-2-oxobutanoate
PANTO-PWY	phosphopantothenate biosynthesis I	H2O
PANTO-PWY	phosphopantothenate biosynthesis I	ADP
PANTO-PWY	phosphopantothenate biosynthesis I	(R)-4'-phosphopantothenate
PANTO-PWY	phosphopantothenate biosynthesis I	H+
PANTO-PWY	phosphopantothenate biosynthesis I	(R)-pantothenate
PANTO-PWY	phosphopantothenate biosynthesis I	ATP
PWY0-1296	purine ribonucleosides degradation	AMP
PWY0-1296	purine ribonucleosides degradation	diphosphate
PWY0-1296	purine ribonucleosides degradation	β-alanine
PWY0-1296	purine ribonucleosides degradation	NADPH
PWY0-1296	purine ribonucleosides degradation	(R)-pantoate
PWY0-1296	purine ribonucleosides degradation	NADP+
PWY0-1296	purine ribonucleosides degradation	a tetrahydrofolate
PWY0-1296	purine ribonucleosides degradation	2-dehydropantoate
PWY0-1296	purine ribonucleosides degradation	a 5,10-methylenetetrahydrofolate
PWY0-1296	purine ribonucleosides degradation	3-methyl-2-oxobutanoate
PWY0-1296	purine ribonucleosides degradation	H2O
PWY0-1296	purine ribonucleosides degradation	ADP
PWY0-1296	purine ribonucleosides degradation	(R)-4'-phosphopantothenate
PWY0-1296	purine ribonucleosides degradation	H+
PWY0-1296	purine ribonucleosides degradation	(R)-pantothenate
PWY0-1296	purine ribonucleosides degradation	ATP
PWY-6703	preQ0 biosynthesis	formate
PWY-6703	preQ0 biosynthesis	GTP
PWY-6703	preQ0 biosynthesis	triphosphate
PWY-6703	preQ0 biosynthesis	acetaldehyde
PWY-6703	preQ0 biosynthesis	7,8-dihydroneopterin 3'-triphosphate
PWY-6703	preQ0 biosynthesis	6-carboxy-5,6,7,8-tetrahydropterin
PWY-6703	preQ0 biosynthesis	H2O
PWY-6703	preQ0 biosynthesis	phosphate
PWY-6703	preQ0 biosynthesis	ADP
PWY-6703	preQ0 biosynthesis	preQ0
PWY-6703	preQ0 biosynthesis	H+
PWY-6703	preQ0 biosynthesis	7-carboxy-7-deazaguanine
PWY-6703	preQ0 biosynthesis	ammonium
PWY-6703	preQ0 biosynthesis	ATP
PWY-6385	peptidoglycan biosynthesis III (mycobacteria)	UMP
PWY-6385	peptidoglycan biosynthesis III (mycobacteria)	trans,octacis-decaprenyl phosphate
PWY-6385	peptidoglycan biosynthesis III (mycobacteria)	UDP
PWY-6385	peptidoglycan biosynthesis III (mycobacteria)	decaprenyl-diphospho-N-acetylmuramoyl-pentapeptide
PWY-6385	peptidoglycan biosynthesis III (mycobacteria)	UDP-N-acetyl-α-D-glucosamine
PWY-6385	peptidoglycan biosynthesis III (mycobacteria)	phosphoenolpyruvate
PWY-6385	peptidoglycan biosynthesis III (mycobacteria)	UDP-N-acetyl-α-D-glucosamine-enolpyruvate
PWY-6385	peptidoglycan biosynthesis III (mycobacteria)	NADPH
PWY-6385	peptidoglycan biosynthesis III (mycobacteria)	NADP+
PWY-6385	peptidoglycan biosynthesis III (mycobacteria)	diaminopimeloyl-D-alanyl-D-alanine
PWY-6385	peptidoglycan biosynthesis III (mycobacteria)	L-alanine
PWY-6385	peptidoglycan biosynthesis III (mycobacteria)	UDP-N-acetyl-α-D-muramate
PWY-6385	peptidoglycan biosynthesis III (mycobacteria)	UDP-N-acetyl-α-D-muramoyl-L-alanine
PWY-6385	peptidoglycan biosynthesis III (mycobacteria)	D-glutamate
PWY-6385	peptidoglycan biosynthesis III (mycobacteria)	L-glutamate
PWY-6385	peptidoglycan biosynthesis III (mycobacteria)	diaminopimelate
PWY-6385	peptidoglycan biosynthesis III (mycobacteria)	UDP-N-acetyl-α-D-muramoyl-L-alanyl-D-glutamate
PWY-6385	peptidoglycan biosynthesis III (mycobacteria)	meso-diaminopimelate
PWY-6385	peptidoglycan biosynthesis III (mycobacteria)	ADP
PWY-6385	peptidoglycan biosynthesis III (mycobacteria)	phosphate
PWY-6385	peptidoglycan biosynthesis III (mycobacteria)	D-alanyl-D-alanine
PWY-6385	peptidoglycan biosynthesis III (mycobacteria)	ATP
PWY-6385	peptidoglycan biosynthesis III (mycobacteria)	a peptidoglycan with L,D cross-links (mycobacteria)
PWY-6385	peptidoglycan biosynthesis III (mycobacteria)	D-alanine
PWY-6385	peptidoglycan biosynthesis III (mycobacteria)	a peptidoglycan with D,D cross-links (mycobacteria)

PWY-6385	peptidoglycan biosynthesis III (mycobacteria)	H2O
PWY-6385	peptidoglycan biosynthesis III (mycobacteria)	mono-trans,octa-cis-decaprenyl diphosphate
PWY-6385	peptidoglycan biosynthesis III (mycobacteria)	a peptidoglycan dimer (mycobacteria)
PWY-6385	peptidoglycan biosynthesis III (mycobacteria)	H+
PWY-6385	peptidoglycan biosynthesis III (mycobacteria)	(pentapeptide)
PWY-6385	peptidoglycan biosynthesis III (mycobacteria)	(1,4)-]-N-acetyl-α-D-muramoyl-L-alanyl-γ-D-glutamyl-meso-2,6-
PWY-6385	peptidoglycan biosynthesis III (mycobacteria)	di-trans,octa-cis-undecaprenyl diphosphate
PWY-6478	biosynthesis	D-sedoheptulose 7-phosphate
PWY-6478	biosynthesis	ADP
PWY-6478	biosynthesis	D-glycero-D-manno-heptose 7-phosphate
PWY-6478	biosynthesis	ATP
PWY-6478	biosynthesis	phosphate
PWY-6478	biosynthesis	D-glycero-α-D-manno-heptose 1,7-bisphosphate
PWY-6478	biosynthesis	H2O
PWY-6478	biosynthesis	diphosphate
PWY-6478	biosynthesis	GDP-D-glycero-α-D-manno-heptose
PWY-6478	biosynthesis	H+
PWY-6478	biosynthesis	D-glycero-α-D-manno-heptose 1-phosphate
PWY-6478	biosynthesis	GTP
PWY-6151	S-adenosyl-L-methionine cycle I	adenine
PWY-6151	S-adenosyl-L-methionine cycle I	autoinducer 2
PWY-6151	S-adenosyl-L-methionine cycle I	S-ribosyl-L-homocysteine
PWY-6151	S-adenosyl-L-methionine cycle I	diphosphate
PWY-6151	S-adenosyl-L-methionine cycle I	phosphate
PWY-6151	S-adenosyl-L-methionine cycle I	ATP
PWY-6151	S-adenosyl-L-methionine cycle I	H2O
PWY-6151	S-adenosyl-L-methionine cycle I	H+
PWY-6151	S-adenosyl-L-methionine cycle I	a methylated methyl donor
PWY-6151	S-adenosyl-L-methionine cycle I	S-adenosyl-L-homocysteine
PWY-6151	S-adenosyl-L-methionine cycle I	S-adenosyl-L-methionine
PWY-6151	S-adenosyl-L-methionine cycle I	a demethylated methyl donor
PWY-6151	S-adenosyl-L-methionine cycle I	L-methionine
PWY-6151	S-adenosyl-L-methionine cycle I	tetrahydropteroyl tri-L-glutamate
PWY-6151	S-adenosyl-L-methionine cycle I	5-methyltetrahydropteroyl tri-L-glutamate
PWY-6151	S-adenosyl-L-methionine cycle I	L-homocysteine
PWY	flavin biosynthesis I (bacteria and plants)	ADP
PWY	flavin biosynthesis I (bacteria and plants)	FAD
PWY	flavin biosynthesis I (bacteria and plants)	FMN
PWY	flavin biosynthesis I (bacteria and plants)	ATP
PWY	flavin biosynthesis I (bacteria and plants)	riboflavin
PWY	flavin biosynthesis I (bacteria and plants)	6,7-dimethyl-8-(1-D-ribityl)lumazine
PWY	flavin biosynthesis I (bacteria and plants)	phosphate
PWY	flavin biosynthesis I (bacteria and plants)	5-amino-6-(D-ribitylamino)uracil
PWY	flavin biosynthesis I (bacteria and plants)	1-deoxy-L-glycero-tetrulose 4-phosphate
PWY	flavin biosynthesis I (bacteria and plants)	D-ribulose 5-phosphate
PWY	flavin biosynthesis I (bacteria and plants)	NADPH
PWY	flavin biosynthesis I (bacteria and plants)	5-amino-6-(5-phospho-D-ribitylamino)uracil
PWY	flavin biosynthesis I (bacteria and plants)	NADP+
PWY	flavin biosynthesis I (bacteria and plants)	ammonium
PWY	flavin biosynthesis I (bacteria and plants)	5-amino-6-(5-phospho-D-ribosylamino)uracil
PWY	flavin biosynthesis I (bacteria and plants)	formate
PWY	flavin biosynthesis I (bacteria and plants)	2,5-diamino-6-(5-phospho-D-ribosylamino)pyrimidin-4(3H)-one
PWY	flavin biosynthesis I (bacteria and plants)	diphosphate
PWY	flavin biosynthesis I (bacteria and plants)	H+
PWY	flavin biosynthesis I (bacteria and plants)	H2O
PWY	flavin biosynthesis I (bacteria and plants)	GTP
PWY	UDP-N-acetyl-D-glucosamine biosynthesis I	phosphorylated phosphoglucosamine mutase
PWY	UDP-N-acetyl-D-glucosamine biosynthesis I	glucosamine 1,6-diphosphate
PWY	UDP-N-acetyl-D-glucosamine biosynthesis I	phosphoglucosamine mutase
PWY	UDP-N-acetyl-D-glucosamine biosynthesis I	diphosphate
PWY	UDP-N-acetyl-D-glucosamine biosynthesis I	UDP-N-acetyl-α-D-glucosamine
PWY	UDP-N-acetyl-D-glucosamine biosynthesis I	UTP
PWY	UDP-N-acetyl-D-glucosamine biosynthesis I	H+
PWY	UDP-N-acetyl-D-glucosamine biosynthesis I	coenzyme A
PWY	UDP-N-acetyl-D-glucosamine biosynthesis I	N-acetyl-α-D-glucosamine 1-phosphate
PWY	UDP-N-acetyl-D-glucosamine biosynthesis I	acetyl-CoA
PWY	UDP-N-acetyl-D-glucosamine biosynthesis I	L-glutamate
PWY	UDP-N-acetyl-D-glucosamine biosynthesis I	L-glutamine
PWY	UDP-N-acetyl-D-glucosamine biosynthesis I	α-D-glucosamine 1-phosphate
PWY	UDP-N-acetyl-D-glucosamine biosynthesis I	D-glucosamine 6-phosphate
PWY	UDP-N-acetyl-D-glucosamine biosynthesis I	β-D-fructofuranose 6-phosphate
PWY	UDP-N-acetyl-D-glucosamine biosynthesis I	D-glucopyranose 6-phosphate

PWY	O-antigen building blocks biosynthesis (E. coli)	phosphorylated phosphoglucosamine mutase
PWY	O-antigen building blocks biosynthesis (E. coli)	glucosamine 1,6-diphosphate
PWY	O-antigen building blocks biosynthesis (E. coli)	phosphoglucosamine mutase
PWY	O-antigen building blocks biosynthesis (E. coli)	UDP-α-D-galactofuranose
PWY	O-antigen building blocks biosynthesis (E. coli)	UDP-α-D-galactose
PWY	O-antigen building blocks biosynthesis (E. coli)	NADPH
PWY	O-antigen building blocks biosynthesis (E. coli)	dTDP-4-dehydro-β-L-rhamnose
PWY	O-antigen building blocks biosynthesis (E. coli)	dTDP-β-L-rhamnose
PWY	O-antigen building blocks biosynthesis (E. coli)	NADP+
PWY	O-antigen building blocks biosynthesis (E. coli)	dTTP
PWY	O-antigen building blocks biosynthesis (E. coli)	α-D-glucopyranose 1-phosphate
PWY	O-antigen building blocks biosynthesis (E. coli)	dTDP-4-dehydro-6-deoxy-α-D-glucopyranose
PWY	O-antigen building blocks biosynthesis (E. coli)	H2O
PWY	O-antigen building blocks biosynthesis (E. coli)	dTDP-α-D-glucose
PWY	O-antigen building blocks biosynthesis (E. coli)	diphosphate
PWY	O-antigen building blocks biosynthesis (E. coli)	UDP-N-acetyl-α-D-glucosamine
PWY	O-antigen building blocks biosynthesis (E. coli)	UTP
PWY	O-antigen building blocks biosynthesis (E. coli)	H+
PWY	O-antigen building blocks biosynthesis (E. coli)	coenzyme A
PWY	O-antigen building blocks biosynthesis (E. coli)	N-acetyl-α-D-glucosamine 1-phosphate
PWY	O-antigen building blocks biosynthesis (E. coli)	acetyl-CoA
PWY	O-antigen building blocks biosynthesis (E. coli)	L-glutamate
PWY	O-antigen building blocks biosynthesis (E. coli)	L-glutamine
PWY	O-antigen building blocks biosynthesis (E. coli)	α-D-glucosamine 1-phosphate
PWY	O-antigen building blocks biosynthesis (E. coli)	D-glucosamine 6-phosphate
PWY	O-antigen building blocks biosynthesis (E. coli)	β-D-fructofuranose 6-phosphate
PWY	O-antigen building blocks biosynthesis (E. coli)	D-glucopyranose 6-phosphate
ON-PWY	mixed acid fermentation	an oxidized hydrogenase 3
ON-PWY	mixed acid fermentation	a reduced hydrogenase 3
ON-PWY	mixed acid fermentation	oxalosuccinate
ON-PWY	mixed acid fermentation	acetyl phosphate
ON-PWY	mixed acid fermentation	acetate
ON-PWY	mixed acid fermentation	acetaldehyde
ON-PWY	mixed acid fermentation	ethanol
ON-PWY	mixed acid fermentation	ADP
ON-PWY	mixed acid fermentation	ATP
ON-PWY	mixed acid fermentation	pyruvate
ON-PWY	mixed acid fermentation	(R)-lactate
ON-PWY	mixed acid fermentation	hydrogencarbonate
ON-PWY	mixed acid fermentation	phosphoenolpyruvate
ON-PWY	mixed acid fermentation	phosphate
ON-PWY	mixed acid fermentation	H2
ON-PWY	mixed acid fermentation	formate
ON-PWY	mixed acid fermentation	succinate
ON-PWY	mixed acid fermentation	a menaquinone
ON-PWY	mixed acid fermentation	a menaquinol
ON-PWY	mixed acid fermentation	fumarate
ON-PWY	mixed acid fermentation	coenzyme A
ON-PWY	mixed acid fermentation	acetyl-CoA
ON-PWY	mixed acid fermentation	citrate
ON-PWY	mixed acid fermentation	cis-aconitate
ON-PWY	mixed acid fermentation	H2O
ON-PWY	mixed acid fermentation	NADPH
ON-PWY	mixed acid fermentation	CO2
ON-PWY	mixed acid fermentation	2-oxoglutarate
ON-PWY	mixed acid fermentation	D-threo-isocitrate
ON-PWY	mixed acid fermentation	NADP+
ON-PWY	mixed acid fermentation	H+
ON-PWY	mixed acid fermentation	NADH
ON-PWY	mixed acid fermentation	oxaloacetate
ON-PWY	mixed acid fermentation	(S)-malate
ON-PWY	mixed acid fermentation	NAD+
PWY-6122	5-aminoimidazole ribonucleotide biosynthesis II	5-phospho-α-D-ribose 1-diphosphate
PWY-6122	5-aminoimidazole ribonucleotide biosynthesis II	diphosphate
PWY-6122	5-aminoimidazole ribonucleotide biosynthesis II	5-phospho-β-D-ribosylamine
PWY-6122	5-aminoimidazole ribonucleotide biosynthesis II	glycine
PWY-6122	5-aminoimidazole ribonucleotide biosynthesis II	L-glutamate
PWY-6122	5-aminoimidazole ribonucleotide biosynthesis II	L-glutamine
PWY-6122	5-aminoimidazole ribonucleotide biosynthesis II	H2O
PWY-6122	5-aminoimidazole ribonucleotide biosynthesis II	5-amino-1-(5-phospho-β-D-ribosyl)imidazole
PWY-6122	5-aminoimidazole ribonucleotide biosynthesis II	2-(formamido)-N1-(5-phospho-β-D-ribosyl)acetamidine
PWY-6122	5-aminoimidazole ribonucleotide biosynthesis II	phosphate

PWY-6122	5-aminoimidazole ribonucleotide biosynthesis II	ADP
PWY-6122	5-aminoimidazole ribonucleotide biosynthesis II	N2-formyl-N1-(5-phospho-β-D-ribosyl)glycinamide
PWY-6122	5-aminoimidazole ribonucleotide biosynthesis II	H+
PWY-6122	5-aminoimidazole ribonucleotide biosynthesis II	N1-(5-phospho-β-D-ribosyl)glycinamide
PWY-6122	5-aminoimidazole ribonucleotide biosynthesis II	formate
PWY-6122	5-aminoimidazole ribonucleotide biosynthesis II	ATP
PWY-6277	biosynthesis	5-phospho-α-D-ribose 1-diphosphate
PWY-6277	biosynthesis	diphosphate
PWY-6277	biosynthesis	5-phospho-β-D-ribosylamine
PWY-6277	biosynthesis	glycine
PWY-6277	biosynthesis	L-glutamate
PWY-6277	biosynthesis	L-glutamine
PWY-6277	biosynthesis	H2O
PWY-6277	biosynthesis	5-amino-1-(5-phospho-β-D-ribosyl)imidazole
PWY-6277	biosynthesis	2-(formamido)-N1-(5-phospho-β-D-ribosyl)acetamidine
PWY-6277	biosynthesis	phosphate
PWY-6277	biosynthesis	ADP
PWY-6277	biosynthesis	formate
PWY-6277	biosynthesis	ATP
PWY-6277	biosynthesis	H+
PWY-6277	biosynthesis	N2-formyl-N1-(5-phospho-β-D-ribosyl)glycinamide
PWY-6277	biosynthesis	a tetrahydrofolate
PWY-6277	biosynthesis	an N10-formyltetrahydrofolate
PWY-6277	biosynthesis	N1-(5-phospho-β-D-ribosyl)glycinamide
PWY-6612	superpathway of tetrahydrofolate biosynthesis	ADP
PWY-6612	superpathway of tetrahydrofolate biosynthesis	7,8-dihydrofolate monoglutamate
PWY-6612	superpathway of tetrahydrofolate biosynthesis	7,8-dihydropteroate
PWY-6612	superpathway of tetrahydrofolate biosynthesis	NADPH
PWY-6612	superpathway of tetrahydrofolate biosynthesis	a 7,8-dihydrofolate
PWY-6612	superpathway of tetrahydrofolate biosynthesis	a tetrahydrofolate
PWY-6612	superpathway of tetrahydrofolate biosynthesis	NADP+
PWY-6612	superpathway of tetrahydrofolate biosynthesis	AMP
PWY-6612	superpathway of tetrahydrofolate biosynthesis	(7,8-dihydropterin-6-yl)methyl diphosphate
PWY-6612	superpathway of tetrahydrofolate biosynthesis	ATP
PWY-6612	superpathway of tetrahydrofolate biosynthesis	6-(hydroxymethyl)-7,8-dihydropterin
PWY-6612	superpathway of tetrahydrofolate biosynthesis	glycolaldehyde
PWY-6612	superpathway of tetrahydrofolate biosynthesis	phosphate
PWY-6612	superpathway of tetrahydrofolate biosynthesis	D-erythro-7,8-dihydroneopterin
PWY-6612	superpathway of tetrahydrofolate biosynthesis	diphosphate
PWY-6612	superpathway of tetrahydrofolate biosynthesis	7,8-dihydroneopterin 3'-phosphate
PWY-6612	superpathway of tetrahydrofolate biosynthesis	7,8-dihydroneopterin 3'-triphosphate
PWY-6612	superpathway of tetrahydrofolate biosynthesis	formate
PWY-6612	superpathway of tetrahydrofolate biosynthesis	GTP
PWY-6612	superpathway of tetrahydrofolate biosynthesis	H2O
PWY-6612	superpathway of tetrahydrofolate biosynthesis	L-glutamate
PWY-6612	superpathway of tetrahydrofolate biosynthesis	chorismate
PWY-6612	superpathway of tetrahydrofolate biosynthesis	L-glutamine
PWY-6612	superpathway of tetrahydrofolate biosynthesis	pyruvate
PWY-6612	superpathway of tetrahydrofolate biosynthesis	4-aminobenzoate
PWY-6612	superpathway of tetrahydrofolate biosynthesis	H+
PWY-6612	superpathway of tetrahydrofolate biosynthesis	4-amino-4-deoxychorismate
PWY	pantothenate and coenzyme A biosynthesis I	AMP
PWY	pantothenate and coenzyme A biosynthesis I	NADPH
PWY	pantothenate and coenzyme A biosynthesis I	(R)-pantoate
PWY	pantothenate and coenzyme A biosynthesis I	NADP+
PWY	pantothenate and coenzyme A biosynthesis I	a tetrahydrofolate
PWY	pantothenate and coenzyme A biosynthesis I	2-dehydropantoate
PWY	pantothenate and coenzyme A biosynthesis I	a 5,10-methylenetetrahydrofolate
PWY	pantothenate and coenzyme A biosynthesis I	3-methyl-2-oxobutanoate
PWY	pantothenate and coenzyme A biosynthesis I	H2O
PWY	pantothenate and coenzyme A biosynthesis I	(R)-pantothenate
PWY	pantothenate and coenzyme A biosynthesis I	ADP
PWY	pantothenate and coenzyme A biosynthesis I	coenzyme A
PWY	pantothenate and coenzyme A biosynthesis I	3'-dephospho-CoA
PWY	pantothenate and coenzyme A biosynthesis I	ATP
PWY	pantothenate and coenzyme A biosynthesis I	4'-phosphopantetheine
PWY	pantothenate and coenzyme A biosynthesis I	CMP
PWY	pantothenate and coenzyme A biosynthesis I	diphosphate
PWY	pantothenate and coenzyme A biosynthesis I	(R)-4'-phosphopantothenoyl-L-cysteine
PWY	pantothenate and coenzyme A biosynthesis I	(R)-4'-phosphopantothenate
PWY	pantothenate and coenzyme A biosynthesis I	L-cysteine
PWY	pantothenate and coenzyme A biosynthesis I	CTP

PWY	pantothenate and coenzyme A biosynthesis I	CO_2
PWY	pantothenate and coenzyme A biosynthesis I	β-alanine
PWY	pantothenate and coenzyme A biosynthesis I	H^+
PWY	pantothenate and coenzyme A biosynthesis I	L-aspartate
TCA	TCA cycle I (prokaryotic)	a [2-oxoglutarate dehydrogenase E2 protein] N6-lipoyl-L-lysine
TCA	TCA cycle I (prokaryotic)	succinyldihydrolipoyl-L-lysine
TCA	TCA cycle I (prokaryotic)	a [2-oxoglutarate dehydrogenase E2 protein] N6-dihydrolipoyl-L-lysine
TCA	TCA cycle I (prokaryotic)	oxalosuccinate
TCA	TCA cycle I (prokaryotic)	phosphate
TCA	TCA cycle I (prokaryotic)	ADP
TCA	TCA cycle I (prokaryotic)	ATP
TCA	TCA cycle I (prokaryotic)	succinyl-CoA
TCA	TCA cycle I (prokaryotic)	coenzyme A
TCA	TCA cycle I (prokaryotic)	acetyl-CoA
TCA	TCA cycle I (prokaryotic)	H_2O
TCA	TCA cycle I (prokaryotic)	cis-aconitate
TCA	TCA cycle I (prokaryotic)	citrate
TCA	TCA cycle I (prokaryotic)	NADPH
TCA	TCA cycle I (prokaryotic)	CO_2
TCA	TCA cycle I (prokaryotic)	2-oxoglutarate
TCA	TCA cycle I (prokaryotic)	D-threo-isocitrate
TCA	TCA cycle I (prokaryotic)	$NADP^+$
TCA	TCA cycle I (prokaryotic)	H^+
TCA	TCA cycle I (prokaryotic)	NADH
TCA	TCA cycle I (prokaryotic)	oxaloacetate
TCA	TCA cycle I (prokaryotic)	(S)-malate
TCA	TCA cycle I (prokaryotic)	NAD^+
TCA	TCA cycle I (prokaryotic)	fumarate
TCA	TCA cycle I (prokaryotic)	an electron-transfer quinol
TCA	TCA cycle I (prokaryotic)	an electron-transfer quinone
TCA	TCA cycle I (prokaryotic)	succinate
PWY-6969	oxidoreductase)	oxalosuccinate
PWY-6969	oxidoreductase)	citrate
PWY-6969	oxidoreductase)	cis-aconitate
PWY-6969	oxidoreductase)	acetyl-CoA
PWY-6969	oxidoreductase)	H_2O
PWY-6969	oxidoreductase)	glyoxylate
PWY-6969	oxidoreductase)	NADPH
PWY-6969	oxidoreductase)	D-threo-isocitrate
PWY-6969	oxidoreductase)	$NADP^+$
PWY-6969	oxidoreductase)	a reduced ferredoxin [iron-sulfur] cluster
PWY-6969	oxidoreductase)	CO_2
PWY-6969	oxidoreductase)	an oxidized ferredoxin [iron-sulfur] cluster
PWY-6969	oxidoreductase)	a deaminated amino group donor
PWY-6969	oxidoreductase)	L-glutamate
PWY-6969	oxidoreductase)	2-oxoglutarate
PWY-6969	oxidoreductase)	an aminated amino group donor
PWY-6969	oxidoreductase)	phosphate
PWY-6969	oxidoreductase)	ADP
PWY-6969	oxidoreductase)	succinyl-CoA
PWY-6969	oxidoreductase)	coenzyme A
PWY-6969	oxidoreductase)	ATP
PWY-6969	oxidoreductase)	H^+
PWY-6969	oxidoreductase)	NADH
PWY-6969	oxidoreductase)	oxaloacetate
PWY-6969	oxidoreductase)	(S)-malate
PWY-6969	oxidoreductase)	NAD^+
PWY-6969	oxidoreductase)	fumarate
PWY-6969	oxidoreductase)	an electron-transfer quinol
PWY-6969	oxidoreductase)	an electron-transfer quinone
PWY-6969	oxidoreductase)	succinate
PWY-6700	queuosine biosynthesis	NADPH
PWY-6700	queuosine biosynthesis	preQ0
PWY-6700	queuosine biosynthesis	$NADP^+$
PWY-6700	queuosine biosynthesis	guanine
PWY-6700	queuosine biosynthesis	a guanine34 in tRNA
PWY-6700	queuosine biosynthesis	preQ1
PWY-6700	queuosine biosynthesis	H^+
PWY-6700	queuosine biosynthesis	L-methionine
PWY-6700	queuosine biosynthesis	adenine
PWY-6700	queuosine biosynthesis	S-adenosyl-L-methionine
PWY-6700	queuosine biosynthesis	a 7-aminomethyl-7-deazaguanosine34 in tRNA

PWY-6700	queuosine biosynthesis	a reduced unknown electron carrier
PWY-6700	queuosine biosynthesis	an epoxyqueuosine34 in tRNA
PWY-6700	queuosine biosynthesis	a queuosine34 in tRNA
PWY-6700	queuosine biosynthesis	an oxidized unknown electron carrier
PWY-6700	queuosine biosynthesis	H2O
PWY-5659	GDP-mannose biosynthesis	D-mannopyranose 6-phosphate
PWY-5659	GDP-mannose biosynthesis	diphosphate
PWY-5659	GDP-mannose biosynthesis	GDP-α-D-mannose
PWY-5659	GDP-mannose biosynthesis	H+
PWY-5659	GDP-mannose biosynthesis	α-D-mannose 1-phosphate
PWY-5659	GDP-mannose biosynthesis	GTP
PWY-5659	GDP-mannose biosynthesis	β-D-fructofuranose 6-phosphate
PWY-5659	GDP-mannose biosynthesis	D-glucopyranose 6-phosphate
P108-PWY	pyruvate fermentation to propanoate I	H2O
P108-PWY	pyruvate fermentation to propanoate I	a menaquinone
P108-PWY	pyruvate fermentation to propanoate I	a menaquinol
P108-PWY	pyruvate fermentation to propanoate I	fumarate
P108-PWY	pyruvate fermentation to propanoate I	propanoate
P108-PWY	pyruvate fermentation to propanoate I	succinate
P108-PWY	pyruvate fermentation to propanoate I	succinyl-CoA
P108-PWY	pyruvate fermentation to propanoate I	(R)-methylmalonyl-CoA
P108-PWY	pyruvate fermentation to propanoate I	propanoyl-CoA
P108-PWY	pyruvate fermentation to propanoate I	pyruvate
P108-PWY	pyruvate fermentation to propanoate I	(S)-methylmalonyl-CoA
P108-PWY	pyruvate fermentation to propanoate I	H+
P108-PWY	pyruvate fermentation to propanoate I	NADH
P108-PWY	pyruvate fermentation to propanoate I	oxaloacetate
P108-PWY	pyruvate fermentation to propanoate I	(S)-malate
P108-PWY	pyruvate fermentation to propanoate I	NAD+
PWY-6386	biosynthesis II (lysine-containing)	UDP-N-acetyl-α-D-glucosamine
PWY-6386	biosynthesis II (lysine-containing)	phosphoenolpyruvate
PWY-6386	biosynthesis II (lysine-containing)	UDP-N-acetyl-α-D-glucosamine-enolpyruvate
PWY-6386	biosynthesis II (lysine-containing)	NADPH
PWY-6386	biosynthesis II (lysine-containing)	NADP+
PWY-6386	biosynthesis II (lysine-containing)	L-alanine
PWY-6386	biosynthesis II (lysine-containing)	UDP-N-acetyl-α-D-muramate
PWY-6386	biosynthesis II (lysine-containing)	UDP-N-acetyl-α-D-muramoyl-L-alanine
PWY-6386	biosynthesis II (lysine-containing)	D-glutamate
PWY-6386	biosynthesis II (lysine-containing)	L-glutamate
PWY-6386	biosynthesis II (lysine-containing)	D-alanine
PWY-6386	biosynthesis II (lysine-containing)	UDP-N-acetyl-α-D-muramoyl-L-alanyl-D-glutamate
PWY-6386	biosynthesis II (lysine-containing)	L-lysine
PWY-6386	biosynthesis II (lysine-containing)	ADP
PWY-6386	biosynthesis II (lysine-containing)	phosphate
PWY-6386	biosynthesis II (lysine-containing)	alanine
PWY-6386	biosynthesis II (lysine-containing)	H+
PWY-6386	biosynthesis II (lysine-containing)	UDP-N-acetyl-α-D-muramoyl-L-alanyl-γ-D-glutamyl-L-lysine
PWY-6386	biosynthesis II (lysine-containing)	D-alanyl-D-alanine
PWY-6386	biosynthesis II (lysine-containing)	ATP
P161-PWY	acetylene degradation	acetylene
P161-PWY	acetylene degradation	H2O
P161-PWY	acetylene degradation	coenzyme A
P161-PWY	acetylene degradation	phosphate
P161-PWY	acetylene degradation	acetyl-CoA
P161-PWY	acetylene degradation	acetyl phosphate
P161-PWY	acetylene degradation	ADP
P161-PWY	acetylene degradation	ATP
P161-PWY	acetylene degradation	acetate
P161-PWY	acetylene degradation	H+
P161-PWY	acetylene degradation	NADH
P161-PWY	acetylene degradation	acetaldehyde
P161-PWY	acetylene degradation	ethanol
P161-PWY	acetylene degradation	NAD+
PWY0-1586	containing)	(1,4)-]-N-acetyl-α-D-muramoyl-L-alanyl-γ-D-glutamyl-meso-2,6-
PWY0-1586	containing)	D-alanine) tetrapeptide
PWY0-1586	containing)	H2O
PWY0-1586	containing)	D-alanine
PWY0-1586	containing)	glycine
PWY0-1586	containing)	diaminopimeloyl-D-alanyl-D-alanine) pentapeptide
PWY0-1586	containing)	diaminopimeloyl-D-alanyl-D-alanine) pentapeptide
PWY0-1586	containing)	diaminopimelate) tripeptide
PWY0-1586	containing)	diaminopimelate) tripeptide

PWY0-1586	containing)	containing)
PWY0-1586	containing)	containing)
PWY0-1586	containing)	containing)
PWY0-1586	containing)	containing)
PWY0-1586	containing)	diaminopimeloyl-D-alanine) tetrapeptide
PWY0-1586	containing)	diaminopimeloyl-D-alanine) tetrapeptide
PWY0-1586	containing)	di-trans,octa-cis-undecaprenyl diphosphate
PWY0-1586	containing)	H^+
PWY0-1586	containing)	diaminopimeloyl-glycine) tetrapeptide
PWY0-1586	containing)	diaminopimeloyl-glycine) tetrapeptide
PWY-5973	cis-vaccenate biosynthesis	cis-vaccenate
PWY-5973	cis-vaccenate biosynthesis	a soluble [acyl-carrier protein]
PWY-5973	cis-vaccenate biosynthesis	CO_2
PWY-5973	cis-vaccenate biosynthesis	a palmitoleoyl-[acp]
PWY-5973	cis-vaccenate biosynthesis	a malonyl-[acp]
PWY-5973	cis-vaccenate biosynthesis	NADPH
PWY-5973	cis-vaccenate biosynthesis	an (11Z)-3-oxooctadec-11-enoyl-[acp]
PWY-5973	cis-vaccenate biosynthesis	$NADP^+$
PWY-5973	cis-vaccenate biosynthesis	H_2O
PWY-5973	cis-vaccenate biosynthesis	a (3R,11Z)-3-hydroxyoctadec-11-enoyl-[acp]
PWY-5973	cis-vaccenate biosynthesis	H^+
PWY-5973	cis-vaccenate biosynthesis	NADH
PWY-5973	cis-vaccenate biosynthesis	a (2E,11Z)-octadeca-2,11-dienoyl-[acp]
PWY-5973	cis-vaccenate biosynthesis	a cis-vaccenoyl-[acp]
PWY-5973	cis-vaccenate biosynthesis	NAD^+
FOLSYN-PWY	and salvage	ADP
FOLSYN-PWY	and salvage	7,8-dihydrofolate monoglutamate
FOLSYN-PWY	and salvage	7,8-dihydropteroate
FOLSYN-PWY	and salvage	NADPH
FOLSYN-PWY	and salvage	a 7,8-dihydrofolate
FOLSYN-PWY	and salvage	$NADP^+$
FOLSYN-PWY	and salvage	AMP
FOLSYN-PWY	and salvage	(7,8-dihydropterin-6-yl)methyl diphosphate
FOLSYN-PWY	and salvage	ATP
FOLSYN-PWY	and salvage	6-(hydroxymethyl)-7,8-dihydropterin
FOLSYN-PWY	and salvage	glycolaldehyde
FOLSYN-PWY	and salvage	phosphate
FOLSYN-PWY	and salvage	D-erythro-7,8-dihydroneopterin
FOLSYN-PWY	and salvage	diphosphate
FOLSYN-PWY	and salvage	7,8-dihydroneopterin 3'-phosphate
FOLSYN-PWY	and salvage	7,8-dihydroneopterin 3'-triphosphate
FOLSYN-PWY	and salvage	formate
FOLSYN-PWY	and salvage	GTP
FOLSYN-PWY	and salvage	L-glutamate
FOLSYN-PWY	and salvage	chorismate
FOLSYN-PWY	and salvage	L-glutamine
FOLSYN-PWY	and salvage	pyruvate
FOLSYN-PWY	and salvage	4-aminobenzoate
FOLSYN-PWY	and salvage	4-amino-4-deoxychorismate
FOLSYN-PWY	and salvage	N2-formyl-N1-(5-phospho-β-D-ribosyl)glycinamide
FOLSYN-PWY	and salvage	a tetrahydrofolate
FOLSYN-PWY	and salvage	N1-(5-phospho-β-D-ribosyl)glycinamide
FOLSYN-PWY	and salvage	H^+
FOLSYN-PWY	and salvage	an N10-formyltetrahydrofolate
FOLSYN-PWY	and salvage	a 5,10-methenyltetrahydrofolate
FOLSYN-PWY	and salvage	H_2O
YNTH-PWY	glycogen biosynthesis I (from ADP-D-Glucose)	a phosphorylated phosphoglucomutase
YNTH-PWY	glycogen biosynthesis I (from ADP-D-Glucose)	α-glucose 1,6-bisphosphate
YNTH-PWY	glycogen biosynthesis I (from ADP-D-Glucose)	a phosphoglucomutase
YNTH-PWY	glycogen biosynthesis I (from ADP-D-Glucose)	ADP
YNTH-PWY	glycogen biosynthesis I (from ADP-D-Glucose)	a glycogen
YNTH-PWY	glycogen biosynthesis I (from ADP-D-Glucose)	a (1→4)-α-D-glucan
YNTH-PWY	glycogen biosynthesis I (from ADP-D-Glucose)	diphosphate
YNTH-PWY	glycogen biosynthesis I (from ADP-D-Glucose)	ADP-α-D-glucose
YNTH-PWY	glycogen biosynthesis I (from ADP-D-Glucose)	H^+
YNTH-PWY	glycogen biosynthesis I (from ADP-D-Glucose)	ATP
YNTH-PWY	glycogen biosynthesis I (from ADP-D-Glucose)	D-glucopyranose 6-phosphate
YNTH-PWY	glycogen biosynthesis I (from ADP-D-Glucose)	α-D-glucopyranose 1-phosphate
PWY0-1297	degradation	hypoxanthine
PWY0-1297	degradation	guanine
PWY0-1297	degradation	2'-deoxyguanosine
PWY0-1297	degradation	adenine

PWY0-1297	degradation	phosphate
PWY0-1297	degradation	2'-deoxyinosine
PWY0-1297	degradation	ammonium
PWY0-1297	degradation	H_2O
PWY0-1297	degradation	2'-deoxyadenosine
PWY0-1297	degradation	2-deoxy-α-D-ribose 1-phosphate
PWY0-1297	degradation	D-glyceraldehyde 3-phosphate
PWY0-1297	degradation	2-deoxy-D-ribose 5-phosphate
PWY0-1297	degradation	H+
PWY0-1297	degradation	NADH
PWY0-1297	degradation	acetyl-CoA
PWY0-1297	degradation	acetaldehyde
PWY0-1297	degradation	coenzyme A
PWY0-1297	degradation	NAD+
PWY-6467	Kdo transfer to lipid IVA III (Chlamydia)	lipid IVA (E. coli)
PWY-6467	Kdo transfer to lipid IVA III (Chlamydia)	α-Kdo-(2→6)-lipid IVA (E. coli)
PWY-6467	Kdo transfer to lipid IVA III (Chlamydia)	α-Kdo-(2->4)-α-Kdo-(2->4)-α-Kdo-(2->6)-lipid IVA
PWY-6467	Kdo transfer to lipid IVA III (Chlamydia)	α-Kdo-(2->4)-α-Kdo-(2->6)-lipid IVA (E. coli)
PWY-6467	Kdo transfer to lipid IVA III (Chlamydia)	H+
PWY-6467	Kdo transfer to lipid IVA III (Chlamydia)	CMP
PWY-6467	Kdo transfer to lipid IVA III (Chlamydia)	α-Kdo-(2->8)-[α-Kdo-(2->4)]-α-Kdo-(2->4)-α-Kdo-(2->6)-lipid IVA
PWY-6467	Kdo transfer to lipid IVA III (Chlamydia)	CMP-3-deoxy-β-D-manno-octulosonate
PWY-6467	Kdo transfer to lipid IVA III (Chlamydia)	α-Kdo-(2->8)-α-Kdo-(2->4)-α-Kdo-(2->6)-lipid IVA
PWY-7663	gondoate biosynthesis (anaerobic)	CO_2
PWY-7663	gondoate biosynthesis (anaerobic)	a soluble [acyl-carrier protein]
PWY-7663	gondoate biosynthesis (anaerobic)	an oleoyl-[acp]
PWY-7663	gondoate biosynthesis (anaerobic)	a malonyl-[acp]
PWY-7663	gondoate biosynthesis (anaerobic)	NADPH
PWY-7663	gondoate biosynthesis (anaerobic)	an (11Z)-3-oxo-icos-11-enoyl-[acp]
PWY-7663	gondoate biosynthesis (anaerobic)	NADP+
PWY-7663	gondoate biosynthesis (anaerobic)	H_2O
PWY-7663	gondoate biosynthesis (anaerobic)	a (3R,11Z)-3-hydroxy-icos-11-enoyl-[acp]
PWY-7663	gondoate biosynthesis (anaerobic)	H+
PWY-7663	gondoate biosynthesis (anaerobic)	NADH
PWY-7663	gondoate biosynthesis (anaerobic)	a (2E,11Z)-icosa-2,11-dienoyl-[acp]
PWY-7663	gondoate biosynthesis (anaerobic)	a gondoyl-[acp]
PWY-7663	gondoate biosynthesis (anaerobic)	NAD+
PWY-7332	derived O-antigen building blocks biosynthesis	a phosphorylated phosphoglucosamine mutase
PWY-7332	derived O-antigen building blocks biosynthesis	glucosamine 1,6-diphosphate
PWY-7332	derived O-antigen building blocks biosynthesis	a phosphoglucosamine mutase
PWY-7332	derived O-antigen building blocks biosynthesis	UDP-N-acetyl-β-L-pneumosamine
PWY-7332	derived O-antigen building blocks biosynthesis	UDP-2-acetamido-2,6-dideoxy-β-L-arabino-hex-4-ulose
PWY-7332	derived O-antigen building blocks biosynthesis	UDP-2-acetamido-2,6-dideoxy-β-L-lyxo-4-hexulose
PWY-7332	derived O-antigen building blocks biosynthesis	UDP-N-acetyl-β-L-quinovosamine
PWY-7332	derived O-antigen building blocks biosynthesis	UDP-N-acetyl-β-L-rhamnosamine
PWY-7332	derived O-antigen building blocks biosynthesis	UDP-N-acetyl-α-D-quinovosamine
PWY-7332	derived O-antigen building blocks biosynthesis	NAD(P)H
PWY-7332	derived O-antigen building blocks biosynthesis	UDP-2-acetamido-2,6-dideoxy-α-D-xylo-hex-4-ulose
PWY-7332	derived O-antigen building blocks biosynthesis	UDP-N-acetyl-α-D-fucosamine
PWY-7332	derived O-antigen building blocks biosynthesis	NAD(P)+
PWY-7332	derived O-antigen building blocks biosynthesis	UDP-N-acetyl-α-D-mannosaminuronate
PWY-7332	derived O-antigen building blocks biosynthesis	UDP-N-acetyl-α-D-mannosamine
PWY-7332	derived O-antigen building blocks biosynthesis	UDP-N-acetyl-α-D-galactosaminouronate
PWY-7332	derived O-antigen building blocks biosynthesis	UDP-2,3-diacetamido-2,3-dideoxy-α-D-glucuronate
PWY-7332	derived O-antigen building blocks biosynthesis	UDP-2-acetamido-3-amino-2,3-dideoxy-α-D-glucuronate
PWY-7332	derived O-antigen building blocks biosynthesis	2-oxoglutarate
PWY-7332	derived O-antigen building blocks biosynthesis	NADH
PWY-7332	derived O-antigen building blocks biosynthesis	UDP-2-acetamido-2-deoxy-α-D-ribo-hex-3-uluronate
PWY-7332	derived O-antigen building blocks biosynthesis	UDP-N-acetyl-α-D-glucosaminouronate
PWY-7332	derived O-antigen building blocks biosynthesis	NAD+
PWY-7332	derived O-antigen building blocks biosynthesis	UDP-2,6-dideoxy-2-acetamidino-β-L-galactose
PWY-7332	derived O-antigen building blocks biosynthesis	UDP-N-acetyl-β-L-fucosamine
PWY-7332	derived O-antigen building blocks biosynthesis	H_2O
PWY-7332	derived O-antigen building blocks biosynthesis	UDP-2-acetamido-3-acetamidino-2,3-dideoxy-α-D-mannuronate
PWY-7332	derived O-antigen building blocks biosynthesis	UDP-2,3-diacetamido-2,3-dideoxy-α-D-mannuronate
PWY-7332	derived O-antigen building blocks biosynthesis	ammonium
PWY-7332	derived O-antigen building blocks biosynthesis	diphosphate
PWY-7332	derived O-antigen building blocks biosynthesis	UDP-N-acetyl-α-D-glucosamine
PWY-7332	derived O-antigen building blocks biosynthesis	UTP
PWY-7332	derived O-antigen building blocks biosynthesis	H+
PWY-7332	derived O-antigen building blocks biosynthesis	coenzyme A
PWY-7332	derived O-antigen building blocks biosynthesis	N-acetyl-α-D-glucosamine 1-phosphate

PWY-7332	derived O-antigen building blocks biosynthesis	acetyl-CoA
PWY-7332	derived O-antigen building blocks biosynthesis	L-glutamate
PWY-7332	derived O-antigen building blocks biosynthesis	L-glutamine
PWY-7332	derived O-antigen building blocks biosynthesis	α-D-glucosamine 1-phosphate
PWY-7332	derived O-antigen building blocks biosynthesis	D-glucosamine 6-phosphate
PWY-7332	derived O-antigen building blocks biosynthesis	β-D-fructofuranose 6-phosphate
PWY-7332	derived O-antigen building blocks biosynthesis	D-glucopyranose 6-phosphate
PWY	lipid IVA biosynthesis	UDP
PWY	lipid IVA biosynthesis	ADP
PWY	lipid IVA biosynthesis	lipid IVA (E. coli)
PWY	lipid IVA biosynthesis	lipid A disaccharide (E. coli)
PWY	lipid IVA biosynthesis	ATP
PWY	lipid IVA biosynthesis	UMP
PWY	lipid IVA biosynthesis	lipid X (E. coli)
PWY	lipid IVA biosynthesis	H+
PWY	lipid IVA biosynthesis	UDP-2-N,3-O-bis[(3R)-3-hydroxytetradecanoyl]-α-D-glucosamine
PWY	lipid IVA biosynthesis	acetate
PWY	lipid IVA biosynthesis	UDP-3-O-(3-hydroxymyristoyl)-α-D-glucosamine
PWY	lipid IVA biosynthesis	H2O
PWY	lipid IVA biosynthesis	a soluble [acyl-carrier protein]
PWY	lipid IVA biosynthesis	UDP-3-O-[(3R)-3-hydroxymyristoyl]-N-acetyl-α-D-glucosamine
PWY	lipid IVA biosynthesis	UDP-N-acetyl-α-D-glucosamine
PWY	lipid IVA biosynthesis	a (3R)-3-hydroxytetradecanoyl-[acp]
PWY-1269	biosynthesis I	diphosphate
PWY-1269	biosynthesis I	CMP-3-deoxy-β-D-manno-octulosonate
PWY-1269	biosynthesis I	CTP
PWY-1269	biosynthesis I	3-deoxy-α-D-manno-2-octulosonate
PWY-1269	biosynthesis I	phosphate
PWY-1269	biosynthesis I	3-deoxy-D-manno-octulosonate 8-phosphate
PWY-1269	biosynthesis I	H2O
PWY-1269	biosynthesis I	phosphoenolpyruvate
PWY-1269	biosynthesis I	D-ribulose 5-phosphate
PWY-1269	biosynthesis I	D-arabinofuranose 5-phosphate
PWY-1269	biosynthesis I	aldehydo-D-arabinose 5-phosphate
PWY-7392	taxadiene biosynthesis (engineered)	geranyl diphosphate
PWY-7392	taxadiene biosynthesis (engineered)	CO2
PWY-7392	taxadiene biosynthesis (engineered)	pyruvate
PWY-7392	taxadiene biosynthesis (engineered)	D-glyceraldehyde 3-phosphate
PWY-7392	taxadiene biosynthesis (engineered)	NADPH
PWY-7392	taxadiene biosynthesis (engineered)	1-deoxy-D-xylulose 5-phosphate
PWY-7392	taxadiene biosynthesis (engineered)	NADP+
PWY-7392	taxadiene biosynthesis (engineered)	CMP
PWY-7392	taxadiene biosynthesis (engineered)	prenyl diphosphate
PWY-7392	taxadiene biosynthesis (engineered)	a reduced ferredoxin [iron-sulfur] cluster
PWY-7392	taxadiene biosynthesis (engineered)	an oxidized ferredoxin [iron-sulfur] cluster
PWY-7392	taxadiene biosynthesis (engineered)	ADP
PWY-7392	taxadiene biosynthesis (engineered)	2-phospho-4-(cytidine 5'-diphospho)-2-C-methyl-D-erythritol
PWY-7392	taxadiene biosynthesis (engineered)	ATP
PWY-7392	taxadiene biosynthesis (engineered)	4-(cytidine 5'-diphospho)-2-C-methyl-D-erythritol
PWY-7392	taxadiene biosynthesis (engineered)	2-C-methyl-D-erythritol 4-phosphate
PWY-7392	taxadiene biosynthesis (engineered)	CTP
PWY-7392	taxadiene biosynthesis (engineered)	a reduced flavodoxin
PWY-7392	taxadiene biosynthesis (engineered)	2-C-methyl-D-erythritol-2,4-cyclodiphosphate
PWY-7392	taxadiene biosynthesis (engineered)	(E)-4-hydroxy-3-methylbut-2-en-1-yl diphosphate
PWY-7392	taxadiene biosynthesis (engineered)	an oxidized flavodoxin
PWY-7392	taxadiene biosynthesis (engineered)	H2O
PWY-7392	taxadiene biosynthesis (engineered)	H+
PWY-7392	taxadiene biosynthesis (engineered)	(2E,6E)-farnesyl diphosphate
PWY-7392	taxadiene biosynthesis (engineered)	isopentenyl diphosphate
PWY-7392	taxadiene biosynthesis (engineered)	taxa-4,11-diene
PWY-7392	taxadiene biosynthesis (engineered)	diphosphate
PWY-7392	taxadiene biosynthesis (engineered)	geranylgeranyl diphosphate
PWY-7219	adenosine ribonucleotides de novo biosynthesis	GDP
PWY-7219	adenosine ribonucleotides de novo biosynthesis	phosphate
PWY-7219	adenosine ribonucleotides de novo biosynthesis	H+
PWY-7219	adenosine ribonucleotides de novo biosynthesis	L-aspartate
PWY-7219	adenosine ribonucleotides de novo biosynthesis	IMP
PWY-7219	adenosine ribonucleotides de novo biosynthesis	GTP
PWY-7219	adenosine ribonucleotides de novo biosynthesis	fumarate
PWY-7219	adenosine ribonucleotides de novo biosynthesis	adenylo-succinate
PWY-7219	adenosine ribonucleotides de novo biosynthesis	ADP
PWY-7219	adenosine ribonucleotides de novo biosynthesis	ATP

PWY-7219	adenosine ribonucleotides de novo biosynthesis	AMP
PWY	heme biosynthesis II (anaerobic)	Fe^{2+}
PWY	heme biosynthesis II (anaerobic)	protoheme
PWY	heme biosynthesis II (anaerobic)	uroporphyrinogen-III
PWY	heme biosynthesis II (anaerobic)	H^+
PWY	heme biosynthesis II (anaerobic)	5'-deoxyadenosine
PWY	heme biosynthesis II (anaerobic)	L-methionine
PWY	heme biosynthesis II (anaerobic)	CO_2
PWY	heme biosynthesis II (anaerobic)	S-adenosyl-L-methionine
PWY	heme biosynthesis II (anaerobic)	coproporphyrinogen III
PWY	heme biosynthesis II (anaerobic)	protoporphyrinogen IX
PWY	heme biosynthesis II (anaerobic)	a menaquinone
PWY	heme biosynthesis II (anaerobic)	a menaquinol
PWY	heme biosynthesis II (anaerobic)	protoporphyrin IX
PWY-2942	L-lysine biosynthesis III	ADP
PWY-2942	L-lysine biosynthesis III	L-aspartate
PWY-2942	L-lysine biosynthesis III	ATP
PWY-2942	L-lysine biosynthesis III	L-aspartyl-4-phosphate
PWY-2942	L-lysine biosynthesis III	phosphate
PWY-2942	L-lysine biosynthesis III	pyruvate
PWY-2942	L-lysine biosynthesis III	L-aspartate 4-semialdehyde
PWY-2942	L-lysine biosynthesis III	CO_2
PWY-2942	L-lysine biosynthesis III	L-lysine
PWY-2942	L-lysine biosynthesis III	NADPH
PWY-2942	L-lysine biosynthesis III	ammonium
PWY-2942	L-lysine biosynthesis III	L-α-amino-ε-keto-pimelate
PWY-2942	L-lysine biosynthesis III	meso-diaminopimelate
PWY-2942	L-lysine biosynthesis III	$NADP^+$
PWY-2942	L-lysine biosynthesis III	H^+
PWY-2942	L-lysine biosynthesis III	NAD(P)H
PWY-2942	L-lysine biosynthesis III	(2S,4S)-4-hydroxy-2,3,4,5-tetrahydrodipicolinate
PWY-2942	L-lysine biosynthesis III	(S)-2,3,4,5-tetrahydrodipicolinate
PWY-2942	L-lysine biosynthesis III	$NAD(P)^+$
PWY-2942	L-lysine biosynthesis III	H_2O
P124-PWY	Bifidobacterium shunt	acetate
P124-PWY	Bifidobacterium shunt	D-erythrose 4-phosphate
P124-PWY	Bifidobacterium shunt	D-sedoheptulose 7-phosphate
P124-PWY	Bifidobacterium shunt	D-ribose 5-phosphate
P124-PWY	Bifidobacterium shunt	D-ribulose 5-phosphate
P124-PWY	Bifidobacterium shunt	acetyl phosphate
P124-PWY	Bifidobacterium shunt	D-xylulose 5-phosphate
P124-PWY	Bifidobacterium shunt	D-glyceraldehyde 3-phosphate
P124-PWY	Bifidobacterium shunt	phosphate
P124-PWY	Bifidobacterium shunt	3-phospho-D-glyceroyl phosphate
P124-PWY	Bifidobacterium shunt	3-phospho-D-glycerate
P124-PWY	Bifidobacterium shunt	H_2O
P124-PWY	Bifidobacterium shunt	2-phospho-D-glycerate
P124-PWY	Bifidobacterium shunt	phosphoenolpyruvate
P124-PWY	Bifidobacterium shunt	NADH
P124-PWY	Bifidobacterium shunt	pyruvate
P124-PWY	Bifidobacterium shunt	(S)-lactate
P124-PWY	Bifidobacterium shunt	NAD^+
P124-PWY	Bifidobacterium shunt	β-D-fructofuranose 6-phosphate
P124-PWY	Bifidobacterium shunt	H^+
P124-PWY	Bifidobacterium shunt	ADP
P124-PWY	Bifidobacterium shunt	D-glucopyranose 6-phosphate
P124-PWY	Bifidobacterium shunt	D-glucopyranose
P124-PWY	Bifidobacterium shunt	ATP
PWY-6123	inosine-5'-phosphate biosynthesis I	L-aspartate
PWY-6123	inosine-5'-phosphate biosynthesis I	fumarate
PWY-6123	inosine-5'-phosphate biosynthesis I	5'-phosphoribosyl-4-(N-succinocarboxamide)-5-aminoimidazole
PWY-6123	inosine-5'-phosphate biosynthesis I	a tetrahydrofolate
PWY-6123	inosine-5'-phosphate biosynthesis I	an N10-formyltetrahydrofolate
PWY-6123	inosine-5'-phosphate biosynthesis I	5-amino-1-(5-phospho-D-ribosyl)imidazole-4-carboxamide
PWY-6123	inosine-5'-phosphate biosynthesis I	5-formamido-1-(5-phospho-D-ribosyl)-imidazole-4-carboxamide
PWY-6123	inosine-5'-phosphate biosynthesis I	IMP
PWY-6123	inosine-5'-phosphate biosynthesis I	H_2O
PWY-6123	inosine-5'-phosphate biosynthesis I	5-amino-1-(5-phospho-D-ribosyl)imidazole-4-carboxylate
PWY-6123	inosine-5'-phosphate biosynthesis I	phosphate
PWY-6123	inosine-5'-phosphate biosynthesis I	ADP
PWY-6123	inosine-5'-phosphate biosynthesis I	N5-carboxyaminoimidazole ribonucleotide
PWY-6123	inosine-5'-phosphate biosynthesis I	H^+

PWY-6123	inosine-5'-phosphate biosynthesis I	5-amino-1-(5-phospho-β-D-ribosyl)imidazole
PWY-6123	inosine-5'-phosphate biosynthesis I	ATP
PWY-6123	inosine-5'-phosphate biosynthesis I	hydrogencarbonate
PWY4FS-7	phosphatidylglycerol biosynthesis I (plastidic)	diphosphate
PWY4FS-7	phosphatidylglycerol biosynthesis I (plastidic)	CTP
PWY4FS-7	phosphatidylglycerol biosynthesis I (plastidic)	NAD(P)H
PWY4FS-7	phosphatidylglycerol biosynthesis I (plastidic)	glycerone phosphate
PWY4FS-7	phosphatidylglycerol biosynthesis I (plastidic)	NAD(P)+
PWY4FS-7	phosphatidylglycerol biosynthesis I (plastidic)	a 1,2-diacyl-sn-glycerol 3-phosphate
PWY4FS-7	phosphatidylglycerol biosynthesis I (plastidic)	a soluble [acyl-carrier protein]
PWY4FS-7	phosphatidylglycerol biosynthesis I (plastidic)	a 1-acyl-sn-glycerol 3-phosphate
PWY4FS-7	phosphatidylglycerol biosynthesis I (plastidic)	an acyl-[acyl-carrier protein]
PWY4FS-7	phosphatidylglycerol biosynthesis I (plastidic)	H+
PWY4FS-7	phosphatidylglycerol biosynthesis I (plastidic)	CMP
PWY4FS-7	phosphatidylglycerol biosynthesis I (plastidic)	a CDP-diacylglycerol
PWY4FS-7	phosphatidylglycerol biosynthesis I (plastidic)	sn-glycerol 3-phosphate
PWY4FS-7	phosphatidylglycerol biosynthesis I (plastidic)	phosphate
PWY4FS-7	phosphatidylglycerol biosynthesis I (plastidic)	an L-1-phosphatidyl-sn-glycerol
PWY4FS-7	phosphatidylglycerol biosynthesis I (plastidic)	1-(3-sn-phosphatidyl)-sn-glycerol 3-phosphate
PWY4FS-7	phosphatidylglycerol biosynthesis I (plastidic)	H2O
PWY4FS-8	phosphatidylglycerol biosynthesis II (non-plastidic)	diphosphate
PWY4FS-8	phosphatidylglycerol biosynthesis II (non-plastidic)	CTP
PWY4FS-8	phosphatidylglycerol biosynthesis II (non-plastidic)	a 1,2-diacyl-sn-glycerol 3-phosphate
PWY4FS-8	phosphatidylglycerol biosynthesis II (non-plastidic)	coenzyme A
PWY4FS-8	phosphatidylglycerol biosynthesis II (non-plastidic)	a 1-acyl-sn-glycerol 3-phosphate
PWY4FS-8	phosphatidylglycerol biosynthesis II (non-plastidic)	an acyl-CoA
PWY4FS-8	phosphatidylglycerol biosynthesis II (non-plastidic)	NAD(P)H
PWY4FS-8	phosphatidylglycerol biosynthesis II (non-plastidic)	glycerone phosphate
PWY4FS-8	phosphatidylglycerol biosynthesis II (non-plastidic)	NAD(P)+
PWY4FS-8	phosphatidylglycerol biosynthesis II (non-plastidic)	H+
PWY4FS-8	phosphatidylglycerol biosynthesis II (non-plastidic)	CMP
PWY4FS-8	phosphatidylglycerol biosynthesis II (non-plastidic)	a CDP-diacylglycerol
PWY4FS-8	phosphatidylglycerol biosynthesis II (non-plastidic)	sn-glycerol 3-phosphate
PWY4FS-8	phosphatidylglycerol biosynthesis II (non-plastidic)	phosphate
PWY4FS-8	phosphatidylglycerol biosynthesis II (non-plastidic)	an L-1-phosphatidyl-sn-glycerol
PWY4FS-8	phosphatidylglycerol biosynthesis II (non-plastidic)	1-(3-sn-phosphatidyl)-sn-glycerol 3-phosphate
PWY4FS-8	phosphatidylglycerol biosynthesis II (non-plastidic)	H2O
P42-PWY	incomplete reductive TCA cycle	E-
P42-PWY	incomplete reductive TCA cycle	acetyl-CoA
P42-PWY	incomplete reductive TCA cycle	pyruvate
P42-PWY	incomplete reductive TCA cycle	hydrogencarbonate
P42-PWY	incomplete reductive TCA cycle	H2O
P42-PWY	incomplete reductive TCA cycle	phosphate
P42-PWY	incomplete reductive TCA cycle	ADP
P42-PWY	incomplete reductive TCA cycle	ATP
P42-PWY	incomplete reductive TCA cycle	a reduced ferredoxin [iron-sulfur] cluster
P42-PWY	incomplete reductive TCA cycle	CO2
P42-PWY	incomplete reductive TCA cycle	succinyl-CoA
P42-PWY	incomplete reductive TCA cycle	2-oxoglutarate
P42-PWY	incomplete reductive TCA cycle	coenzyme A
P42-PWY	incomplete reductive TCA cycle	an oxidized ferredoxin [iron-sulfur] cluster
P42-PWY	incomplete reductive TCA cycle	H+
P42-PWY	incomplete reductive TCA cycle	NADH
P42-PWY	incomplete reductive TCA cycle	oxaloacetate
P42-PWY	incomplete reductive TCA cycle	(S)-malate
P42-PWY	incomplete reductive TCA cycle	NAD+
P42-PWY	incomplete reductive TCA cycle	succinate
P42-PWY	incomplete reductive TCA cycle	a menaquinone
P42-PWY	incomplete reductive TCA cycle	a menaquinol
P42-PWY	incomplete reductive TCA cycle	fumarate
PWY-5913	TCA cycle VI (obligate autotrophs)	oxalosuccinate
PWY-5913	TCA cycle VI (obligate autotrophs)	E-
PWY-5913	TCA cycle VI (obligate autotrophs)	acetyl-CoA
PWY-5913	TCA cycle VI (obligate autotrophs)	cis-aconitate
PWY-5913	TCA cycle VI (obligate autotrophs)	citrate
PWY-5913	TCA cycle VI (obligate autotrophs)	ammonium
PWY-5913	TCA cycle VI (obligate autotrophs)	H2O
PWY-5913	TCA cycle VI (obligate autotrophs)	hydrogencarbonate
PWY-5913	TCA cycle VI (obligate autotrophs)	phosphoenolpyruvate
PWY-5913	TCA cycle VI (obligate autotrophs)	L-glutamate
PWY-5913	TCA cycle VI (obligate autotrophs)	L-aspartate
PWY-5913	TCA cycle VI (obligate autotrophs)	phosphate

PWY-5913	TCA cycle VI (obligate autotrophs)	ADP
PWY-5913	TCA cycle VI (obligate autotrophs)	succinyl-CoA
PWY-5913	TCA cycle VI (obligate autotrophs)	coenzyme A
PWY-5913	TCA cycle VI (obligate autotrophs)	ATP
PWY-5913	TCA cycle VI (obligate autotrophs)	NADPH
PWY-5913	TCA cycle VI (obligate autotrophs)	CO2
PWY-5913	TCA cycle VI (obligate autotrophs)	2-oxoglutarate
PWY-5913	TCA cycle VI (obligate autotrophs)	D-threo-isocitrate
PWY-5913	TCA cycle VI (obligate autotrophs)	NADP+
PWY-5913	TCA cycle VI (obligate autotrophs)	H+
PWY-5913	TCA cycle VI (obligate autotrophs)	NADH
PWY-5913	TCA cycle VI (obligate autotrophs)	oxaloacetate
PWY-5913	TCA cycle VI (obligate autotrophs)	(S)-malate
PWY-5913	TCA cycle VI (obligate autotrophs)	NAD+
PWY-5913	TCA cycle VI (obligate autotrophs)	fumarate
PWY-5913	TCA cycle VI (obligate autotrophs)	an electron-transfer quinol
PWY-5913	TCA cycle VI (obligate autotrophs)	an electron-transfer quinone
PWY-5913	TCA cycle VI (obligate autotrophs)	succinate
SIS-PWY	glycolysis III (from glucose)	2,3-diphospho-D-glycerate
SIS-PWY	glycolysis III (from glucose)	a [protein]-L-histidine
SIS-PWY	glycolysis III (from glucose)	a [protein]-Nπ-phospho-L-histidine
SIS-PWY	glycolysis III (from glucose)	2/3-phospho-D-glycerate
SIS-PWY	glycolysis III (from glucose)	β-D-fructose 1,6-bisphosphate
SIS-PWY	glycolysis III (from glucose)	glycerone phosphate
SIS-PWY	glycolysis III (from glucose)	NADH
SIS-PWY	glycolysis III (from glucose)	D-glyceraldehyde 3-phosphate
SIS-PWY	glycolysis III (from glucose)	phosphate
SIS-PWY	glycolysis III (from glucose)	NAD+
SIS-PWY	glycolysis III (from glucose)	3-phospho-D-glyceroyl phosphate
SIS-PWY	glycolysis III (from glucose)	H2O
SIS-PWY	glycolysis III (from glucose)	phosphoenolpyruvate
SIS-PWY	glycolysis III (from glucose)	pyruvate
SIS-PWY	glycolysis III (from glucose)	β-D-fructofuranose 6-phosphate
SIS-PWY	glycolysis III (from glucose)	H+
SIS-PWY	glycolysis III (from glucose)	ADP
SIS-PWY	glycolysis III (from glucose)	D-glucopyranose 6-phosphate
SIS-PWY	glycolysis III (from glucose)	D-glucopyranose
SIS-PWY	glycolysis III (from glucose)	ATP
SIS-PWY	glycolysis III (from glucose)	3-phospho-D-glycerate
SIS-PWY	glycolysis III (from glucose)	2-phospho-D-glycerate
PWY-7539	biosynthesis III (Chlamydia)	AMP
PWY-7539	biosynthesis III (Chlamydia)	(7,8-dihydropterin-6-yl)methyl diphosphate
PWY-7539	biosynthesis III (Chlamydia)	ATP
PWY-7539	biosynthesis III (Chlamydia)	6-(hydroxymethyl)-7,8-dihydropterin
PWY-7539	biosynthesis III (Chlamydia)	glycolaldehyde
PWY-7539	biosynthesis III (Chlamydia)	phosphate
PWY-7539	biosynthesis III (Chlamydia)	D-erythro-7,8-dihydroneopterin
PWY-7539	biosynthesis III (Chlamydia)	formate
PWY-7539	biosynthesis III (Chlamydia)	diphosphate
PWY-7539	biosynthesis III (Chlamydia)	H+
PWY-7539	biosynthesis III (Chlamydia)	GTP
PWY-7539	biosynthesis III (Chlamydia)	H2O
PWY-7539	biosynthesis III (Chlamydia)	7,8-dihydroneopterin 3'-phosphate
PWY-7539	biosynthesis III (Chlamydia)	2,5-diamino-6-(5-phospho-D-ribosylamino)pyrimidin-4(3H)-one
PWY-6609	adenine and adenosine salvage III	α-D-ribose-1-phosphate
PWY-6609	adenine and adenosine salvage III	adenine
PWY-6609	adenine and adenosine salvage III	phosphate
PWY-6609	adenine and adenosine salvage III	ammonium
PWY-6609	adenine and adenosine salvage III	inosine
PWY-6609	adenine and adenosine salvage III	adenosine
PWY-6609	adenine and adenosine salvage III	H2O
PWY-6609	adenine and adenosine salvage III	H+
PWY-6609	adenine and adenosine salvage III	5-phospho-α-D-ribose 1-diphosphate
PWY-6609	adenine and adenosine salvage III	hypoxanthine
PWY-6609	adenine and adenosine salvage III	IMP
PWY-6609	adenine and adenosine salvage III	diphosphate
PWY-5695	degradation	IMP
PWY-5695	degradation	XMP
PWY-5695	degradation	α-D-ribose-1-phosphate
PWY-5695	degradation	xanthosine
PWY-5695	degradation	phosphate
PWY-5695	degradation	H+

PWY-5695	degradation	NADH
PWY-5695	degradation	urate
PWY-5695	degradation	xanthine
PWY-5695	degradation	NAD+
PWY-5695	degradation	H_2O
PWY-5667	CDP-diacylglycerol biosynthesis I	diphosphate
PWY-5667	CDP-diacylglycerol biosynthesis I	a CDP-diacylglycerol
PWY-5667	CDP-diacylglycerol biosynthesis I	CTP
PWY-5667	CDP-diacylglycerol biosynthesis I	a 1,2-diacyl-sn-glycerol 3-phosphate
PWY-5667	CDP-diacylglycerol biosynthesis I	coenzyme A
PWY-5667	CDP-diacylglycerol biosynthesis I	a 1-acyl-sn-glycerol 3-phosphate
PWY-5667	CDP-diacylglycerol biosynthesis I	an acyl-CoA
PWY-5667	CDP-diacylglycerol biosynthesis I	H+
PWY-5667	CDP-diacylglycerol biosynthesis I	NAD(P)H
PWY-5667	CDP-diacylglycerol biosynthesis I	glycerone phosphate
PWY-5667	CDP-diacylglycerol biosynthesis I	sn-glycerol 3-phosphate
PWY-5667	CDP-diacylglycerol biosynthesis I	NAD(P)+
PWY0-1319	CDP-diacylglycerol biosynthesis II	diphosphate
PWY0-1319	CDP-diacylglycerol biosynthesis II	a CDP-diacylglycerol
PWY0-1319	CDP-diacylglycerol biosynthesis II	CTP
PWY0-1319	CDP-diacylglycerol biosynthesis II	H+
PWY0-1319	CDP-diacylglycerol biosynthesis II	NAD(P)H
PWY0-1319	CDP-diacylglycerol biosynthesis II	glycerone phosphate
PWY0-1319	CDP-diacylglycerol biosynthesis II	NAD(P)+
PWY0-1319	CDP-diacylglycerol biosynthesis II	a 1,2-diacyl-sn-glycerol 3-phosphate
PWY0-1319	CDP-diacylglycerol biosynthesis II	a soluble [acyl-carrier protein]
PWY0-1319	CDP-diacylglycerol biosynthesis II	a 1-acyl-sn-glycerol 3-phosphate
PWY0-1319	CDP-diacylglycerol biosynthesis II	an acyl-[acyl-carrier protein]
PWY0-1319	CDP-diacylglycerol biosynthesis II	sn-glycerol 3-phosphate
P122-PWY	heterolactic fermentation	2,3-diphospho-D-glycerate
P122-PWY	heterolactic fermentation	a [protein]-L-histidine
P122-PWY	heterolactic fermentation	a [protein]-Nπ-phospho-L-histidine
P122-PWY	heterolactic fermentation	2/3-phospho-D-glycerate
P122-PWY	heterolactic fermentation	NAD(P)H
P122-PWY	heterolactic fermentation	CO_2
P122-PWY	heterolactic fermentation	NAD(P)+
P122-PWY	heterolactic fermentation	D-ribulose 5-phosphate
P122-PWY	heterolactic fermentation	D-xylulose 5-phosphate
P122-PWY	heterolactic fermentation	D-glyceraldehyde 3-phosphate
P122-PWY	heterolactic fermentation	3-phospho-D-glyceroyl phosphate
P122-PWY	heterolactic fermentation	3-phospho-D-glycerate
P122-PWY	heterolactic fermentation	2-phospho-D-glycerate
P122-PWY	heterolactic fermentation	phosphoenolpyruvate
P122-PWY	heterolactic fermentation	acetyl phosphate
P122-PWY	heterolactic fermentation	phosphate
P122-PWY	heterolactic fermentation	acetyl-CoA
P122-PWY	heterolactic fermentation	coenzyme A
P122-PWY	heterolactic fermentation	acetaldehyde
P122-PWY	heterolactic fermentation	ethanol
P122-PWY	heterolactic fermentation	D-gluconate 6-phosphate
P122-PWY	heterolactic fermentation	H_2O
P122-PWY	heterolactic fermentation	(S)-lactate
P122-PWY	heterolactic fermentation	NADH
P122-PWY	heterolactic fermentation	pyruvate
P122-PWY	heterolactic fermentation	(R)-lactate
P122-PWY	heterolactic fermentation	NAD+
P122-PWY	heterolactic fermentation	β-D-fructofuranose
P122-PWY	heterolactic fermentation	β-D-fructofuranose 6-phosphate
P122-PWY	heterolactic fermentation	NADPH
P122-PWY	heterolactic fermentation	6-phospho D-glucono-1,5-lactone
P122-PWY	heterolactic fermentation	NADP+
P122-PWY	heterolactic fermentation	H+
P122-PWY	heterolactic fermentation	ADP
P122-PWY	heterolactic fermentation	D-glucopyranose 6-phosphate
P122-PWY	heterolactic fermentation	D-glucopyranose
P122-PWY	heterolactic fermentation	ATP
PWY-2941	L-lysine biosynthesis II	ADP
PWY-2941	L-lysine biosynthesis II	L-aspartate
PWY-2941	L-lysine biosynthesis II	ATP
PWY-2941	L-lysine biosynthesis II	NADPH
PWY-2941	L-lysine biosynthesis II	L-aspartyl-4-phosphate
PWY-2941	L-lysine biosynthesis II	phosphate

PWY-2941	L-lysine biosynthesis II	NADP+
PWY-2941	L-lysine biosynthesis II	pyruvate
PWY-2941	L-lysine biosynthesis II	L-aspartate 4-semialdehyde
PWY-2941	L-lysine biosynthesis II	coenzyme A
PWY-2941	L-lysine biosynthesis II	acetyl-CoA
PWY-2941	L-lysine biosynthesis II	2-oxoglutarate
PWY-2941	L-lysine biosynthesis II	L-2-acetamido-6-oxoheptanedioate
PWY-2941	L-lysine biosynthesis II	L-glutamate
PWY-2941	L-lysine biosynthesis II	acetate
PWY-2941	L-lysine biosynthesis II	N-acetyl-L,L-2,6-diaminopimelate
PWY-2941	L-lysine biosynthesis II	CO_2
PWY-2941	L-lysine biosynthesis II	L-lysine
PWY-2941	L-lysine biosynthesis II	meso-diaminopimelate
PWY-2941	L-lysine biosynthesis II	L,L-diaminopimelate
PWY-2941	L-lysine biosynthesis II	H+
PWY-2941	L-lysine biosynthesis II	NAD(P)H
PWY-2941	L-lysine biosynthesis II	(2S,4S)-4-hydroxy-2,3,4,5-tetrahydrodipicolinate
PWY-2941	L-lysine biosynthesis II	(S)-2,3,4,5-tetrahydrodipicolinate
PWY-2941	L-lysine biosynthesis II	NAD(P)+
PWY-2941	L-lysine biosynthesis II	H_2O
PWY-5097	L-lysine biosynthesis VI	ADP
PWY-5097	L-lysine biosynthesis VI	L-aspartate
PWY-5097	L-lysine biosynthesis VI	ATP
PWY-5097	L-lysine biosynthesis VI	NADPH
PWY-5097	L-lysine biosynthesis VI	L-aspartyl-4-phosphate
PWY-5097	L-lysine biosynthesis VI	phosphate
PWY-5097	L-lysine biosynthesis VI	NADP+
PWY-5097	L-lysine biosynthesis VI	pyruvate
PWY-5097	L-lysine biosynthesis VI	L-aspartate 4-semialdehyde
PWY-5097	L-lysine biosynthesis VI	L-glutamate
PWY-5097	L-lysine biosynthesis VI	2-oxoglutarate
PWY-5097	L-lysine biosynthesis VI	L,L-diaminopimelate
PWY-5097	L-lysine biosynthesis VI	CO_2
PWY-5097	L-lysine biosynthesis VI	L-lysine
PWY-5097	L-lysine biosynthesis VI	meso-diaminopimelate
PWY-5097	L-lysine biosynthesis VI	H+
PWY-5097	L-lysine biosynthesis VI	NAD(P)H
PWY-5097	L-lysine biosynthesis VI	(2S,4S)-4-hydroxy-2,3,4,5-tetrahydrodipicolinate
PWY-5097	L-lysine biosynthesis VI	(S)-2,3,4,5-tetrahydrodipicolinate
PWY-5097	L-lysine biosynthesis VI	NAD(P)+
PWY-5097	L-lysine biosynthesis VI	H_2O
PWY-7323	antigen building blocks biosynthesis	D-mannopyranose 6-phosphate
PWY-7323	antigen building blocks biosynthesis	diphosphate
PWY-7323	antigen building blocks biosynthesis	α-D-mannose 1-phosphate
PWY-7323	antigen building blocks biosynthesis	GTP
PWY-7323	antigen building blocks biosynthesis	β-D-fructofuranose 6-phosphate
PWY-7323	antigen building blocks biosynthesis	D-glucopyranose 6-phosphate
PWY-7323	antigen building blocks biosynthesis	GDP-α-D-mannose
PWY-7323	antigen building blocks biosynthesis	NADPH
PWY-7323	antigen building blocks biosynthesis	GDP-4-dehydro-6-deoxy-β-L-galactose
PWY-7323	antigen building blocks biosynthesis	GDP-β-L-fucose
PWY-7323	antigen building blocks biosynthesis	NADP+
PWY-7323	antigen building blocks biosynthesis	GDP-β-colitose
PWY-7323	antigen building blocks biosynthesis	ammonium
PWY-7323	antigen building blocks biosynthesis	GDP-4-dehydro-3,6-dideoxy-α-D-mannose
PWY-7323	antigen building blocks biosynthesis	H_2O
PWY-7323	antigen building blocks biosynthesis	2-GDP-[(2S,3S,6R)-5-imino-6-methyloxan-3-ol]
PWY-7323	antigen building blocks biosynthesis	2-GDP-[(2S,3S,6R)-5-amino-6-methyl-3,6-dihydro-2H-pyran-3-ol]
PWY-7323	antigen building blocks biosynthesis	L-glutamate
PWY-7323	antigen building blocks biosynthesis	GDP-α-D-perosamine
PWY-7323	antigen building blocks biosynthesis	2-oxoglutarate
PWY-7323	antigen building blocks biosynthesis	GDP-α-D-rhamnose
PWY-7323	antigen building blocks biosynthesis	H+
PWY-7323	antigen building blocks biosynthesis	NAD(P)H
PWY-7323	antigen building blocks biosynthesis	GDP-4-dehydro-α-D-rhamnose
PWY-7323	antigen building blocks biosynthesis	GDP-6-deoxy-α-D-talose
PWY-7323	antigen building blocks biosynthesis	NAD(P)+
PWY-5910	biosynthesis I (via mevalonate)	a [protein]-L-cysteine
PWY-5910	biosynthesis I (via mevalonate)	a [protein] S-acetyl-L-cysteine
PWY-5910	biosynthesis I (via mevalonate)	acetoacetyl-CoA
PWY-5910	biosynthesis I (via mevalonate)	H_2O
PWY-5910	biosynthesis I (via mevalonate)	acetyl-CoA

PWY-5910	biosynthesis I (via mevalonate)	(S)-3-hydroxy-3-methylglutaryl-CoA
PWY-5910	biosynthesis I (via mevalonate)	NADPH
PWY-5910	biosynthesis I (via mevalonate)	NADP+
PWY-5910	biosynthesis I (via mevalonate)	coenzyme A
PWY-5910	biosynthesis I (via mevalonate)	H+
PWY-5910	biosynthesis I (via mevalonate)	(R)-mevalonate
PWY-5910	biosynthesis I (via mevalonate)	(R)-5-phosphomevalonate
PWY-5910	biosynthesis I (via mevalonate)	phosphate
PWY-5910	biosynthesis I (via mevalonate)	ADP
PWY-5910	biosynthesis I (via mevalonate)	CO2
PWY-5910	biosynthesis I (via mevalonate)	(R)-mevalonate diphosphate
PWY-5910	biosynthesis I (via mevalonate)	ATP
PWY-5910	biosynthesis I (via mevalonate)	prenyl diphosphate
PWY-5910	biosynthesis I (via mevalonate)	geranyl diphosphate
PWY-5910	biosynthesis I (via mevalonate)	diphosphate
PWY-5910	biosynthesis I (via mevalonate)	geranylgeranyl diphosphate
PWY-5910	biosynthesis I (via mevalonate)	(2E,6E)-farnesyl diphosphate
PWY-5910	biosynthesis I (via mevalonate)	isopentenyl diphosphate
PWY-922	mevalonate pathway I	a [protein]-L-cysteine
PWY-922	mevalonate pathway I	a [protein] S-acetyl-L-cysteine
PWY-922	mevalonate pathway I	acetoacetyl-CoA
PWY-922	mevalonate pathway I	H2O
PWY-922	mevalonate pathway I	acetyl-CoA
PWY-922	mevalonate pathway I	(S)-3-hydroxy-3-methylglutaryl-CoA
PWY-922	mevalonate pathway I	NADPH
PWY-922	mevalonate pathway I	NADP+
PWY-922	mevalonate pathway I	coenzyme A
PWY-922	mevalonate pathway I	H+
PWY-922	mevalonate pathway I	(R)-mevalonate
PWY-922	mevalonate pathway I	(R)-5-phosphomevalonate
PWY-922	mevalonate pathway I	phosphate
PWY-922	mevalonate pathway I	ADP
PWY-922	mevalonate pathway I	CO2
PWY-922	mevalonate pathway I	(R)-mevalonate diphosphate
PWY-922	mevalonate pathway I	ATP
PWY-922	mevalonate pathway I	prenyl diphosphate
PWY-922	mevalonate pathway I	isopentenyl diphosphate
BIOSYNTHESI	heme biosynthesis I (aerobic)	uroporphyrinogen-III
BIOSYNTHESI	heme biosynthesis I (aerobic)	hydrogen peroxide
BIOSYNTHESI	heme biosynthesis I (aerobic)	Fe2+
BIOSYNTHESI	heme biosynthesis I (aerobic)	protoporphyrin IX
BIOSYNTHESI	heme biosynthesis I (aerobic)	protoheme
BIOSYNTHESI	heme biosynthesis I (aerobic)	H2O
BIOSYNTHESI	heme biosynthesis I (aerobic)	CO2
BIOSYNTHESI	heme biosynthesis I (aerobic)	protoporphyrinogen IX
BIOSYNTHESI	heme biosynthesis I (aerobic)	coproporphyrinogen III
BIOSYNTHESI	heme biosynthesis I (aerobic)	oxygen
BIOSYNTHESI	heme biosynthesis I (aerobic)	H+
CAT-PWY	homolactic fermentation	2,3-diphospho-D-glycerate
CAT-PWY	homolactic fermentation	a [protein]-L-histidine
CAT-PWY	homolactic fermentation	a [protein]-Nπ-phospho-L-histidine
CAT-PWY	homolactic fermentation	2/3-phospho-D-glycerate
CAT-PWY	homolactic fermentation	(S)-lactate
CAT-PWY	homolactic fermentation	β-D-fructose 1,6-bisphosphate
CAT-PWY	homolactic fermentation	glycerone phosphate
CAT-PWY	homolactic fermentation	NADH
CAT-PWY	homolactic fermentation	D-glyceraldehyde 3-phosphate
CAT-PWY	homolactic fermentation	phosphate
CAT-PWY	homolactic fermentation	NAD+
CAT-PWY	homolactic fermentation	3-phospho-D-glyceroyl phosphate
CAT-PWY	homolactic fermentation	H2O
CAT-PWY	homolactic fermentation	phosphoenolpyruvate
CAT-PWY	homolactic fermentation	pyruvate
CAT-PWY	homolactic fermentation	β-D-fructofuranose 6-phosphate
CAT-PWY	homolactic fermentation	H+
CAT-PWY	homolactic fermentation	ADP
CAT-PWY	homolactic fermentation	D-glucopyranose 6-phosphate
CAT-PWY	homolactic fermentation	D-glucopyranose
CAT-PWY	homolactic fermentation	ATP
CAT-PWY	homolactic fermentation	3-phospho-D-glycerate
CAT-PWY	homolactic fermentation	2-phospho-D-glycerate
PWY-7234	inosine-5'-phosphate biosynthesis III	L-aspartate

PWY-7234	inosine-5'-phosphate biosynthesis III	fumarate
PWY-7234	inosine-5'-phosphate biosynthesis III	5'-phosphoribosyl-4-(N-succinocarboxamide)-5-aminoimidazole
PWY-7234	inosine-5'-phosphate biosynthesis III	IMP
PWY-7234	inosine-5'-phosphate biosynthesis III	H2O
PWY-7234	inosine-5'-phosphate biosynthesis III	5-amino-1-(5-phospho-D-ribosyl)imidazole-4-carboxylate
PWY-7234	inosine-5'-phosphate biosynthesis III	N5-carboxyaminoimidazole ribonucleotide
PWY-7234	inosine-5'-phosphate biosynthesis III	H+
PWY-7234	inosine-5'-phosphate biosynthesis III	5-amino-1-(5-phospho-β-D-ribosyl)imidazole
PWY-7234	inosine-5'-phosphate biosynthesis III	hydrogencarbonate
PWY-7234	inosine-5'-phosphate biosynthesis III	phosphate
PWY-7234	inosine-5'-phosphate biosynthesis III	ADP
PWY-7234	inosine-5'-phosphate biosynthesis III	5-formamido-1-(5-phospho-D-ribosyl)-imidazole-4-carboxamide
PWY-7234	inosine-5'-phosphate biosynthesis III	5-amino-1-(5-phospho-D-ribosyl)imidazole-4-carboxamide
PWY-7234	inosine-5'-phosphate biosynthesis III	formate
PWY-7234	inosine-5'-phosphate biosynthesis III	ATP
PWY-7200	salvage	dTTP
PWY-7200	salvage	dTDP
PWY-7200	salvage	dCTP
PWY-7200	salvage	dCDP
PWY-7200	salvage	ammonium
PWY-7200	salvage	H2O
PWY-7200	salvage	2'-deoxyuridine
PWY-7200	salvage	ADP
PWY-7200	salvage	thymidine
PWY-7200	salvage	ATP
PWY-7200	salvage	H+
PWY-7200	salvage	a nucleoside diphosphate
PWY-7200	salvage	dCMP
PWY-7200	salvage	2'-deoxycytidine
PWY-7200	salvage	a nucleoside triphosphate
PWY-7200	salvage	a 7,8-dihydrofolate
PWY-7200	salvage	dTMP
PWY-7200	salvage	a 5,10-methylenetetrahydrofolate
PWY-7200	salvage	dUMP
PWY	gluconeogenesis I	β-D-fructose 1,6-bisphosphate
PWY	gluconeogenesis I	3-phospho-D-glyceroyl phosphate
PWY	gluconeogenesis I	3-phospho-D-glycerate
PWY	gluconeogenesis I	2-phospho-D-glycerate
PWY	gluconeogenesis I	ADP
PWY	gluconeogenesis I	AMP
PWY	gluconeogenesis I	phosphoenolpyruvate
PWY	gluconeogenesis I	phosphate
PWY	gluconeogenesis I	H2O
PWY	gluconeogenesis I	ATP
PWY	gluconeogenesis I	NADPH
PWY	gluconeogenesis I	NADP+
PWY	gluconeogenesis I	glycerone phosphate
PWY	gluconeogenesis I	D-glyceraldehyde 3-phosphate
PWY	gluconeogenesis I	CO2
PWY	gluconeogenesis I	pyruvate
PWY	gluconeogenesis I	H+
PWY	gluconeogenesis I	NADH
PWY	gluconeogenesis I	oxaloacetate
PWY	gluconeogenesis I	(S)-malate
PWY	gluconeogenesis I	NAD+
PWY	gluconeogenesis I	β-D-fructofuranose 6-phosphate
PWY	gluconeogenesis I	D-glucopyranose 6-phosphate
PWY-5686	UMP biosynthesis	ammonium
PWY-5686	UMP biosynthesis	carbamate
PWY-5686	UMP biosynthesis	carboxyphosphate
PWY-5686	UMP biosynthesis	E-
PWY-5686	UMP biosynthesis	ADP
PWY-5686	UMP biosynthesis	L-glutamate
PWY-5686	UMP biosynthesis	ATP
PWY-5686	UMP biosynthesis	L-glutamine
PWY-5686	UMP biosynthesis	hydrogencarbonate
PWY-5686	UMP biosynthesis	phosphate
PWY-5686	UMP biosynthesis	L-aspartate
PWY-5686	UMP biosynthesis	carbamoyl phosphate
PWY-5686	UMP biosynthesis	N-carbamoyl-L-aspartate
PWY-5686	UMP biosynthesis	H2O
PWY-5686	UMP biosynthesis	5-phospho-α-D-ribose 1-diphosphate

PWY-5686	UMP biosynthesis	diphosphate
PWY-5686	UMP biosynthesis	UMP
PWY-5686	UMP biosynthesis	CO_2
PWY-5686	UMP biosynthesis	H+
PWY-5686	UMP biosynthesis	orotidine 5'-phosphate
PWY-5686	UMP biosynthesis	orotate
PWY-5686	UMP biosynthesis	an electron-transfer quinol
PWY-5686	UMP biosynthesis	an electron-transfer quinone
PWY-5686	UMP biosynthesis	(S)-dihydroorotate
PWY	lactose and galactose degradation I	ADP
PWY	lactose and galactose degradation I	H+
PWY	lactose and galactose degradation I	ATP
PWY	lactose and galactose degradation I	D-glucopyranose
PWY	lactose and galactose degradation I	lactose 6'-phosphate
PWY	lactose and galactose degradation I	H_2O
PWY	lactose and galactose degradation I	D-tagatofuranose 6-phosphate
PWY	lactose and galactose degradation I	D-galactopyranose 6-phosphate
PWY	lactose and galactose degradation I	D-glyceraldehyde 3-phosphate
PWY	lactose and galactose degradation I	glycerone phosphate
PWY	lactose and galactose degradation I	D-tagatofuranose 1,6-bisphosphate
PWY-6608	guanosine nucleotides degradation III	ammonium
PWY-6608	guanosine nucleotides degradation III	GMP
PWY-6608	guanosine nucleotides degradation III	H+
PWY-6608	guanosine nucleotides degradation III	NADH
PWY-6608	guanosine nucleotides degradation III	urate
PWY-6608	guanosine nucleotides degradation III	xanthine
PWY-6608	guanosine nucleotides degradation III	NAD+
PWY-6608	guanosine nucleotides degradation III	H_2O
PWY-6608	guanosine nucleotides degradation III	α-D-ribose-1-phosphate
PWY-6608	guanosine nucleotides degradation III	guanine
PWY-6608	guanosine nucleotides degradation III	guanosine
PWY-6608	guanosine nucleotides degradation III	phosphate
PWY	(bacteria)	diphosphate
PWY	(bacteria)	CTP
PWY	(bacteria)	coenzyme A
PWY	(bacteria)	an acyl-CoA
PWY	(bacteria)	NAD(P)H
PWY	(bacteria)	glycerone phosphate
PWY	(bacteria)	NAD(P)+
PWY	(bacteria)	phosphate
PWY	(bacteria)	H_2O
PWY	(bacteria)	glycerol
PWY	(bacteria)	1-(3-sn-phosphatidyl)-sn-glycerol 3-phosphate
PWY	(bacteria)	CMP
PWY	(bacteria)	a CDP-diacylglycerol
PWY	(bacteria)	L-serine
PWY	(bacteria)	CO_2
PWY	(bacteria)	a 3-sn-phosphatidyl-L-serine
PWY	(bacteria)	H+
PWY	(bacteria)	a 1,2-diacyl-sn-glycerol 3-phosphate
PWY	(bacteria)	a soluble [acyl-carrier protein]
PWY	(bacteria)	a 1-acyl-sn-glycerol 3-phosphate
PWY	(bacteria)	an acyl-[acyl-carrier protein]
PWY	(bacteria)	sn-glycerol 3-phosphate
PWY	(bacteria)	ethanolamine
PWY	(bacteria)	a cardiolipin
PWY	(bacteria)	an L-1-phosphatidyl-sn-glycerol
PWY	(bacteria)	an L-1-phosphatidylethanolamine
D-PWY	teichoic acid (poly-glycerol) biosynthesis	(D-alanyl)adenylate
D-PWY	teichoic acid (poly-glycerol) biosynthesis	UDP-N-acetyl-α-D-mannosamine
D-PWY	teichoic acid (poly-glycerol) biosynthesis	ManNAc-GlcNAc-PP-undecaprenol
D-PWY	teichoic acid (poly-glycerol) biosynthesis	UDP
D-PWY	teichoic acid (poly-glycerol) biosynthesis	UDP-α-D-glucose
D-PWY	teichoic acid (poly-glycerol) biosynthesis	di-trans,octa-cis-undecaprenyl diphosphate
D-PWY	teichoic acid (poly-glycerol) biosynthesis	a peptidoglycan-major wall teichoic acid complex (B. subtilis 168)
D-PWY	teichoic acid (poly-glycerol) biosynthesis	(1,4)-]-N-acetyl-α-D-muramoyl-L-alanyl-γ-D-glutamyl-meso-2,6-
D-PWY	teichoic acid (poly-glycerol) biosynthesis	CMP
D-PWY	teichoic acid (poly-glycerol) biosynthesis	(Gro-P)n-Gro-P-ManNAc-GlcNAc-PP-undecaprenol
D-PWY	teichoic acid (poly-glycerol) biosynthesis	Gro-P-ManNAc-GlcNAc-PP-undecaprenol
D-PWY	teichoic acid (poly-glycerol) biosynthesis	CDP-glycerol
D-PWY	teichoic acid (poly-glycerol) biosynthesis	sn-glycerol 3-phosphate
D-PWY	teichoic acid (poly-glycerol) biosynthesis	CTP

D-PWY	teichoic acid (poly-glycerol) biosynthesis	UMP
D-PWY	teichoic acid (poly-glycerol) biosynthesis	N-acetyl-α-D-glucosaminyldiphospho-ditrans,octacis-undecaprenol
D-PWY	teichoic acid (poly-glycerol) biosynthesis	UDP-N-acetyl-α-D-glucosamine
D-PWY	teichoic acid (poly-glycerol) biosynthesis	di-trans,octa-cis-undecaprenyl phosphate
D-PWY	teichoic acid (poly-glycerol) biosynthesis	a polyisoprenyl-major wall teichoic acid (B. subtilis 168)
D-PWY	teichoic acid (poly-glycerol) biosynthesis	H+
D-PWY	teichoic acid (poly-glycerol) biosynthesis	phosphate
D-PWY	teichoic acid (poly-glycerol) biosynthesis	ADP
D-PWY	teichoic acid (poly-glycerol) biosynthesis	[(2-Glc)-Gro-P]n-Gro-P-ManNAc-GlcNAc-PP-undecaprenol
D-PWY	teichoic acid (poly-glycerol) biosynthesis	H2O
D-PWY	teichoic acid (poly-glycerol) biosynthesis	diphosphate
D-PWY	teichoic acid (poly-glycerol) biosynthesis	AMP
D-PWY	teichoic acid (poly-glycerol) biosynthesis	ATP
D-PWY	teichoic acid (poly-glycerol) biosynthesis	D-alanine
D-PWY	teichoic acid (poly-glycerol) biosynthesis	a holo [D-alanyl carrier protein]
D-PWY	teichoic acid (poly-glycerol) biosynthesis	2-O-D-alanyl-1-O-phosphatidylglycerol
D-PWY	teichoic acid (poly-glycerol) biosynthesis	a D-alanyl-[D-alanyl carrier protein]
D-PWY	teichoic acid (poly-glycerol) biosynthesis	an L-1-phosphatidyl-sn-glycerol
1CMET2-PWY	N10-formyl-tetrahydrofolate biosynthesis	a [glycine-cleavage complex H protein] N6-lipoyl-L-lysine
1CMET2-PWY	N10-formyl-tetrahydrofolate biosynthesis	lysine
1CMET2-PWY	N10-formyl-tetrahydrofolate biosynthesis	a [glycine-cleavage complex H protein] N6-dihydrolipoyl-L-lysine
1CMET2-PWY	N10-formyl-tetrahydrofolate biosynthesis	phosphate
1CMET2-PWY	N10-formyl-tetrahydrofolate biosynthesis	ADP
1CMET2-PWY	N10-formyl-tetrahydrofolate biosynthesis	a (6S)-5-formyltetrahydrofolate
1CMET2-PWY	N10-formyl-tetrahydrofolate biosynthesis	ATP
1CMET2-PWY	N10-formyl-tetrahydrofolate biosynthesis	L-methionine
1CMET2-PWY	N10-formyl-tetrahydrofolate biosynthesis	L-homocysteine
1CMET2-PWY	N10-formyl-tetrahydrofolate biosynthesis	a 5-methyltetrahydrofolate
1CMET2-PWY	N10-formyl-tetrahydrofolate biosynthesis	NADH
1CMET2-PWY	N10-formyl-tetrahydrofolate biosynthesis	CO2
1CMET2-PWY	N10-formyl-tetrahydrofolate biosynthesis	ammonium
1CMET2-PWY	N10-formyl-tetrahydrofolate biosynthesis	NAD+
1CMET2-PWY	N10-formyl-tetrahydrofolate biosynthesis	H+
1CMET2-PWY	N10-formyl-tetrahydrofolate biosynthesis	an N10-formyltetrahydrofolate
1CMET2-PWY	N10-formyl-tetrahydrofolate biosynthesis	NADPH
1CMET2-PWY	N10-formyl-tetrahydrofolate biosynthesis	a 5,10-methenyltetrahydrofolate
1CMET2-PWY	N10-formyl-tetrahydrofolate biosynthesis	NADP+
1CMET2-PWY	N10-formyl-tetrahydrofolate biosynthesis	H2O
1CMET2-PWY	N10-formyl-tetrahydrofolate biosynthesis	glycine
1CMET2-PWY	N10-formyl-tetrahydrofolate biosynthesis	L-serine
1CMET2-PWY	N10-formyl-tetrahydrofolate biosynthesis	a tetrahydrofolate
1CMET2-PWY	N10-formyl-tetrahydrofolate biosynthesis	a 7,8-dihydrofolate
1CMET2-PWY	N10-formyl-tetrahydrofolate biosynthesis	dTMP
1CMET2-PWY	N10-formyl-tetrahydrofolate biosynthesis	a 5,10-methylenetetrahydrofolate
1CMET2-PWY	N10-formyl-tetrahydrofolate biosynthesis	dUMP
PWY-5180	toluene degradation I (aerobic) (via o-cresol)	acetate
PWY-5180	toluene degradation I (aerobic) (via o-cresol)	(2Z,4E)-2-hydroxy-6-oxohepta-2,4-dienoate
PWY-5180	toluene degradation I (aerobic) (via o-cresol)	3-methylcatechol
PWY-5180	toluene degradation I (aerobic) (via o-cresol)	2-methylphenol
PWY-5180	toluene degradation I (aerobic) (via o-cresol)	toluene
PWY-5180	toluene degradation I (aerobic) (via o-cresol)	oxygen
PWY-5180	toluene degradation I (aerobic) (via o-cresol)	H2O
PWY-5180	toluene degradation I (aerobic) (via o-cresol)	(2Z)-2-hydroxypenta-2,4-dienoate
PWY-5180	toluene degradation I (aerobic) (via o-cresol)	pyruvate
PWY-5180	toluene degradation I (aerobic) (via o-cresol)	(S)-4-hydroxy-2-oxopentanoate
PWY-5180	toluene degradation I (aerobic) (via o-cresol)	H+
PWY-5180	toluene degradation I (aerobic) (via o-cresol)	NADH
PWY-5180	toluene degradation I (aerobic) (via o-cresol)	acetyl-CoA
PWY-5180	toluene degradation I (aerobic) (via o-cresol)	acetaldehyde
PWY-5180	toluene degradation I (aerobic) (via o-cresol)	coenzyme A
PWY-5180	toluene degradation I (aerobic) (via o-cresol)	NAD+
PWY-5182	methylcatechol)	4-methylphenol
PWY-5182	methylcatechol)	toluene
PWY-5182	methylcatechol)	4-methylcatechol
PWY-5182	methylcatechol)	oxygen
PWY-5182	methylcatechol)	formate
PWY-5182	methylcatechol)	(2Z,4E)-2-hydroxy-5-methyl-6-oxohexa-2,4-dienoate
PWY-5182	methylcatechol)	(2Z)-2-hydroxyhexa-2,5-dienoate
PWY-5182	methylcatechol)	1-propanal
PWY-5182	methylcatechol)	(S)-4-hydroxy-2-oxohexanoate
PWY-5182	methylcatechol)	H2O
PWY-5182	methylcatechol)	(2Z)-2-hydroxypenta-2,4-dienoate

PWY-5182	methylcatechol)	pyruvate
PWY-5182	methylcatechol)	(S)-4-hydroxy-2-oxopentanoate
PWY-5182	methylcatechol)	H+
PWY-5182	methylcatechol)	NADH
PWY-5182	methylcatechol)	acetyl-CoA
PWY-5182	methylcatechol)	acetaldehyde
PWY-5182	methylcatechol)	coenzyme A
PWY-5182	methylcatechol)	NAD+
PWY-5304	ambivalens)	hydrogen sulfide
PWY-5304	ambivalens)	S0
PWY-5304	ambivalens)	oxygen
PWY-5304	ambivalens)	AMP
PWY-5304	ambivalens)	phosphate
PWY-5304	ambivalens)	adenosine 5'-phosphosulfate
PWY-5304	ambivalens)	ADP
PWY-5304	ambivalens)	a reduced c-type cytochrome
PWY-5304	ambivalens)	sulfate
PWY-5304	ambivalens)	an oxidized c-type cytochrome
PWY-5304	ambivalens)	a quinol
PWY-5304	ambivalens)	thiosulfate
PWY-5304	ambivalens)	an electron-transfer quinone
PWY-5304	ambivalens)	tetrathionate
PWY-5304	ambivalens)	a [DsrE3A thiosulfate-carrier protein]-L-cysteine
PWY-5304	ambivalens)	a [DsrE3A thiosulfate-carrier protein]-L-cysteine-S-thiosulfonate
PWY-5304	ambivalens)	a reduced unknown electron carrier
PWY-5304	ambivalens)	H+
PWY-5304	ambivalens)	sulfite
PWY-5304	ambivalens)	a [TusA]-L-cysteine
PWY-5304	ambivalens)	a [TusA]-L-cysteine-S-thiosulfonate
PWY-5304	ambivalens)	H2O
PWY-5304	ambivalens)	an oxidized unknown electron carrier
CHARGING-	tRNA charging	an L-arginyl-[tRNAArg]
CHARGING-	tRNA charging	a tRNAArg
CHARGING-	tRNA charging	L-arginine
CHARGING-	tRNA charging	an L-cysteinyl-[tRNACys]
CHARGING-	tRNA charging	a tRNACys
CHARGING-	tRNA charging	L-cysteine
CHARGING-	tRNA charging	an L-glutaminyl-[tRNAGln]
CHARGING-	tRNA charging	a tRNAGln
CHARGING-	tRNA charging	L-glutamine
CHARGING-	tRNA charging	an L-glutamyl-[tRNAGlu]
CHARGING-	tRNA charging	a tRNAglu
CHARGING-	tRNA charging	L-glutamate
CHARGING-	tRNA charging	an L-isoleucyl-[tRNAIle]
CHARGING-	tRNA charging	a tRNAIle
CHARGING-	tRNA charging	L-isoleucine
CHARGING-	tRNA charging	an L-leucyl-[tRNALeu]
CHARGING-	tRNA charging	a tRNALeu
CHARGING-	tRNA charging	L-leucine
CHARGING-	tRNA charging	an L-valyl-[tRNAVal]
CHARGING-	tRNA charging	a tRNAVal
CHARGING-	tRNA charging	L-valine
CHARGING-	tRNA charging	an L-alanyl-[tRNAAla]
CHARGING-	tRNA charging	a tRNAAla
CHARGING-	tRNA charging	L-alanine
CHARGING-	tRNA charging	an L-asparaginyl-[tRNAAsn]
CHARGING-	tRNA charging	tRNAAsn
CHARGING-	tRNA charging	L-asparagine
CHARGING-	tRNA charging	an L-aspartyl-[tRNAasp]
CHARGING-	tRNA charging	tRNAasp
CHARGING-	tRNA charging	L-aspartate
CHARGING-	tRNA charging	an L-tyrosyl-[tRNATyr]
CHARGING-	tRNA charging	a tRNATyr
CHARGING-	tRNA charging	L-tyrosine
CHARGING-	tRNA charging	an L-tryptophanyl-[tRNATrp]
CHARGING-	tRNA charging	L-tryptophan
CHARGING-	tRNA charging	a tRNATrp
CHARGING-	tRNA charging	an L-threonyl-[tRNAThr]
CHARGING-	tRNA charging	a tRNAThr
CHARGING-	tRNA charging	L-threonine
CHARGING-	tRNA charging	an L-seryl-[tRNAser]
CHARGING-	tRNA charging	a tRNAser

CHARGING-	tRNA charging	L-serine
CHARGING-	tRNA charging	an L-prolyl-[tRNAPro]
CHARGING-	tRNA charging	a tRNAPro
CHARGING-	tRNA charging	L-proline
CHARGING-	tRNA charging	an L-phenylalanyl-[tRNAPhe]
CHARGING-	tRNA charging	a tRNAPhe
CHARGING-	tRNA charging	L-phenylalanine
CHARGING-	tRNA charging	an L-methionyl-[elongator tRNAMet]
CHARGING-	tRNA charging	elongator tRNAMet
CHARGING-	tRNA charging	an L-lysyl-[tRNALys]
CHARGING-	tRNA charging	L-lysine
CHARGING-	tRNA charging	a tRNALys
CHARGING-	tRNA charging	an L-histidyl-[tRNAHis]
CHARGING-	tRNA charging	a tRNAHis
CHARGING-	tRNA charging	L-histidine
CHARGING-	tRNA charging	a glycyl-[tRNAGly]
CHARGING-	tRNA charging	a tRNAGly
CHARGING-	tRNA charging	glycine
CHARGING-	tRNA charging	AMP
CHARGING-	tRNA charging	diphosphate
CHARGING-	tRNA charging	an L-methionyl-[initiator tRNAMet]
CHARGING-	tRNA charging	initiator tRNAMet
CHARGING-	tRNA charging	L-methionine
CHARGING-	tRNA charging	ATP
PWY-6737	starch degradation V	a phosphorylated phosphoglucomutase
PWY-6737	starch degradation V	α-glucose 1,6-bisphosphate
PWY-6737	starch degradation V	a phosphoglucomutase
PWY-6737	starch degradation V	starch
PWY-6737	starch degradation V	H2O
PWY-6737	starch degradation V	phosphate
PWY-6737	starch degradation V	maltose
PWY-6737	starch degradation V	a maltodextrin
PWY-6737	starch degradation V	D-glucopyranose
PWY-6737	starch degradation V	D-glucopyranose 6-phosphate
PWY-6737	starch degradation V	α-D-glucopyranose 1-phosphate
HISDEG-PWY	L-histidine degradation I	ammonium
HISDEG-PWY	L-histidine degradation I	L-histidine
HISDEG-PWY	L-histidine degradation I	urocanate
HISDEG-PWY	L-histidine degradation I	4-imidazolone-5-propanoate
HISDEG-PWY	L-histidine degradation I	formamide
HISDEG-PWY	L-histidine degradation I	L-glutamate
HISDEG-PWY	L-histidine degradation I	H2O
HISDEG-PWY	L-histidine degradation I	N-formimino-L-glutamate
THISYN-PWY	biosynthesis I	carbon monoxide
THISYN-PWY	biosynthesis I	formate
THISYN-PWY	biosynthesis I	4-amino-2-methyl-5-(phosphooxymethyl)pyrimidine
THISYN-PWY	biosynthesis I	5-amino-1-(5-phospho-β-D-ribosyl)imidazole
THISYN-PWY	biosynthesis I	ADP
THISYN-PWY	biosynthesis I	thiamine diphosphate
THISYN-PWY	biosynthesis I	thiamine phosphate
THISYN-PWY	biosynthesis I	4-amino-2-methyl-5-(diphosphooxymethyl)pyrimidine
THISYN-PWY	biosynthesis I	H2O
THISYN-PWY	biosynthesis I	2-[(2R,5Z)-2-carboxy-4-methylthiazol-5(2H)-ylidene]ethyl phosphate
THISYN-PWY	biosynthesis I	CO2
THISYN-PWY	biosynthesis I	1-deoxy-D-xylulose 5-phosphate
THISYN-PWY	biosynthesis I	pyruvate
THISYN-PWY	biosynthesis I	D-glyceraldehyde 3-phosphate
THISYN-PWY	biosynthesis I	an oxidized ferredoxin [iron-sulfur] cluster
THISYN-PWY	biosynthesis I	AMP
THISYN-PWY	biosynthesis I	a thiocarboxy-[ThiS-Protein]
THISYN-PWY	biosynthesis I	a reduced ferredoxin [iron-sulfur] cluster
THISYN-PWY	biosynthesis I	diphosphate
THISYN-PWY	biosynthesis I	a carboxy-adenylated-[ThiS sulfur-carrier protein]
THISYN-PWY	biosynthesis I	a ThiS sulfur-carrier protein
THISYN-PWY	biosynthesis I	ATP
THISYN-PWY	biosynthesis I	H+
THISYN-PWY	biosynthesis I	NADP+
THISYN-PWY	biosynthesis I	L-methionine
THISYN-PWY	biosynthesis I	5'-deoxyadenosine
THISYN-PWY	biosynthesis I	4-methylphenol
THISYN-PWY	biosynthesis I	2-iminoacetate
THISYN-PWY	biosynthesis I	S-adenosyl-L-methionine

THISYN-PWY	biosynthesis I	L-tyrosine
THISYN-PWY	biosynthesis I	NADPH
THISYN-PWY	biosynthesis I	a [ThiI sulfur-carrier protein]-S-sulfanyl-L-cysteine
THISYN-PWY	biosynthesis I	a [ThiI sulfur-carrier protein]-L-cysteine
THISYN-PWY	biosynthesis I	an [L-cysteine desulfurase]-S-sulfanyl-L-cysteine
THISYN-PWY	biosynthesis I	L-alanine
THISYN-PWY	biosynthesis I	L-cysteine
THISYN-PWY	biosynthesis I	an [L-cysteine desulfurase]-L-cysteine
PWY0-41	allantoin degradation IV (anaerobic)	(S)-(+)-allantoin
PWY0-41	allantoin degradation IV (anaerobic)	allantoate
PWY0-41	allantoin degradation IV (anaerobic)	(S)-ureidoglycine
PWY0-41	allantoin degradation IV (anaerobic)	H2O
PWY0-41	allantoin degradation IV (anaerobic)	NAD(P)H
PWY0-41	allantoin degradation IV (anaerobic)	NAD(P)+
PWY0-41	allantoin degradation IV (anaerobic)	phosphate
PWY0-41	allantoin degradation IV (anaerobic)	oxamate
PWY0-41	allantoin degradation IV (anaerobic)	NADH
PWY0-41	allantoin degradation IV (anaerobic)	N-carbamoyl-2-oxoglycine
PWY0-41	allantoin degradation IV (anaerobic)	(S)-ureidoglycolate
PWY0-41	allantoin degradation IV (anaerobic)	NAD+
PWY0-41	allantoin degradation IV (anaerobic)	ADP
PWY0-41	allantoin degradation IV (anaerobic)	carbamoyl phosphate
PWY0-41	allantoin degradation IV (anaerobic)	ATP
PWY0-41	allantoin degradation IV (anaerobic)	CO2
PWY0-41	allantoin degradation IV (anaerobic)	ammonium
PWY0-41	allantoin degradation IV (anaerobic)	carbamate
PWY0-41	allantoin degradation IV (anaerobic)	H+
PWY-6147	biosynthesis I	AMP
PWY-6147	biosynthesis I	(7,8-dihydropterin-6-yl)methyl diphosphate
PWY-6147	biosynthesis I	ATP
PWY-6147	biosynthesis I	6-(hydroxymethyl)-7,8-dihydropterin
PWY-6147	biosynthesis I	glycolaldehyde
PWY-6147	biosynthesis I	phosphate
PWY-6147	biosynthesis I	D-erythro-7,8-dihydroneopterin
PWY-6147	biosynthesis I	diphosphate
PWY-6147	biosynthesis I	7,8-dihydroneopterin 3'-phosphate
PWY-6147	biosynthesis I	7,8-dihydroneopterin 3'-triphosphate
PWY-6147	biosynthesis I	formate
PWY-6147	biosynthesis I	H+
PWY-6147	biosynthesis I	GTP
PWY-6147	biosynthesis I	H2O
PWY-7208	superpathway of pyrimidine nucleobases salvage	ammonium
PWY-7208	superpathway of pyrimidine nucleobases salvage	UDP
PWY-7208	superpathway of pyrimidine nucleobases salvage	L-glutamate
PWY-7208	superpathway of pyrimidine nucleobases salvage	CTP
PWY-7208	superpathway of pyrimidine nucleobases salvage	phosphate
PWY-7208	superpathway of pyrimidine nucleobases salvage	ADP
PWY-7208	superpathway of pyrimidine nucleobases salvage	H+
PWY-7208	superpathway of pyrimidine nucleobases salvage	ATP
PWY-7208	superpathway of pyrimidine nucleobases salvage	UTP
PWY-7208	superpathway of pyrimidine nucleobases salvage	L-glutamine
PWY-7208	superpathway of pyrimidine nucleobases salvage	H2O
PWY-7208	superpathway of pyrimidine nucleobases salvage	uracil
PWY-7208	superpathway of pyrimidine nucleobases salvage	5-phospho-α-D-ribose 1-diphosphate
PWY-7208	superpathway of pyrimidine nucleobases salvage	diphosphate
PWY-7208	superpathway of pyrimidine nucleobases salvage	UMP
NSYN-PWY	polyisoprenoid biosynthesis (E. coli)	geranyl diphosphate
NSYN-PWY	polyisoprenoid biosynthesis (E. coli)	prenyl diphosphate
NSYN-PWY	polyisoprenoid biosynthesis (E. coli)	phosphate
NSYN-PWY	polyisoprenoid biosynthesis (E. coli)	di-trans,octa-cis-undecaprenyl phosphate
NSYN-PWY	polyisoprenoid biosynthesis (E. coli)	H+
NSYN-PWY	polyisoprenoid biosynthesis (E. coli)	di-trans,octa-cis-undecaprenyl diphosphate
NSYN-PWY	polyisoprenoid biosynthesis (E. coli)	H2O
NSYN-PWY	polyisoprenoid biosynthesis (E. coli)	diphosphate
NSYN-PWY	polyisoprenoid biosynthesis (E. coli)	all-trans-octaprenyl diphosphate
NSYN-PWY	polyisoprenoid biosynthesis (E. coli)	(2E,6E)-farnesyl diphosphate
NSYN-PWY	polyisoprenoid biosynthesis (E. coli)	isopentenyl diphosphate
YN-PWY	L-lysine biosynthesis I	CO2
YN-PWY	L-lysine biosynthesis I	L-lysine
YN-PWY	L-lysine biosynthesis I	meso-diaminopimelate
YN-PWY	L-lysine biosynthesis I	succinate
YN-PWY	L-lysine biosynthesis I	L,L-diaminopimelate

YN-PWY	L-lysine biosynthesis I	L-glutamate
YN-PWY	L-lysine biosynthesis I	2-oxoglutarate
YN-PWY	L-lysine biosynthesis I	N-succinyl-L,L-2,6-diaminopimelate
YN-PWY	L-lysine biosynthesis I	coenzyme A
YN-PWY	L-lysine biosynthesis I	N-succinyl-2-amino-6-ketopimelate
YN-PWY	L-lysine biosynthesis I	succinyl-CoA
YN-PWY	L-lysine biosynthesis I	pyruvate
YN-PWY	L-lysine biosynthesis I	NADPH
YN-PWY	L-lysine biosynthesis I	L-aspartate 4-semialdehyde
YN-PWY	L-lysine biosynthesis I	phosphate
YN-PWY	L-lysine biosynthesis I	NADP+
YN-PWY	L-lysine biosynthesis I	ADP
YN-PWY	L-lysine biosynthesis I	L-aspartyl-4-phosphate
YN-PWY	L-lysine biosynthesis I	L-aspartate
YN-PWY	L-lysine biosynthesis I	ATP
YN-PWY	L-lysine biosynthesis I	H+
YN-PWY	L-lysine biosynthesis I	NAD(P)H
YN-PWY	L-lysine biosynthesis I	(2S,4S)-4-hydroxy-2,3,4,5-tetrahydrodipicolinate
YN-PWY	L-lysine biosynthesis I	(S)-2,3,4,5-tetrahydrodipicolinate
YN-PWY	L-lysine biosynthesis I	NAD(P)+
YN-PWY	L-lysine biosynthesis I	H2O
PWY-5154	citrulline)	ammonium
PWY-5154	citrulline)	carbamate
PWY-5154	citrulline)	carboxyphosphate
PWY-5154	citrulline)	AMP
PWY-5154	citrulline)	diphosphate
PWY-5154	citrulline)	L-aspartate
PWY-5154	citrulline)	fumarate
PWY-5154	citrulline)	L-arginine
PWY-5154	citrulline)	L-arginino-succinate
PWY-5154	citrulline)	L-glutamine
PWY-5154	citrulline)	hydrogencarbonate
PWY-5154	citrulline)	carbamoyl phosphate
PWY-5154	citrulline)	acetate
PWY-5154	citrulline)	L-citrulline
PWY-5154	citrulline)	N-acetyl-L-citrulline
PWY-5154	citrulline)	H2O
PWY-5154	citrulline)	coenzyme A
PWY-5154	citrulline)	acetyl-CoA
PWY-5154	citrulline)	ADP
PWY-5154	citrulline)	N-acetyl-L-glutamate
PWY-5154	citrulline)	ATP
PWY-5154	citrulline)	NADPH
PWY-5154	citrulline)	N-acetylglutamyl-phosphate
PWY-5154	citrulline)	H+
PWY-5154	citrulline)	NADP+
PWY-5154	citrulline)	phosphate
PWY-5154	citrulline)	N-acetyl-L-glutamate 5-semialdehyde
PWY-5154	citrulline)	L-glutamate
PWY-5154	citrulline)	N-acetyl-L-ornithine
PWY-5154	citrulline)	2-oxoglutarate
PWY	L-rhamnose degradation I	glycerone phosphate
PWY	L-rhamnose degradation I	(S)-lactaldehyde
PWY	L-rhamnose degradation I	ADP
PWY	L-rhamnose degradation I	L-rhamnulose 1-phosphate
PWY	L-rhamnose degradation I	H+
PWY	L-rhamnose degradation I	ATP
PWY	L-rhamnose degradation I	L-rhamnulofuranose
PWY	L-rhamnose degradation I	keto-L-rhamnulose
PWY	L-rhamnose degradation I	β-L-rhamnopyranose
PWY	L-rhamnose degradation I	α-L-rhamnopyranose
PWY-7237	myo-, chiro- and scillo-inositol degradation	scyllo-inositol
PWY-7237	myo-, chiro- and scillo-inositol degradation	1D-chiro-inositol
PWY-7237	myo-, chiro- and scillo-inositol degradation	1-keto-D-chiro-inositol
PWY-7237	myo-, chiro- and scillo-inositol degradation	myo-inositol
PWY-7237	myo-, chiro- and scillo-inositol degradation	ADP
PWY-7237	myo-, chiro- and scillo-inositol degradation	ATP
PWY-7237	myo-, chiro- and scillo-inositol degradation	glycerone phosphate
PWY-7237	myo-, chiro- and scillo-inositol degradation	5-dehydro-2-deoxy-D-gluconate 6-phosphate
PWY-7237	myo-, chiro- and scillo-inositol degradation	NADH
PWY-7237	myo-, chiro- and scillo-inositol degradation	CO2
PWY-7237	myo-, chiro- and scillo-inositol degradation	acetyl-CoA

PWY-7237	myo-, chiro- and scillo-inositol degradation	3-oxopropanoate
PWY-7237	myo-, chiro- and scillo-inositol degradation	coenzyme A
PWY-7237	myo-, chiro- and scillo-inositol degradation	NAD+
PWY-7237	myo-, chiro- and scillo-inositol degradation	scyllo-inosose
PWY-7237	myo-, chiro- and scillo-inositol degradation	H+
PWY-7237	myo-, chiro- and scillo-inositol degradation	3D-(3,5/4)-trihydroxycyclohexane-1,2-dione
PWY-7237	myo-, chiro- and scillo-inositol degradation	H2O
PWY-7237	myo-, chiro- and scillo-inositol degradation	5-dehydro-2-deoxy-D-gluconate
PWY-7237	myo-, chiro- and scillo-inositol degradation	5-deoxy-D-glucuronate
PWY-6470	resistance)	phosphoenolpyruvate
PWY-6470	resistance)	UDP-N-acetyl-α-D-glucosamine-enolpyruvate
PWY-6470	resistance)	NADPH
PWY-6470	resistance)	NADP+
PWY-6470	resistance)	L-alanine
PWY-6470	resistance)	UDP-N-acetyl-α-D-muramate
PWY-6470	resistance)	UDP-N-acetyl-α-D-muramoyl-L-alanine
PWY-6470	resistance)	D-glutamate
PWY-6470	resistance)	L-glutamate
PWY-6470	resistance)	UDP-N-acetyl-α-D-muramoyl-L-alanyl-D-glutamate
PWY-6470	resistance)	L-lysine
PWY-6470	resistance)	UDP-N-acetyl-α-D-muramoyl-L-alanyl-γ-D-glutamyl-L-lysine
PWY-6470	resistance)	D-alanyl-D-alanine
PWY-6470	resistance)	alanine
PWY-6470	resistance)	UMP
PWY-6470	resistance)	UDP-N-acetyl-α-D-muramoyl-L-alanyl-γ-D-glutamyl-L-lysyl-D-alanine
PWY-6470	resistance)	di-trans,octa-cis-undecaprenyl phosphate
PWY-6470	resistance)	UDP
PWY-6470	resistance)	lysyl- D-alanine
PWY-6470	resistance)	UDP-N-acetyl-α-D-glucosamine
PWY-6470	resistance)	acetylglucosaminyl)muramoyl-L-alanyl-γ-D-glutamyl-L-lysyl-D-alanine
PWY-6470	resistance)	phosphate
PWY-6470	resistance)	ADP
PWY-6470	resistance)	acetylglucosaminyl)muramoyl-L-alanyl-γ-D-isoglutaminyl-L-lysyl-D-
PWY-6470	resistance)	D-aspartate
PWY-6470	resistance)	ATP
PWY-6470	resistance)	H2O
PWY-6470	resistance)	acetylglucosaminyl)muramoyl-L-alanyl-γ-D-isoglutaminyl-N-(β-D-
PWY-6470	resistance)	ammonium
PWY-6470	resistance)	acetylglucosaminyl)muramoyl-L-alanyl-γ-D-isoglutaminyl-N-(β-D-
PWY-6470	resistance)	D-alanine
PWY-6470	resistance)	faecium)
PWY-6470	resistance)	a peptidoglycan dimer (E. faecium, tetrapeptide)
PWY-6470	resistance)	H+
PWY-6470	resistance)	pentapeptide
PWY-6470	resistance)	di-trans,octa-cis-undecaprenyl diphosphate
PWY-6470	resistance)	glucosaminyl)muramoyl-L-alanyl-γ-D-glutamyl-L-lysyl-D-alanyl-D-
PWY-5100	pyruvate fermentation to acetate and lactate II	a reduced ferredoxin [iron-sulfur] cluster
PWY-5100	pyruvate fermentation to acetate and lactate II	CO2
PWY-5100	pyruvate fermentation to acetate and lactate II	an oxidized ferredoxin [iron-sulfur] cluster
PWY-5100	pyruvate fermentation to acetate and lactate II	ADP
PWY-5100	pyruvate fermentation to acetate and lactate II	ATP
PWY-5100	pyruvate fermentation to acetate and lactate II	acetate
PWY-5100	pyruvate fermentation to acetate and lactate II	coenzyme A
PWY-5100	pyruvate fermentation to acetate and lactate II	acetyl phosphate
PWY-5100	pyruvate fermentation to acetate and lactate II	phosphate
PWY-5100	pyruvate fermentation to acetate and lactate II	acetyl-CoA
PWY-5100	pyruvate fermentation to acetate and lactate II	H+
PWY-5100	pyruvate fermentation to acetate and lactate II	NADH
PWY-5100	pyruvate fermentation to acetate and lactate II	pyruvate
PWY-5100	pyruvate fermentation to acetate and lactate II	(S)-lactate
PWY-5100	pyruvate fermentation to acetate and lactate II	NAD+
P562-PWY	myo-inositol degradation I	ADP
P562-PWY	myo-inositol degradation I	ATP
P562-PWY	myo-inositol degradation I	glycerone phosphate
P562-PWY	myo-inositol degradation I	5-dehydro-2-deoxy-D-gluconate 6-phosphate
P562-PWY	myo-inositol degradation I	CO2
P562-PWY	myo-inositol degradation I	acetyl-CoA
P562-PWY	myo-inositol degradation I	3-oxopropanoate
P562-PWY	myo-inositol degradation I	coenzyme A
P562-PWY	myo-inositol degradation I	NADH
P562-PWY	myo-inositol degradation I	myo-inositol
P562-PWY	myo-inositol degradation I	NAD+

P562-PWY	myo-inositol degradation I	scyllo-inosose
P562-PWY	myo-inositol degradation I	H+
P562-PWY	myo-inositol degradation I	3D-(3,5/4)-trihydroxycyclohexane-1,2-dione
P562-PWY	myo-inositol degradation I	H2O
P562-PWY	myo-inositol degradation I	5-dehydro-2-deoxy-D-gluconate
P562-PWY	myo-inositol degradation I	5-deoxy-D-glucuronate
PWY-7254	TCA cycle VII (acetate-producers)	a [2-oxoglutarate dehydrogenase E2 protein] N6-lipoyl-L-lysine
PWY-7254	TCA cycle VII (acetate-producers)	succinyldihydrolipoyl-L-lysine
PWY-7254	TCA cycle VII (acetate-producers)	a [2-oxoglutarate dehydrogenase E2 protein] N6-dihydrolipoyl-L-lysine
PWY-7254	TCA cycle VII (acetate-producers)	oxalosuccinate
PWY-7254	TCA cycle VII (acetate-producers)	E-
PWY-7254	TCA cycle VII (acetate-producers)	NADH
PWY-7254	TCA cycle VII (acetate-producers)	NAD+
PWY-7254	TCA cycle VII (acetate-producers)	H+
PWY-7254	TCA cycle VII (acetate-producers)	coenzyme A
PWY-7254	TCA cycle VII (acetate-producers)	H2O
PWY-7254	TCA cycle VII (acetate-producers)	cis-aconitate
PWY-7254	TCA cycle VII (acetate-producers)	citrate
PWY-7254	TCA cycle VII (acetate-producers)	oxaloacetate
PWY-7254	TCA cycle VII (acetate-producers)	(S)-malate
PWY-7254	TCA cycle VII (acetate-producers)	NADPH
PWY-7254	TCA cycle VII (acetate-producers)	CO2
PWY-7254	TCA cycle VII (acetate-producers)	2-oxoglutarate
PWY-7254	TCA cycle VII (acetate-producers)	D-threo-isocitrate
PWY-7254	TCA cycle VII (acetate-producers)	NADP+
PWY-7254	TCA cycle VII (acetate-producers)	acetyl-CoA
PWY-7254	TCA cycle VII (acetate-producers)	succinyl-CoA
PWY-7254	TCA cycle VII (acetate-producers)	acetate
PWY-7254	TCA cycle VII (acetate-producers)	fumarate
PWY-7254	TCA cycle VII (acetate-producers)	an electron-transfer quinol
PWY-7254	TCA cycle VII (acetate-producers)	an electron-transfer quinone
PWY-7254	TCA cycle VII (acetate-producers)	succinate
PWY-5265	peptidoglycan biosynthesis II (staphylococci)	ammonium
PWY-5265	peptidoglycan biosynthesis II (staphylococci)	glucosaminyl)muramoyl-L-alanyl-γ-O-phospho-D-glutamyl-L-lysyl-D-
PWY-5265	peptidoglycan biosynthesis II (staphylococci)	ammonia
PWY-5265	peptidoglycan biosynthesis II (staphylococci)	UMP
PWY-5265	peptidoglycan biosynthesis II (staphylococci)	di-trans,octa-cis-undecaprenyl phosphate
PWY-5265	peptidoglycan biosynthesis II (staphylococci)	UDP
PWY-5265	peptidoglycan biosynthesis II (staphylococci)	lysyl- D-alanyl-D-alanine
PWY-5265	peptidoglycan biosynthesis II (staphylococci)	UDP-N-acetyl-α-D-glucosamine
PWY-5265	peptidoglycan biosynthesis II (staphylococci)	phosphoenolpyruvate
PWY-5265	peptidoglycan biosynthesis II (staphylococci)	UDP-N-acetyl-α-D-glucosamine-enolpyruvate
PWY-5265	peptidoglycan biosynthesis II (staphylococci)	NADPH
PWY-5265	peptidoglycan biosynthesis II (staphylococci)	NADP+
PWY-5265	peptidoglycan biosynthesis II (staphylococci)	L-alanine
PWY-5265	peptidoglycan biosynthesis II (staphylococci)	UDP-N-acetyl-α-D-muramate
PWY-5265	peptidoglycan biosynthesis II (staphylococci)	UDP-N-acetyl-α-D-muramoyl-L-alanine
PWY-5265	peptidoglycan biosynthesis II (staphylococci)	D-glutamate
PWY-5265	peptidoglycan biosynthesis II (staphylococci)	UDP-N-acetyl-α-D-muramoyl-L-alanyl-D-glutamate
PWY-5265	peptidoglycan biosynthesis II (staphylococci)	L-lysine
PWY-5265	peptidoglycan biosynthesis II (staphylococci)	alanine
PWY-5265	peptidoglycan biosynthesis II (staphylococci)	UDP-N-acetyl-α-D-muramoyl-L-alanyl-γ-D-glutamyl-L-lysine
PWY-5265	peptidoglycan biosynthesis II (staphylococci)	D-alanyl-D-alanine
PWY-5265	peptidoglycan biosynthesis II (staphylococci)	D-alanine
PWY-5265	peptidoglycan biosynthesis II (staphylococci)	a peptidoglycan with D,D cross-link (S. aureus)
PWY-5265	peptidoglycan biosynthesis II (staphylococci)	a peptidoglycan dimer (S. aureus)
PWY-5265	peptidoglycan biosynthesis II (staphylococci)	acetylglucosaminyl)muramoyl-L-alanyl-γ-D-isoglutaminyl-L-lysyl-(N6-
PWY-5265	peptidoglycan biosynthesis II (staphylococci)	a tRNAGly
PWY-5265	peptidoglycan biosynthesis II (staphylococci)	acetylglucosaminyl)muramoyl-L-alanyl-γ-D-isoglutaminyl-L-lysyl-
PWY-5265	peptidoglycan biosynthesis II (staphylococci)	acetylglucosaminyl)muramoyl-L-alanyl-γ-D-isoglutaminyl-L-lysyl-
PWY-5265	peptidoglycan biosynthesis II (staphylococci)	a glycyl-[tRNAGly]
PWY-5265	peptidoglycan biosynthesis II (staphylococci)	pentapeptide
PWY-5265	peptidoglycan biosynthesis II (staphylococci)	di-trans,octa-cis-undecaprenyl diphosphate
PWY-5265	peptidoglycan biosynthesis II (staphylococci)	H+
PWY-5265	peptidoglycan biosynthesis II (staphylococci)	phosphate
PWY-5265	peptidoglycan biosynthesis II (staphylococci)	ADP
PWY-5265	peptidoglycan biosynthesis II (staphylococci)	acetylglucosaminyl)muramoyl-L-alanyl-γ-D-isoglutaminyl-L-lysyl-D-
PWY-5265	peptidoglycan biosynthesis II (staphylococci)	L-glutamate
PWY-5265	peptidoglycan biosynthesis II (staphylococci)	L-glutamine
PWY-5265	peptidoglycan biosynthesis II (staphylococci)	glucosaminyl)muramoyl-L-alanyl-γ-D-glutamyl-L-lysyl-D-alanyl-D-
PWY-5265	peptidoglycan biosynthesis II (staphylococci)	ATP
PWY-5265	peptidoglycan biosynthesis II (staphylococci)	H2O

PWY	biosynthesis	NADPH
PWY	biosynthesis	NADP+
PWY	biosynthesis	a reduced thioredoxin
PWY	biosynthesis	adenosine 3',5'-bisphosphate
PWY	biosynthesis	sulfite
PWY	biosynthesis	an oxidized thioredoxin
PWY	biosynthesis	diphosphate
PWY	biosynthesis	sulfate
PWY	biosynthesis	ADP
PWY	biosynthesis	3'-phosphoadenylyl-sulfate
PWY	biosynthesis	adenosine 5'-phosphosulfate
PWY	biosynthesis	ATP
PWY	biosynthesis	coenzyme A
PWY	biosynthesis	acetyl-CoA
PWY	biosynthesis	acetate
PWY	biosynthesis	L-cysteine
PWY	biosynthesis	O-acetyl-L-serine
PWY	biosynthesis	hydrogen sulfide
PWY	biosynthesis	L-glutamate
PWY	biosynthesis	2-oxoglutarate
PWY	biosynthesis	NADH
PWY	biosynthesis	3-phosphooxypyruvate
PWY	biosynthesis	H+
PWY	biosynthesis	3-phospho-D-glycerate
PWY	biosynthesis	NAD+
PWY	biosynthesis	phosphate
PWY	biosynthesis	L-serine
PWY	biosynthesis	O-phospho-L-serine
PWY	biosynthesis	H2O
PWY-5345	sulfhydrylation)	L-glutamate
PWY-5345	sulfhydrylation)	oxaloacetate
PWY-5345	sulfhydrylation)	2-oxoglutarate
PWY-5345	sulfhydrylation)	L-aspartate
PWY-5345	sulfhydrylation)	L-aspartyl-4-phosphate
PWY-5345	sulfhydrylation)	phosphate
PWY-5345	sulfhydrylation)	NAD(P)H
PWY-5345	sulfhydrylation)	L-aspartate 4-semialdehyde
PWY-5345	sulfhydrylation)	NAD(P)+
PWY-5345	sulfhydrylation)	acetate
PWY-5345	sulfhydrylation)	coenzyme A
PWY-5345	sulfhydrylation)	O-acetyl-L-homoserine
PWY-5345	sulfhydrylation)	L-homoserine
PWY-5345	sulfhydrylation)	acetyl-CoA
PWY-5345	sulfhydrylation)	tetrahydropteroyl tri-L-glutamate
PWY-5345	sulfhydrylation)	5-methyltetrahydropteroyl tri-L-glutamate
PWY-5345	sulfhydrylation)	a tetrahydrofolate
PWY-5345	sulfhydrylation)	L-methionine
PWY-5345	sulfhydrylation)	L-homocysteine
PWY-5345	sulfhydrylation)	a 5-methyltetrahydrofolate
PWY-5345	sulfhydrylation)	NADPH
PWY-5345	sulfhydrylation)	H2O
PWY-5345	sulfhydrylation)	NADP+
PWY-5345	sulfhydrylation)	hydrogen sulfide
PWY-5345	sulfhydrylation)	a reduced thioredoxin
PWY-5345	sulfhydrylation)	adenosine 3',5'-bisphosphate
PWY-5345	sulfhydrylation)	sulfite
PWY-5345	sulfhydrylation)	an oxidized thioredoxin
PWY-5345	sulfhydrylation)	diphosphate
PWY-5345	sulfhydrylation)	sulfate
PWY-5345	sulfhydrylation)	ADP
PWY-5345	sulfhydrylation)	3'-phosphoadenylyl-sulfate
PWY-5345	sulfhydrylation)	H+
PWY-5345	sulfhydrylation)	adenosine 5'-phosphosulfate
PWY-5345	sulfhydrylation)	ATP
N3-PWY	superpathway of polyamine biosynthesis II	S-adenosyl-L-methionine
N3-PWY	superpathway of polyamine biosynthesis II	L-ornithine
N3-PWY	superpathway of polyamine biosynthesis II	urea
N3-PWY	superpathway of polyamine biosynthesis II	L-arginine
N3-PWY	superpathway of polyamine biosynthesis II	agmatine
N3-PWY	superpathway of polyamine biosynthesis II	ammonium
N3-PWY	superpathway of polyamine biosynthesis II	CO2
N3-PWY	superpathway of polyamine biosynthesis II	putrescine

N3-PWY	superpathway of polyamine biosynthesis II	N-carbamoylputrescine
N3-PWY	superpathway of polyamine biosynthesis II	H2O
N3-PWY	superpathway of polyamine biosynthesis II	S-methyl-5'-thioadenosine
N3-PWY	superpathway of polyamine biosynthesis II	spermine
N3-PWY	superpathway of polyamine biosynthesis II	H+
N3-PWY	superpathway of polyamine biosynthesis II	spermidine
N3-PWY	superpathway of polyamine biosynthesis II	S-adenosyl 3-(methylsulfanyl)propylamine
RUMP-PWY	formaldehyde oxidation I	formaldehyde
RUMP-PWY	formaldehyde oxidation I	hexulose 6-phosphate
RUMP-PWY	formaldehyde oxidation I	H2O
RUMP-PWY	formaldehyde oxidation I	NADH
RUMP-PWY	formaldehyde oxidation I	CO2
RUMP-PWY	formaldehyde oxidation I	D-ribulose 5-phosphate
RUMP-PWY	formaldehyde oxidation I	D-gluconate 6-phosphate
RUMP-PWY	formaldehyde oxidation I	NAD+
RUMP-PWY	formaldehyde oxidation I	β-D-fructofuranose 6-phosphate
RUMP-PWY	formaldehyde oxidation I	H+
RUMP-PWY	formaldehyde oxidation I	NADPH
RUMP-PWY	formaldehyde oxidation I	6-phospho D-glucono-1,5-lactone
RUMP-PWY	formaldehyde oxidation I	D-glucopyranose 6-phosphate
RUMP-PWY	formaldehyde oxidation I	NADP+
PWY	superpathway of polyamine biosynthesis I	agmatine
PWY	superpathway of polyamine biosynthesis I	L-ornithine
PWY	superpathway of polyamine biosynthesis I	urea
PWY	superpathway of polyamine biosynthesis I	H2O
PWY	superpathway of polyamine biosynthesis I	L-arginine
PWY	superpathway of polyamine biosynthesis I	spermidine
PWY	superpathway of polyamine biosynthesis I	putrescine
PWY	superpathway of polyamine biosynthesis I	S-adenosyl-L-methionine
PWY	superpathway of polyamine biosynthesis I	S-methyl-5'-thioadenosine
PWY	superpathway of polyamine biosynthesis I	aminopropylcadaverine
PWY	superpathway of polyamine biosynthesis I	S-adenosyl 3-(methylsulfanyl)propylamine
PWY	superpathway of polyamine biosynthesis I	CO2
PWY	superpathway of polyamine biosynthesis I	cadaverine
PWY	superpathway of polyamine biosynthesis I	L-lysine
PWY	superpathway of polyamine biosynthesis I	H+
PWY-5838	superpathway of menaquinol-8 biosynthesis I	(2E,6E)-farnesyl diphosphate
PWY-5838	superpathway of menaquinol-8 biosynthesis I	isopentenyl diphosphate
PWY-5838	superpathway of menaquinol-8 biosynthesis I	AMP
PWY-5838	superpathway of menaquinol-8 biosynthesis I	ATP
PWY-5838	superpathway of menaquinol-8 biosynthesis I	2-succinylbenzoate
PWY-5838	superpathway of menaquinol-8 biosynthesis I	chorismate
PWY-5838	superpathway of menaquinol-8 biosynthesis I	isochorismate
PWY-5838	superpathway of menaquinol-8 biosynthesis I	2-oxoglutarate
PWY-5838	superpathway of menaquinol-8 biosynthesis I	pyruvate
PWY-5838	superpathway of menaquinol-8 biosynthesis I	(1R,6R)-6-hydroxy-2-succinylcyclohexa-2,4-diene-1-carboxylate
PWY-5838	superpathway of menaquinol-8 biosynthesis I	2-succinyl-5-enolpyruvoyl-6-hydroxy-3-cyclohexene-1-carboxylate
PWY-5838	superpathway of menaquinol-8 biosynthesis I	4-(2'-carboxyphenyl)-4-oxobutyryl-CoA
PWY-5838	superpathway of menaquinol-8 biosynthesis I	coenzyme A
PWY-5838	superpathway of menaquinol-8 biosynthesis I	1,4-dihydroxy-2-naphthoyl-CoA
PWY-5838	superpathway of menaquinol-8 biosynthesis I	H2O
PWY-5838	superpathway of menaquinol-8 biosynthesis I	S-adenosyl-L-homocysteine
PWY-5838	superpathway of menaquinol-8 biosynthesis I	menaquinol-8
PWY-5838	superpathway of menaquinol-8 biosynthesis I	S-adenosyl-L-methionine
PWY-5838	superpathway of menaquinol-8 biosynthesis I	CO2
PWY-5838	superpathway of menaquinol-8 biosynthesis I	diphosphate
PWY-5838	superpathway of menaquinol-8 biosynthesis I	demethylmenaquinol-8
PWY-5838	superpathway of menaquinol-8 biosynthesis I	all-trans-octaprenyl diphosphate
PWY-5838	superpathway of menaquinol-8 biosynthesis I	1,4-dihydroxy-2-naphthoate
PWY-5838	superpathway of menaquinol-8 biosynthesis I	H+
PWY-7229	biosynthesis I	GDP
PWY-7229	biosynthesis I	phosphate
PWY-7229	biosynthesis I	H+
PWY-7229	biosynthesis I	L-aspartate
PWY-7229	biosynthesis I	IMP
PWY-7229	biosynthesis I	GTP
PWY-7229	biosynthesis I	fumarate
PWY-7229	biosynthesis I	adenylo-succinate
PWY-7229	biosynthesis I	AMP
PWY-7229	biosynthesis I	a reduced thioredoxin
PWY-7229	biosynthesis I	an oxidized thioredoxin
PWY-7229	biosynthesis I	H2O

PWY-7229	biosynthesis I	ADP
PWY-7229	biosynthesis I	dATP
PWY-7229	biosynthesis I	dADP
PWY-7229	biosynthesis I	ATP
MINE-SYN	biosynthesis	ammonium
MINE-SYN	biosynthesis	carbamate
MINE-SYN	biosynthesis	carboxyphosphate
MINE-SYN	biosynthesis	agmatine
MINE-SYN	biosynthesis	urea
MINE-SYN	biosynthesis	spermidine
MINE-SYN	biosynthesis	putrescine
MINE-SYN	biosynthesis	S-adenosyl-L-methionine
MINE-SYN	biosynthesis	S-methyl-5'-thioadenosine
MINE-SYN	biosynthesis	aminopropylcadaverine
MINE-SYN	biosynthesis	S-adenosyl 3-(methylsulfanyl)propylamine
MINE-SYN	biosynthesis	CO2
MINE-SYN	biosynthesis	cadaverine
MINE-SYN	biosynthesis	L-lysine
MINE-SYN	biosynthesis	AMP
MINE-SYN	biosynthesis	diphosphate
MINE-SYN	biosynthesis	L-aspartate
MINE-SYN	biosynthesis	fumarate
MINE-SYN	biosynthesis	L-arginine
MINE-SYN	biosynthesis	L-arginino-succinate
MINE-SYN	biosynthesis	L-citrulline
MINE-SYN	biosynthesis	carbamoyl phosphate
MINE-SYN	biosynthesis	L-glutamine
MINE-SYN	biosynthesis	hydrogencarbonate
MINE-SYN	biosynthesis	coenzyme A
MINE-SYN	biosynthesis	acetyl-CoA
MINE-SYN	biosynthesis	ADP
MINE-SYN	biosynthesis	N-acetyl-L-glutamate
MINE-SYN	biosynthesis	ATP
MINE-SYN	biosynthesis	NADPH
MINE-SYN	biosynthesis	N-acetylglutamyl-phosphate
MINE-SYN	biosynthesis	H+
MINE-SYN	biosynthesis	NADP+
MINE-SYN	biosynthesis	phosphate
MINE-SYN	biosynthesis	N-acetyl-L-glutamate 5-semialdehyde
MINE-SYN	biosynthesis	L-glutamate
MINE-SYN	biosynthesis	2-oxoglutarate
MINE-SYN	biosynthesis	acetate
MINE-SYN	biosynthesis	L-ornithine
MINE-SYN	biosynthesis	N-acetyl-L-ornithine
MINE-SYN	biosynthesis	H2O
PWY-5840	superpathway of menaquinol-7 biosynthesis	AMP
PWY-5840	superpathway of menaquinol-7 biosynthesis	ATP
PWY-5840	superpathway of menaquinol-7 biosynthesis	2-succinylbenzoate
PWY-5840	superpathway of menaquinol-7 biosynthesis	chorismate
PWY-5840	superpathway of menaquinol-7 biosynthesis	isochorismate
PWY-5840	superpathway of menaquinol-7 biosynthesis	2-oxoglutarate
PWY-5840	superpathway of menaquinol-7 biosynthesis	pyruvate
PWY-5840	superpathway of menaquinol-7 biosynthesis	(1R,6R)-6-hydroxy-2-succinylcyclohexa-2,4-diene-1-carboxylate
PWY-5840	superpathway of menaquinol-7 biosynthesis	2-succinyl-5-enolpyruvoyl-6-hydroxy-3-cyclohexene-1-carboxylate
PWY-5840	superpathway of menaquinol-7 biosynthesis	4-(2'-carboxyphenyl)-4-oxobutyryl-CoA
PWY-5840	superpathway of menaquinol-7 biosynthesis	coenzyme A
PWY-5840	superpathway of menaquinol-7 biosynthesis	1,4-dihydroxy-2-naphthoyl-CoA
PWY-5840	superpathway of menaquinol-7 biosynthesis	H2O
PWY-5840	superpathway of menaquinol-7 biosynthesis	(2E,6E)-farnesyl diphosphate
PWY-5840	superpathway of menaquinol-7 biosynthesis	isopentenyl diphosphate
PWY-5840	superpathway of menaquinol-7 biosynthesis	CO2
PWY-5840	superpathway of menaquinol-7 biosynthesis	diphosphate
PWY-5840	superpathway of menaquinol-7 biosynthesis	all-trans-heptaprenyl diphosphate
PWY-5840	superpathway of menaquinol-7 biosynthesis	1,4-dihydroxy-2-naphthoate
PWY-5840	superpathway of menaquinol-7 biosynthesis	H+
PWY-5840	superpathway of menaquinol-7 biosynthesis	S-adenosyl-L-homocysteine
PWY-5840	superpathway of menaquinol-7 biosynthesis	menaquinol-7
PWY-5840	superpathway of menaquinol-7 biosynthesis	S-adenosyl-L-methionine
PWY-5840	superpathway of menaquinol-7 biosynthesis	demethylmenaquinol-7

Table S6. Metabolites derived from 16S predicted metagenomic pathways were annoated by HMDB IDs and KEGG IDs

Compounds derived from predicted pathways by 16S	Human Metabolome Database	KEGG
a (1→4)-α-D-glucan	HMDB0003403	C00718
(11Z)-3-oxo-icos-11-enoyl-[acp]	No result	No result
(11Z)-3-oxooctadec-11-enoyl-[acp]	No result	No result
(2E,11Z)-icosa-2,11-dienoyl-[acp]	No result	No result
(2E,11Z)-octadeca-2,11-dienoyl-[acp]	No result	No result
(2E,6E)-farnesyl diphosphate	HMDB0000961	C00448
(3R)-3-hydroxytetradecanoyl-[acp]	No result	No result
(3R,11Z)-3-hydroxy-icos-11-enoyl-[acp]	No result	No result
(3R,11Z)-3-hydroxyoctadec-11-enoyl-[acp]	No result	No result
(7,8-dihydropterin-6-yl)methyl diphosphate	No result	C04807
(E)-4-hydroxy-3-methylbut-2-en-1-yl diphosphate	No result	C11811
(R)-4'-phosphopantothenate	HMDB0001016	C03492
(R)-4'-phosphopantothenoyl-L-cysteine	HMDB0001117	C04352
(R)-lactate	HMDB0001311	C00256
(R)-methylmalonyl-CoA	HMDB0002255	C01213
(R)-pantoate	HMDB0240389	C00522
(R)-pantothenate	HMDB0000210	C00864
(S)-malate	HMDB0000156	C00149
(S)-methylmalonyl-CoA	HMDB0002310	C00683
[2-oxoglutarate dehydrogenase E2 protein] N6-dihydrolipoyl-L-lysine	No result	No result
[2-oxoglutarate dehydrogenase E2 protein] N6-lipoyl-L-lysine	No result	No result
[2-oxoglutarate dehydrogenase E2 protein] N6-S-succinyldihydrolipoyl-L-lysine	No result	No result
1-deoxy-D-xylulose 5-phosphate	HMDB0001213	C11437
1-deoxy-L-glycero-tetrulose 4-phosphate	No result	C15556
2-(formamido)-N1-(5-phospho-β-D-ribosyl)acetamidine	HMDB0006211	C04640
2,5-diamino-6-(5-phospho-D-ribosylamino)pyrimidin-4(3H)-one	No result	C01304
2-C-methyl-D-erythritol 4-phosphate	No result	C11434
2-C-methyl-D-erythritol-2,4-cyclodiphosphate	No result	C11453
2-dehydropantoate	No result	C00966
2'-deoxyadenosine	HMDB0000101	C00559
2'-deoxycytidine	HMDB0000014	C00881
2-deoxy-D-ribose 5-phosphate	HMDB0001031	C00673
2'-deoxyguanosine	HMDB0000085	C00330
2'-deoxyinosine	HMDB0000071	C05512
2'-deoxyuridine	HMDB0000012	C00526
2-deoxy-α-D-ribose 1-phosphate	No result	C00672
2-iminosuccinate	HMDB0001131	C05840
2-oxoglutarate	HMDB0000208	C00026
2-phospho-4-(cytidine 5'-diphospho)-2-C-methyl-D-erythritol	No result	C11436
3-deoxy-D-manno-octulosonate 8-phosphate	No result	C04478
3-deoxy-α-D-manno-2-octulosonate	No result	C01187
3'-dephospho-CoA	HMDB0001373	C00882
3-methyl-2-oxobutanoate	HMDB0000019	C00141
4-(cytidine 5'-diphospho)-2-C-methyl-D-erythritol	No result	C11435
4-amino-2-methyl-5-(diphosphooxymethyl)pyrimidine	No result	C04752
4-amino-2-methyl-5-(phosphooxymethyl)pyrimidine	No result	C04556
4-amino-2-methyl-5-pyrimidinemethanol	No result	C01279
4-amino-4-deoxychorismate	No result	C11355
4-aminobenzoate	HMDB0001392	C00568
4-methyl-5-(2-phosphooxyethyl)thiazole	No result	C04327
4'-phosphopantetheine	HMDB0001416	C01134
5-(2-hydroxyethyl)-4-methylthiazole	HMDB0032985	C04294
a 5,10-methenyltetrahydrofolate	HMDB0001354	C00445
5,10-methylenetetrahydrofolate	HMDB0001533	C00143
5-amino-1-(5-phospho-β-D-ribosyl)imidazole	HMDB0001235	C03373
5-amino-6-(5-phospho-D-ribitylamino)uracil	HMDB0003841	C04454
5-amino-6-(5-phospho-D-ribosylamino)uracil	No result	C01268
5-amino-6-(D-ribitylamino)uracil	HMDB0011106	C04732
5-methyltetrahydropteroyl tri-L-glutamate	HMDB0012177	C04489
5-phospho-α-D-ribose 1-diphosphate	HMDB0000280	C00119
5-phospho-β-D-ribosylamine	HMDB0001128	C03090
6-(hydroxymethyl)-7,8-dihydropterin	No result	C01300
6,7-dimethyl-8-(1-D-ribityl)lumazine	HMDB0003826	C04332
6-carboxy-5,6,7,8-tetrahydropterin	HMDB0060410	C20239
7,8-dihydrofolate	HMDB0001056	C00415
7,8-dihydrofolate monoglutamate	HMDB0001056	C00415
7,8-dihydroneopterin 3'-phosphate	HMDB0006824	C05925
7,8-dihydroneopterin 3'-triphosphate	HMDB0000980	C04895
7,8-dihydropteroate	HMDB0001412	C00921
7-aminomethyl-7-deazaguanosine34 in tRNA	No result	No result
7-carboxy-7-deazaguanine	No result	C20248
acetaldehyde	HMDB0000990	C00084
acetate	HMDB0000042	C00033
acetyl phosphate	HMDB0001494	C00227
acetyl-CoA	HMDB0001206	C00024
acetylene	No result	C01548

adenine	HMDB0000034	C00147
ADP	HMDB0001341	C00008
ADP-α-D-glucose	HMDB0006557	C00498
aldehydo-D-arabinose 5-phosphate	HMDB0011734	C01112
aminated amino group donor	No result	No result
ammonia	HMDB0000051	C00014
ammonium	HMDB0041827	C01342
AMP	HMDB0000045	C00020
ATP	HMDB0000538	C00002
autoinducer 2	No result	No result
chorismate	HMDB0012199	C00251
cis-aconitate	HMDB0000072	C00417
cis-vaccenate	No result	No result
cis-vaccenoyl-[acp]	No result	No result
citrate	HMDB0000094	C00158
CMP	HMDB0000095	C00055
CMP-3-deoxy-β-D-manno-octulosonate	No result	C04121
CO2	HMDB0001967	C00011
coenzyme A	HMDB0001423	C00010
CTP	HMDB0000082	C00063
D-alanine	HMDB0001310	C00133
D-alanyl-D-alanine	HMDB0003459	C00993
D-arabinofuranose 5-phosphate	No result	No result
D-aspartate	HMDB0006483	C00402
dCMP	HMDB0001202	C00239
deaminated amino group donor	No result	No result
decaprenyl-diphospho-N-acetylmuramoyl-pentapeptide	No result	No result
decaprenyl-pyrophosphoryl-(N-acetylglucosamine)-N-acetylmuramyl-(pentapeptide)	No result	No result
demethylated methyl donor	No result	No result
D-erythro-7,8-dihydroneopterin	HMDB0002275	C04874
D-glucopyranose	HMDB0000122	C00031
D-glucopyranose 6-phosphate	HMDB0001401	C00092
D-glucosamine 6-phosphate	HMDB0001254	C00352
D-glutamate	HMDB0003339	C00217
D-glyceraldehyde 3-phosphate	HMDB0001112	C00118
D-glycero-D-manno-heptose 7-phosphate	No result	C19882
D-glycero-α-D-manno-heptose 1,7-bisphosphate	No result	C19879
D-glycero-α-D-manno-heptose 1-phosphate	No result	C07838
diphosphate	HMDB0000250	C00013
di-trans,octa-cis-undecaprenyl diphosphate	No result	C04574
di-trans,octa-cis-undecaprenyl phosphate	No result	C17556
ditrans,octacis-undecaprenyldiphospho-[(N-acetyl-β-D-glucosaminyl)-(1,4)-]-N-acetyl-α-D-muramoyl-L-alanyl-γ-D-glutamyl-meso-2,6-diaminopimeloyl-D-alanyl-D-alanine	No result	C05898
ditrans,octacis-undecaprenyldiphospho-N-acetyl-(N-acetylglucosaminyl)muramoyl-L-alanyl-γ-D-isoglutaminyl-L-lysyl-D-alanyl-D-alanine	No result	No result
ditrans,octacis-undecaprenyldiphospho-N-acetyl-(N-acetylglucosaminyl)muramoyl-L-alanyl-γ-D-isoglutaminyl-N-(β-D-asparaginyl)-L-lysyl-D-alanyl-D-alanine	No result	No result
ditrans,octacis-undecaprenyldiphospho-N-acetyl-(N-acetylglucosaminyl)muramoyl-L-alanyl-γ-D-isoglutaminyl-N-(β-D-asparatyl)-L-lysyl-D-alanyl-D-alanine	No result	No result
ditrans,octacis-undecaprenyldiphospho-N-acetyl-(N-acetyl-β-D-glucosaminyl)muramoyl-L-alanyl-γ-D-glutamyl-L-lysyl-D-alanyl-D-alanine	No result	C05889
ditrans,octacis-undecaprenyldiphospho-N-acetyl-(N-acetyl-β-D-glucosaminyl)muramoyl-L-alanyl-γ-O-phospho-D-glutamyl-L-lysyl-D-alanyl-D-alanine	No result	No result
D-mannopyranose 6-phosphate	HMDB0001078	C00275
D-ribulose 5-phosphate	HMDB0000618	C00199
D-sedoheptulose 7-phosphate	HMDB0001068	C05382
dTDP-4-dehydro-6-deoxy-α-D-glucopyranose	HMDB0001399	C11907
dTDP-4-dehydro-β-L-rhamnose	HMDB0001399	C00688
dTDP-α-D-glucose	HMDB0001328	C00842
dTDP-β-L-rhamnose	HMDB0006354	C03319
D-threo-isocitrate	HMDB0001874	C00451
dTMP	HMDB0001227	C00364
dTTP	HMDB0001342	C00459
dUMP	HMDB0001409	C00365
electron-transfer quinol	No result	No result
electron-transfer quinone	No result	No result
epoxyqueuosine34 in tRNA	No result	No result
ethanol	HMDB0000108	C00469
FAD	HMDB0001248	C00016
FMN	HMDB0001520	C00061
formate	HMDB0000142	C00058
fumarate	HMDB0000134	C00122
GDP-D-glycero-α-D-manno-heptose	No result	C19881
GDP-α-D-mannose	HMDB0001163	C00096
geranyl diphosphate	HMDB0001285	C00341
geranylgeranyl diphosphate	HMDB0004486	C00353
glucosamine 1,6-diphosphate	No result	No result
glycerone phosphate	HMDB0001473	C00111
glycine	HMDB0000123	C00037
a glycogen	HMDB0000757	C00182

glycolaldehyde	HMDB0003344	C00266
glyoxylate	HMDB0000119	C00048
gondoyl-[acp]	No result	No result
GTP	HMDB0001273	C00044
guanine	HMDB0000132	C00242
guanine34 in tRNA	No result	No result
H+	HMDB0059597	C00080
H2	HMDB0001362	C00282
H2O	HMDB0002111	C00001
hydrogen peroxide	HMDB0003125	C00027
hydrogencarbonate	HMDB0000595	C00288
hypoxanthine	HMDB0000157	C00262
isopentenyl diphosphate	HMDB0001347	C00129
L-alanine	HMDB0000161	C00041
L-aspartate	HMDB0000191	C00049
L-cysteine	HMDB0000574	C00097
L-glutamate	HMDB0000148	C00025
L-glutamine	HMDB0000641	C00064
L-homocysteine	HMDB0000742	C00155
lipid A disaccharide (E. coli)	No result	C04932
lipid IVA (E. coli)	No result	C04919
lipid X (E. coli)	No result	C04824
L-lysine	HMDB0000182	C00047
L-methionine	HMDB0000696	C00073
malonyl-[acp]	No result	No result
mature peptidoglycan with (L-alanyl-γ-D-glutamyl-meso-2,6-diaminopimelate) tripeptide	No result	No result
mature peptidoglycan with (L-alanyl-γ-D-glutamyl-meso-2,6-diaminopimeloyl-D-alanine) tetrapeptide	No result	No result
mature peptidoglycan with (L-alanyl-γ-D-glutamyl-meso-2,6-diaminopimeloyl-D-alanyl-D-alanine) pentapeptide	No result	No result
mature peptidoglycan with (L-alanyl-γ-D-glutamyl-meso-2,6-diaminopimeloyl-glycine) tetrapeptide	No result	No result
mature peptidoglycan with D,D cross-links (meso-diaminopimelate containing)	No result	No result
mature peptidoglycan with L,D cross-links (meso-diaminopimelate containing)	No result	No result
a menaquinol	HMDB0060487	C05819
a menaquinone	No result	C00828
meso-diaminopimelate	HMDB0001370	C00680
methylated methyl donor	No result	No result
mono-trans,octa-cis-decaprenyl diphosphate	No result	No result
N1-(5-phospho-β-D-ribosyl)glycinamide	HMDB0002022	C03838
an N10-formyltetrahydrofolate	HMDB0000972	C00234
N2-formyl-N1-(5-phospho-β-D-ribosyl)glycinamide	HMDB0001308	C04376
N-acetyl-D-glucosamine 6-phosphate	HMDB0002817	C00357
N-acetyl-D-mannosamine	HMDB0001129	C00645
N-acetyl-D-mannosamine 6-phosphate	HMDB0001121	C04257
N-acetylneuraminate	HMDB0000230	C00270
N-acetyl-α-D-glucosamine 1-phosphate	HMDB0001367	C04256
NAD+	HMDB0000902	C00003
NADH	HMDB0001487	C00004
NADP+	HMDB0000217	C00006
NADPH	HMDB0000221	C00005
nascent peptidoglycan with (L-alanyl-γ-D-glutamyl-meso-2,6-diaminopimelate) tripeptide	No result	No result
nascent peptidoglycan with (L-alanyl-γ-D-glutamyl-meso-2,6-diaminopimeloyl-D-alanine) tetrapeptide	No result	No result
nascent peptidoglycan with (L-alanyl-γ-D-glutamyl-meso-2,6-diaminopimeloyl-D-alanyl-D-alanine) pentapeptide	No result	No result
nascent peptidoglycan with (L-alanyl-γ-D-glutamyl-meso-2,6-diaminopimeloyl-glycine) tetrapeptide	No result	No result
nascent peptidoglycan with D,D cross-links (meso-diaminopimelate containing)	No result	No result
nascent peptidoglycan with L,D cross links (meso-diaminopimelate containing)	No result	No result
nicotinate adenine dinucleotide	HMDB0001179	C00857
nucleoside diphosphate	No result	C00454
nucleoside triphosphate	HMDB0060500	C00201
oleoyl-[acp]	No result	No result
oxaloacetate	HMDB0000223	C00036
oxalosuccinate	HMDB0003974	C05379
oxidized ferredoxin [iron-sulfur] cluster	No result	No result
oxidized flavodoxin	No result	No result
oxidized hydrogenase 3	No result	No result
oxidized unknown electron carrier	No result	No result
oxygen	HMDB0001377	C00007
palmitoleoyl-[acp]	No result	No result
peptidoglycan dimer (E. faeciums)	No result	No result
peptidoglycan dimer (mycobacteria)	No result	No result
peptidoglycan tetramer with D,D cross-link (Enterococcus faecium)	No result	No result
peptidoglycan with (L-alanA99:A113yl-γ-D-glutamyl-L-lysyl-D-alanyl-D-alanine) pentapeptide	No result	No result

peptidoglycan with (L-alanyl-γ-D-glutamyl-meso-2,6-diaminopimeloyl-D-alanine) tetrapeptide	No result	No result
peptidoglycan with D,D cross-links (mycobacteria)	No result	No result
peptidoglycan with L,D cross-links (mycobacteria)	No result	No result
phosphate	HMDB0001429	C00009
phosphoenolpyruvate	HMDB0000263	C00074
phosphoglucomutase	No result	No result
phosphoglucosamine mutase	No result	No result
phosphorylated phosphoglucomutase	No result	No result
phosphorylated phosphoglucosamine mutase	No result	No result
prenyl diphosphate	HMDB0001120	C00235
preQ0	HMDB0002268	C15996
preQ1	HMDB0011690	C16675
propanoate	HMDB0000237	C00163
propanoyl-CoA	HMDB0001275	C00100
pyruvate	HMDB0000243	C00022
queuosine34 in tRNA	No result	No result
quinolinate	HMDB0000232	C03722
reduced ferredoxin [iron-sulfur] cluster	No result	No result
reduced flavodoxin	No result	No result
reduced hydrogenase 3	No result	No result
reduced unknown electron carrier	No result	No result
riboflavin	HMDB0000244	C00255
S-adenosyl-L-homocysteine	HMDB0000939	C00021
S-adenosyl-L-methionine	HMDB0001185	C00019
soluble [acyl-carrier protein]	No result	No result
S-ribosyl-L-homocysteine	No result	C03539
succinate	HMDB0000254	C00042
succinyl-CoA	HMDB0001022	C00091
sucrose	HMDB0000258	C00089
taxa-4,11-diene	No result	C11894
a tetrahydrofolate	HMDB0001846	C00101
tetrahydropteroyl tri-L-glutamate	HMDB0012290	C04144
thiamine diphosphate	HMDB0001372	C00068
thiamine phosphate	HMDB0002666	C01081
thymidine	HMDB0000273	C00214
thymine	HMDB0000262	C00178
trans,octacis-decaprenyl phosphate	No result	C02970
triphosphate	HMDB0003379	C02174
UDP	HMDB0000295	C00015
UDP-2,3-diacetamido-2,3-dideoxy-α-D-glucuronate	No result	No result
UDP-2,3-diacetamido-2,3-dideoxy-α-D-mannuronate	No result	No result
UDP-2,6-dideoxy-2-acetamidino-β-L-galactose	No result	No result
UDP-2-acetamido-2,6-dideoxy-α-D-xylo-hex-4-ulose	No result	C04613
UDP-2-acetamido-2,6-dideoxy-β-L-arabino-hex-4-ulose	No result	No result
UDP-2-acetamido-2,6-dideoxy-β-L-lyxo-4-hexulose	No result	No result
UDP-2-acetamido-2-deoxy-α-D-ribo-hex-3-uluronate	No result	No result
UDP-2-acetamido-3-acetamidino-2,3-dideoxy-α-D-mannuronate	No result	No result
UDP-2-acetamido-3-amino-2,3-dideoxy-α-D-glucuronate	No result	No result
UDP-2-N,3-O-bis[(3R)-3-hydroxytetradecanoyl]-α-D-glucosamine	No result	C04652
UDP-3-O-(3-hydroxymyristoyl)-α-D-glucosamine	No result	C06022
UDP-3-O-[(3R)-3-hydroxymyristoyl]-N-acetyl-α-D-glucosamine	No result	C04738
UDP-N-acetyl-α-D-fucosamine	No result	No result
UDP-N-acetyl-α-D-galactosaminouronate	No result	No result
UDP-N-acetyl-α-D-glucosamine	HMDB0000290	C00043
UDP-N-acetyl-α-D-glucosamine-enolpyruvate	No result	C04631
UDP-N-acetyl-α-D-glucosaminouronate	No result	C04573
UDP-N-acetyl-α-D-mannosamine	HMDB0013112	C01170
UDP-N-acetyl-α-D-mannosaminuronate	No result	C06240
UDP-N-acetyl-α-D-muramate	HMDB0011720	C01050
UDP-N-acetyl-α-D-muramoyl-L-alanine	No result	C01212
UDP-N-acetyl-α-D-muramoyl-L-alanyl-D-glutamate	No result	C00692
UDP-N-acetyl-α-D-muramoyl-L-alanyl-γ-D-glutamyl-L-lysine	HMDB0004207	C04700
UDP-N-acetyl-α-D-muramoyl-L-alanyl-γ-D-glutamyl-L-lysyl-D-alanyl-D-alanine	No result	C04846
UDP-N-acetyl-α-D-muramoyl-L-alanyl-γ-D-glutamyl-meso-2,6-diaminopimelate	No result	C04877
UDP-N-acetyl-α-D-muramoyl-L-alanyl-γ-D-glutamyl-meso-2,6-diaminopimeloyl-D-alanyl-D-alanine	No result	C04882
UDP-N-acetyl-α-D-quinovosamine	No result	No result
UDP-N-acetyl-β-L-fucosamine	No result	No result
UDP-N-acetyl-β-L-pneumosamine	No result	No result
UDP-N-acetyl-β-L-quinovosamine	No result	No result
UDP-N-acetyl-β-L-rhamnosamine	No result	No result
UDP-α-D-galactofuranose	No result	C03733
UDP-α-D-galactose	HMDB0000302	C00052
UDP-α-D-glucose	HMDB0000286	C00029
UMP	HMDB0000288	C00105
undecaprenyl-diphospho-N-acetylmuramoyl-L-alanyl-γ-D-glutamyl-L-lysyl- D-alanyl-D-alanine	No result	C05888
undecaprenyldiphospho-N-acetylmuramoyl-L-alanyl-γ-D-glutamyl-meso-2,6-diaminopimeloyl-D-alanyl-D-alanine	No result	C05897

uracil	HMDB0000300	C00106
UTP	HMDB0000285	C00075
α-D-galactopyranose	HMDB0000143	C00984
α-D-galactose 1-phosphate	HMDB0000645	C00446
α-D-glucopyranose 1-phosphate	HMDB0001586	C00103
α-D-glucosamine 1-phosphate	HMDB0001109	C06156
α-D-glucosamine 6-phosphate	No result	No result
α-D-mannose 1-phosphate	HMDB0006330	C00636
α-glucose 1,6-bisphosphate	HMDB0003514	C01231
α-Kdo-(2->4)-α-Kdo-(2->4)-α-Kdo-(2->6)-lipid IVA	No result	No result
α-Kdo-(2->4)-α-Kdo-(2->6)-lipid IVA (E. coli)	No result	C06025
α-Kdo-(2->8)-[α-Kdo-(2->4)]-α-Kdo-(2->4)-α-Kdo-(2->6)-lipid IVA	No result	No result
α-Kdo-(2->8)-α-Kdo-(2->4)-α-Kdo-(2->6)-lipid IVA	No result	G13060
α-Kdo-(2→6)-lipid IVA (E. coli)	No result	C06025
β-alanine	HMDB0000056	C00099
β-D-fructofuranose	HMDB0000660	C02336
β-D-fructofuranose 6-phosphate	HMDB0003971	C05345
β-D-galactopyranose	HMDB0003449	C00962
β-nicotinate D-ribonucleotide	HMDB0001132	C01185
(2S,4S)-4-hydroxy-2,3,4,5-tetrahydrodipicolinate	No result	C20258
(D-alanyl)adenylate	No result	No result
(Gro-P)n-Gro-P-ManNAc-GlcNAc-PP-undecaprenol	No result	No result
(R)-5-phosphomevalonate	HMDB0001343	C01107
(R)-mevalonate	HMDB0000227	C00418
(R)-mevalonate diphosphate	HMDB0001090	C01143
(S)-2,3,4,5-tetrahydrodipicolinate	HMDB0012289	C03972
(S)-3-hydroxy-3-methylglutaryl-CoA	HMDB0001375	C00356
(S)-dihydroorotate	HMDB0003349	C00337
(S)-lactate	HMDB0000190	C00186
[(2-Glc)-Gro-P]n-Gro-P-ManNAc-GlcNAc-PP-undecaprenol	No result	No result
[protein] S-acetyl-L-cysteine	No result	No result
[protein]-L-cysteine	No result	No result
[protein]-L-histidine	No result	No result
[protein]-Nπ-phospho-L-histidine	No result	No result
1-(3-sn-phosphatidyl)-sn-glycerol 3-phosphate	No result	No result
1,2-diacyl-sn-glycerol 3-phosphate	No result	C00416
1-acyl-sn-glycerol 3-phosphate	No result	C00681
2,3-diphospho-D-glycerate	HMDB0001294	C01159
2/3-phospho-D-glycerate	No result	No result
2-GDP-[(2S,3S,6R)-5-amino-6-methyl-3,6-dihydro-2H-pyran-3-ol]	No result	No result
2-GDP-[(2S,3S,6R)-5-imino-6-methyloxan-3-ol]	No result	No result
2-O-D-alanyl-1-O-phosphatidylglycerol	No result	No result
2-phospho-D-glycerate	HMDB0003391	C00631
3-phospho-D-glycerate	HMDB0060180	C00197
3-phospho-D-glyceroyl phosphate	HMDB0001270	C00236
a 3-sn-phosphatidyl-L-serine	HMDB0010165	C02737
5-amino-1-(5-phospho-D-ribosyl)imidazole-4-carboxamide	HMDB0001517	C04677
5-amino-1-(5-phospho-D-ribosyl)imidazole-4-carboxylate	HMDB0006273	C04751
5'-deoxyadenosine	HMDB0001983	C05198
5-formamido-1-(5-phospho-D-ribosyl)-imidazole-4-carboxamide	HMDB0001439	C04734
5'-phosphoribosyl-4-(N-succinocarboxamide)-5-aminoimidazole	HMDB0000797	C04823
6-phospho D-glucono-1,5-lactone	HMDB0001127	C01236
acetoacetyl-CoA	HMDB0001484	C00332
acyl-[acyl-carrier protein]	No result	No result
acyl-CoA	No result	C00040
adenosine	HMDB0000050	C00212
adenylo-succinate	HMDB0000536	C03794
carbamate	HMDB0003551	C01563
carbamoyl phosphate	HMDB0001096	C00169
carboxyphosphate	No result	C20969
a cardiolipin	HMDB0056960	C05980
a CDP-diacylglycerol	HMDB0006968	C00269
CDP-glycerol	HMDB0059599	C00513
coproporphyrinogen III	HMDB0001261	C03263
D-alanyl-[D-alanyl carrier protein]	No result	No result
dCDP	HMDB0001245	C00705
dCTP	HMDB0000998	C00458
D-erythrose 4-phosphate	HMDB0001321	C00279
D-galactopyranose 6-phosphate	No result	C01113
D-gluconate 6-phosphate	HMDB0001316	C00345
D-ribose 5-phosphate	HMDB0001548	C00117
D-tagatofuranose 1,6-bisphosphate	HMDB0006872	C03785
D-tagatofuranose 6-phosphate	HMDB0006873	C01097
dTDP	HMDB0001274	C00363
D-xylulose 5-phosphate	HMDB0000868	C00231
ethanolamine	HMDB0000149	C00189
Fe2+	HMDB0000692	C00023
GDP	HMDB0001201	C00035
GDP-4-dehydro-3,6-dideoxy-α-D-mannose	No result	C20612
GDP-4-dehydro-6-deoxy-β-L-galactose	HMDB0001391	C14830

GDP-4-dehydro-α-D-rhamnose	HMDB0001346	C01222
GDP-6-deoxy-α-D-talose	No result	C02977
GDP-α-D-perosamine	No result	No result
GDP-α-D-rhamnose	HMDB0001499	C03117
GDP-β-colitose	No result	C20613
GDP-β-L-fucose	No result	C00325
glycerol	HMDB0000131	C00116
GMP	HMDB0001397	C00144
Gro-P-ManNAc-GlcNAc-PP-undecaprenol	No result	No result
guanosine	HMDB0000133	C00387
holo [D-alanyl carrier protein]	No result	No result
IMP	HMDB0000175	C00130
inosine	HMDB0000195	C00294
L,L-diaminopimelate	HMDB0001370	C00666
an L-1-phosphatidylethanolamine	HMDB0008974	C00350
L-1-phosphatidyl-sn-glycerol	No result	C00344
L-2-acetamido-6-oxoheptanedioate	No result	C05539
lactose 6'-phosphate	HMDB0006789	C05396
L-aspartate 4-semialdehyde	HMDB0012249	C00441
L-aspartyl-4-phosphate	HMDB0012250	C03082
L-serine	HMDB0000187	C00065
L-α-amino-ε-keto-pimelate	No result	C03871
ManNAc-GlcNAc-PP-undecaprenol	No result	C04881
N5-carboxyaminoimidazole ribonucleotide	HMDB0012268	C15667
N-acetyl-L,L-2,6-diaminopimelate	No result	C04390
N-acetyl-α-D-glucosaminyldiphospho-ditrans,octacis-undecaprenol	No result	C01289
N-carbamoyl-L-aspartate	HMDB0000828	C00438
orotate	HMDB0000226	C00295
orotidine 5'-phosphate	HMDB0000218	C01103
peptidoglycan-major wall teichoic acid complex (B. subtilis 168)	No result	No result
polyisoprenyl-major wall teichoic acid (B. subtilis 168)	No result	C04457
protoheme	HMDB0003178	C00032
protoporphyrin IX	HMDB0000241	C02191
protoporphyrinogen IX	HMDB0001097	C01079
sn-glycerol 3-phosphate	HMDB0000126	C00093
urate	HMDB0000289	C00366
uroporphyrinogen-III	HMDB0001086	C01051
xanthine	HMDB0000292	C00385
xanthosine	HMDB0000299	C01762
XMP	HMDB0001554	C00655
α-D-ribose-1-phosphate	HMDB0001489	C00620
β-D-fructose 1,6-bisphosphate	HMDB0001058	C00354
(1R,6R)-6-hydroxy-2-succinylcyclohexa-2,4-diene-1-carboxylate	No result	C05817
(2Z)-2-hydroxyhexa-2,5-dienoate	No result	No result
(2Z)-2-hydroxypenta-2,4-dienoate	No result	No result
(2Z,4E)-2-hydroxy-5-methyl-6-oxohexa-2,4-dienoate	No result	C06760
(2Z,4E)-2-hydroxy-6-oxohepta-2,4-dienoate	No result	No result
(6S)-5-formyltetrahydrofolate	No result	No result
(S)-(+)-allantoin	No result	C02350
(S)-4-hydroxy-2-oxohexanoate	No result	No result
(S)-4-hydroxy-2-oxopentanoate	No result	C03589
(S)-lactaldehyde	HMDB0003052	C00424
(S)-ureidoglycine	No result	C02091
(S)-ureidoglycolate	HMDB0001005	C00603
[DsrE3A thiosulfate-carrier protein]-L-cysteine	No result	No result
[DsrE3A thiosulfate-carrier protein]-L-cysteine-S-thiosulfonate	No result	No result
[glycine-cleavage complex H protein] N6-aminomethyldihydrolipoyl-L-lysine	No result	No result
[glycine-cleavage complex H protein] N6-dihydrolipoyl-L-lysine	No result	No result
[glycine-cleavage complex H protein] N6-lipoyl-L-lysine	No result	No result
[L-cysteine desulfurase]-L-cysteine	No result	No result
[L-cysteine desulfurase]-S-sulfanyl-L-cysteine	No result	No result
[ThiI sulfur-carrier protein]-L-cysteine	No result	No result
[ThiI sulfur-carrier protein]-S-sulfanyl-L-cysteine	No result	No result
[TusA]-L-cysteine	No result	No result
[TusA]-L-cysteine-S-thiosulfonate	No result	No result
1,4-dihydroxy-2-naphthoate	No result	C03657
1,4-dihydroxy-2-naphthoyl-CoA	No result	C15547
1D-chiro-inositol	HMDB0240209	C19891
1-keto-D-chiro-inositol	No result	C20251
1-propanal	HMDB0003366	C00479
2-[(2R,5Z)-2-carboxy-4-methylthiazol-5(2H)-ylidene]ethyl phosphate	No result	C20246
2-iminoacetate	No result	C15809
2-methylphenol	HMDB0002055	C01542
2-succinyl-5-enolpyruvoyl-6-hydroxy-3-cyclohexene-1-carboxylate	No result	C16519
2-succinylbenzoate	No result	C02730
3D-(3,5/4)-trihydroxycyclohexane-1,2-dione	No result	C04287
3-methylcatechol	No result	C02923
3-oxopropanoate	HMDB0011111	C00222
3'-phosphoadenylyl-sulfate	HMDB0001134	C00053
3-phosphooxypyruvate	HMDB0001024	C03232

4-(2'-carboxyphenyl)-4-oxobutyryl-CoA	No result	C03160
4-imidazolone-5-propanoate	HMDB0001014	C03680
4-methylcatechol	HMDB0000873	C06730
4-methylphenol	HMDB0001858	C01468
5-dehydro-2-deoxy-D-gluconate	No result	C06892
5-dehydro-2-deoxy-D-gluconate 6-phosphate	No result	C06893
5-deoxy-D-glucuronate	No result	C16737
a 5-methyltetrahydrofolate	HMDB0001396	D09353
adenosine 3',5'-bisphosphate	HMDB0000061	C00054
adenosine 5'-phosphosulfate	HMDB0001003	C00224
agmatine	HMDB0001432	C00179
allantoate	HMDB0001209	C00499
all-trans-heptaprenyl diphosphate	HMDB0012187	C04216
all-trans-octaprenyl diphosphate	HMDB0001094	C04146
aminopropylcadaverine	HMDB0012189	C16565
cadaverine	HMDB0002322	C01672
carbon monoxide	HMDB0001426	C00067
carboxy-adenylated-[ThiS sulfur-carrier protein]	No result	No result
dADP	HMDB0001508	C00206
dATP	HMDB0001532	C00131
demethylmenaquinol-7	No result	No result
demethylmenaquinol-8	No result	No result
ditrans,octacis-undecaprenyldiphospho-N-acetyl-(N-acetylglucosaminyl)muramoyl-L-alanyl-γ-D-glutamyl-L-lysyl-D-alanine	No result	No result
ditrans,octacis-undecaprenyldiphospho-N-acetyl-(N-acetylglucosaminyl)muramoyl-L-alanyl-γ-D-isoglutaminyl-L-lysyl-(glycyl)3-D-alanyl-D-alanine	No result	No result
ditrans,octacis-undecaprenyldiphospho-N-acetyl-(N-acetylglucosaminyl)muramoyl-L-alanyl-γ-D-isoglutaminyl-L-lysyl-(glycyl)5-D-alanyl-D-alanine	No result	No result
ditrans,octacis-undecaprenyldiphospho-N-acetyl-(N-acetylglucosaminyl)muramoyl-L-alanyl-γ-D-isoglutaminyl-L-lysyl-(N6-glycyl)-D-alanyl-D-alanine	No result	No result
ditrans,octacis-undecaprenyldiphospho-N-acetyl-(N-acetylglucosaminyl)muramoyl-L-alanyl-γ-D-isoglutaminyl-L-lysyl-D-alanine	No result	No result
ditrans,octacis-undecaprenyldiphospho-N-acetyl-(N-acetylglucosaminyl)muramoyl-L-alanyl-γ-D-isoglutaminyl-N-(β-D-asparaginyl)-L-lysyl-D-alanine	No result	No result
ditrans,octacis-undecaprenyldiphospho-N-acetyl-(N-acetylglucosaminyl)muramoyl-L-alanyl-γ-D-isoglutaminyl-N-(β-D-asparatyl)-L-lysyl-D-alanine	No result	No result
elongator tRNAMet	No result	No result
formaldehyde	HMDB0001426	C00067
formamide	HMDB0001536	C00488
glycyl-[tRNAGly]	No result	No result
hexulose 6-phosphate	No result	C06019
hydrogen sulfide	HMDB0003276	C00087
initiator tRNAMet	No result	No result
isochorismate	No result	C00885
keto-L-rhamnulose	No result	No result
L-alanyl-[tRNAAla]	No result	No result
L-arginine	HMDB0000517	C00062
L-arginino-succinate	HMDB0000052	C03406
L-arginyl-[tRNAArg]	No result	No result
L-asparagine	HMDB0000168	C00152
L-asparaginyl-[tRNAAsn]	No result	No result
L-aspartyl-[tRNAasp]	No result	No result
L-citrulline	HMDB0000904	C00327
L-cysteinyl-[tRNACys]	No result	No result
L-glutaminyl-[tRNAGln]	No result	No result
L-glutamyl-[tRNAGlu]	No result	No result
L-histidine	HMDB0000177	C00135
L-histidyl-[tRNAHis]	No result	No result
L-homoserine	HMDB0000719	C00263
L-isoleucine	HMDB0000172	C00407
L-isoleucyl-[tRNAIle]	No result	No result
L-leucine	HMDB0000687	C00123
L-leucyl-[tRNALeu]	No result	No result
L-lysyl-[tRNALys]	No result	No result
L-methionyl-[elongator tRNAMet]	No result	No result
L-methionyl-[initiator tRNAMet]	No result	No result
L-ornithine	HMDB0000214	C00077
L-phenylalanine	HMDB0000159	C00079
L-phenylalanyl-[tRNAPhe]	No result	No result
L-proline	HMDB0000162	C00148
L-prolyl-[tRNAPro]	No result	No result
L-rhamnulofuranose	No result	No result
L-rhamnulose 1-phosphate	No result	C01131
L-seryl-[tRNAser]	No result	No result
L-threonine	HMDB0000167	C00188
L-threonyl-[tRNAThr]	No result	No result
L-tryptophan	HMDB0000929	C00078
L-tryptophanyl-[tRNATrp]	No result	No result
L-tyrosine	HMDB0000158	C00082
L-tyrosyl-[tRNATyr]	No result	No result

L-valine	HMDB0000883	C00183
L-valyl-[tRNAVal]	No result	No result
a maltodextrin	HMDB0037138	C01935
maltose	HMDB0000163	C00208
menaquinol-7	No result	No result
menaquinol-8	No result	No result
myo-inositol	HMDB0000211	C00137
N-acetylglutamyl-phosphate	HMDB0006456	C04133
N-acetyl-L-citrulline	HMDB0000856	C15532
N-acetyl-L-glutamate	HMDB0001138	C00624
N-acetyl-L-glutamate 5-semialdehyde	HMDB0006488	C01250
N-acetyl-L-ornithine	HMDB0003357	C00437
N-carbamoyl-2-oxoglycine	No result	C00802
N-carbamoylputrescine	HMDB0033458	C00436
N-formimino-L-glutamate	HMDB0000854	C00439
N-succinyl-2-amino-6-ketopimelate	HMDB0012266	C04462
N-succinyl-L,L-2,6-diaminopimelate	HMDB0012267	C04421
O-acetyl-L-homoserine	HMDB0029423	C01077
O-acetyl-L-serine	HMDB0003011	C00979
O-phospho-L-serine	HMDB0000272	C01005
oxamate	No result	C01444
oxidized c-type cytochrome	No result	No result
oxidized thioredoxin	No result	No result
peptidoglycan dimer (E. faecium, tetrapeptide)	No result	No result
peptidoglycan dimer (S. aureus)	No result	No result
peptidoglycan tetramer with an L,D cross-link (Enterococcus faecium)	No result	No result
peptidoglycan with (L-alanyl-γ-D-glutamyl-L-lysyl-D-alanyl-D-alanine) pentapeptide	No result	No result
peptidoglycan with D,D cross-link (S. aureus)	No result	No result
putrescine	HMDB0001414	C00134
an electron-transfer quinol	HMDB0002434	C00530
an electron-transfer quinone	HMDB0003364	C00472
reduced c-type cytochrome	No result	No result
reduced thioredoxin	No result	No result
S	No result	C00087
S-adenosyl 3-(methylsulfanyl)propylamine	HMDB0000988	C01137
scyllo-inositol	HMDB0006088	C06153
scyllo-inosose	No result	C00691
S-methyl-5'-thioadenosine	HMDB0001173	C00170
spermidine	HMDB0001257	C00315
spermine	HMDB0001256	C00750
starch	No result	C00369
sulfate	HMDB0001448	C00059
sulfite	HMDB0000240	C00094
tetrathionate	No result	C02084
thiocarboxy-[ThiS-Protein]	No result	No result
thiosulfate	HMDB0000257	C00320
ThiS sulfur-carrier protein	No result	No result
toluene	HMDB0034168	C01455
tRNAAla	No result	No result
tRNAArg	No result	No result
tRNAAsn	No result	No result
tRNAasp	No result	No result
tRNACys	No result	No result
tRNAGln	No result	No result
tRNAglu	No result	No result
tRNAGly	No result	No result
tRNAHis	No result	No result
tRNAIle	No result	No result
tRNALeu	No result	No result
tRNALys	No result	No result
tRNAPhe	No result	No result
tRNAPro	No result	No result
tRNAser	No result	No result
tRNAThr	No result	No result
tRNATrp	No result	No result
tRNATyr	No result	No result
tRNAVal	No result	No result
UDP-N-acetyl-α-D-muramoyl-L-alanyl-γ-D-glutamyl-L-lysyl-D-alanine	No result	C06432
undecaprenyl-diphospho-N-acetylmuramoyl-L-alanyl-γ-D-glutamyl-L-lysyl- D-alanine	No result	No result
urea	HMDB0000294	C00086
urocanate	HMDB0000301	C00785
α-L-rhamnopyranose	No result	C02476
β-L-rhamnopyranose	No result	C02338

Table S7. List of metabolites common to 16S predicted metagenomic pathways and LC-MS fecal metabolome

ion_mode	mz	HMDB IDs	KEGG	p value
neg	96.9597	HMDB0001448	C00059	4.66E-07
neg	256.057	HMDB0001129	C00645	1.53E-05
neg	142.049	HMDB0029423	C01077	0.000114562
pos	326.037	HMDB0001090	C01143	0.000151743
neg	323.0977	HMDB0000163	C00208	0.000293886
neg	323.0977	HMDB0000258	C00089	0.000293886
pos	256.0709	HMDB0000856	C15532	0.000514952
pos	342.0718	HMDB0000288	C00105	0.000524517
neg	166.0172	HMDB0000574	C00097	0.000608867
pos	154.0959	HMDB0033458	C00436	0.000629902
pos	166.0865	HMDB0000159	C00079	0.000642958
neg	209.0821	HMDB0000517	C00062	0.000644354
neg	164.0708	HMDB0000159	C00079	0.000811758
pos	313.1091	HMDB0000052	C03406	0.000919042
neg	130.0864	HMDB0000172	C00407	0.000992283
neg	130.0864	HMDB0000687	C00123	0.000992283
pos	140.0683	HMDB0000883	C00183	0.00103456
pos	132.1023	HMDB0000172	C00407	0.001252468
pos	132.1023	HMDB0000687	C00123	0.001252468
pos	355.1555	HMDB0000988	C01137	0.001344416
pos	118.0866	HMDB0000883	C00183	0.001459814
neg	152.011	HMDB0012249	C00441	0.001932727
pos	205.0969	HMDB0000929	C00078	0.002863727
pos	162.0758	HMDB0029423	C01077	0.002967643
neg	142.0501	HMDB0029423	C01077	0.003177012
neg	166.0137	HMDB0000232	C03722	0.004566663
pos	117.055	HMDB0000019	C00141	0.004681959
pos	188.0682	HMDB0000159	C00079	0.004998639
pos	284.101	HMDB0000133	C00387	0.005519946
pos	149.5522	HMDB0001173	C00170	0.005630117
neg	146.0607	HMDB0000159	C00079	0.005862979
pos	136.0624	HMDB0000034	C00147	0.006450368
neg	194.0485	HMDB0000696	C00073	0.007307417
neg	429.1211	HMDB0000939	C00021	0.008737521
pos	286.1162	HMDB0000195	C00294	0.008820423
pos	197.1005	HMDB0000517	C00062	0.009115102
pos	569.3131	HMDB0001097	C01079	0.00989505
neg	115.0391	HMDB0000019	C00141	0.010924159
neg	129.055	HMDB0000227	C00418	0.011071725
neg	129.055	HMDB0240389	C00522	0.011071725
neg	129.0548	HMDB0000227	C00418	0.01438629
neg	129.0548	HMDB0240389	C00522	0.01438629
neg	816.5704	HMDB0008974	C00350	0.016669852
neg	312.0951	HMDB0000050	C00212	0.01702771
neg	312.0951	HMDB0000085	C00330	0.01702771
pos	127.0496	HMDB0000071	C05512	0.018878133
pos	127.0496	HMDB0000262	C00178	0.018878133
pos	191.1042	HMDB0001370	C00680	0.01933916
pos	191.1042	HMDB0001370	C00666	0.01933916
pos	191.1042	HMDB0006488	C01250	0.01933916
neg	308.0973	HMDB0000230	C00270	0.019860257
neg	358.0235	HMDB0000095	C00055	0.021895167
neg	203.082	HMDB0000929	C00078	0.024434588
neg	117.0187	HMDB0000254	C00042	0.026377474
neg	103.0397	HMDB0003366	C00479	0.027858305
neg	148.0434	HMDB0000696	C00073	0.027999506
neg	103.0394	HMDB0003366	C00479	0.030000225
pos	553.4365	HMDB0002268	C15996	0.031352368
neg	235.0088	HMDB0003974	C05379	0.031655029
neg	264.0046	HMDB0001128	C03090	0.032109886
neg	508.1367	HMDB0000972	C00234	0.032543219
pos	230.0423	HMDB0001128	C03090	0.03266976
neg	274.0323	HMDB0001128	C03090	0.033722878
pos	195.0042	HMDB0000126	C00093	0.0345694
neg	216.0453	HMDB0000158	C00082	0.037935864
pos	150.0585	HMDB0000696	C00073	0.038020424
neg	228.0264	HMDB0001128	C03090	0.041240057
pos	268.1032	HMDB0000050	C00212	0.046666535
pos	268.1032	HMDB0000085	C00330	0.046666535
pos	672.4214	HMDB0012187	C04216	0.051790602
neg	233.0676	HMDB0000071	C05512	0.054424311
neg	142.0501	HMDB0029423	C01077	0.06231438
pos	170.0663	HMDB0000292	C00385	0.0645986
neg	816.5695	HMDB0008974	C00350	0.066455856
pos	210.9777	HMDB0000126	C00093	0.068658923

neg	258.0376	HMDB0001109	C06156	0.069569656
neg	258.0376	HMDB0001254	C00352	0.069569656
neg	287.0857	HMDB0000273	C00214	0.072723475
pos	196.0582	HMDB0006488	C01250	0.093199688
neg	699.3806	HMDB0012187	C04216	0.097452336
pos	178.0589	HMDB0000177	C00135	0.104511492
pos	202.0107	HMDB0001024	C03232	0.111168874
neg	162.0548	HMDB0000158	C00082	0.114467462
pos	332.1437	HMDB0001412	C00921	0.117172076
pos	149.5518	HMDB0001173	C00170	0.12171073
pos	473.1851	HMDB0001354	C00445	0.124704738
pos	473.1851	HMDB0004486	C00353	0.124704738
neg	218.1027	HMDB0000210	C00864	0.127573934
neg	217.011	HMDB0000126	C00093	0.129694675
neg	162.0554	HMDB0000158	C00082	0.134494443
pos	220.0386	HMDB0000158	C00082	0.135732372
neg	89.024	HMDB0000190	C00186	0.139011625
neg	89.024	HMDB0000990	C00084	0.139011625
neg	89.024	HMDB0001311	C00256	0.139011625
pos	338.0659	HMDB0001132	C01185	0.148192507
pos	198.0834	HMDB0000904	C00327	0.150480411
pos	155.5599	HMDB0000230	C00270	0.152297187
pos	116.0708	HMDB0000162	C00148	0.152464844
neg	111.0192	HMDB0000300	C00106	0.153558952
pos	220.1185	HMDB0000210	C00864	0.166588729
pos	164.0702	HMDB0003826	C04332	0.172748335
neg	245.0086	HMDB0001321	C00279	0.179134228
pos	140.0688	HMDB0000883	C00183	0.179280532
neg	128.0346	HMDB0000148	C00025	0.182510171
neg	128.0346	HMDB0003011	C00979	0.182510171
neg	128.0346	HMDB0003339	C00217	0.182510171
pos	182.0821	HMDB0000158	C00082	0.187512363
neg	424.1375	HMDB0001056	C00415	0.188154533
neg	567.2938	HMDB0001097	C01079	0.188223631
neg	816.5695	HMDB0008974	C00350	0.193470039
neg	701.1854	HMDB0000757	C00182	0.195931076
neg	174.9554	HMDB0001494	C00227	0.207180494
neg	329.0298	HMDB0000175	C00130	0.214831592
neg	174.9553	HMDB0001494	C00227	0.218223267
pos	184.9857	HMDB0000208	C00026	0.226403213
pos	184.9857	HMDB0001024	C03232	0.226403213
pos	140.9958	HMDB0001494	C00227	0.233537561
neg	151.0257	HMDB0000292	C00385	0.234203532
pos	242.1021	HMDB0000210	C00864	0.240140829
neg	135.0302	HMDB0000157	C00262	0.284014602
neg	135.0302	HMDB0000190	C00186	0.284014602
neg	135.0302	HMDB0001311	C00256	0.284014602
neg	215.032	HMDB0000122	C00031	0.302796185
neg	215.032	HMDB0000143	C00984	0.302796185
neg	215.032	HMDB0000211	C00137	0.302796185
neg	215.032	HMDB0000660	C02336	0.302796185
neg	215.032	HMDB0003449	C00962	0.302796185
neg	215.032	HMDB0006088	C06153	0.302796185
neg	215.032	HMDB0240209	C19891	0.302796185
neg	172.0609	HMDB0006488	C01250	0.315198864
neg	328.0874	HMDB0000133	C00387	0.33183703
pos	349.1144	HMDB0003826	C04332	0.352646988
neg	934.4918	HMDB0006968	C00269	0.355751564
neg	202.9967	HMDB0000289	C00366	0.356253426
neg	180.0656	HMDB0000158	C00082	0.360168168
neg	132.0296	HMDB0000191	C00049	0.365105045
neg	132.0296	HMDB0006483	C00402	0.365105045
pos	75.0443	HMDB0000227	C00418	0.366665105
pos	75.0443	HMDB0000237	C00163	0.366665105
pos	75.0443	HMDB0003052	C00424	0.366665105
pos	75.0443	HMDB0240389	C00522	0.366665105
pos	174.075	HMDB0006488	C01250	0.36982751
pos	170.0661	HMDB0000292	C00385	0.382051287
pos	365.1047	HMDB0000163	C00208	0.420616911
pos	365.1047	HMDB0000258	C00089	0.420616911
neg	361.0947	HMDB0003826	C04332	0.432135144
neg	174.9553	HMDB0001494	C00227	0.449370898
neg	123.0444	HMDB0000873	C06730	0.465288529
neg	246.0401	HMDB0060410	C20239	0.469009543
pos	327.1403	HMDB0000230	C00270	0.477144044
pos	282.1371	HMDB0000241	C02191	0.487857618
neg	209.0816	HMDB0000517	C00062	0.489424348
neg	256.0673	HMDB0060410	C20239	0.517968015

pos	241.1768	HMDB0001256	C00750	0.542023102
pos	203.0522	HMDB0000122	C00031	0.544542899
pos	203.0522	HMDB0000143	C00984	0.544542899
pos	203.0522	HMDB0000211	C00137	0.544542899
pos	203.0522	HMDB0000660	C02336	0.544542899
pos	203.0522	HMDB0003449	C00962	0.544542899
pos	203.0522	HMDB0006088	C06153	0.544542899
pos	203.0522	HMDB0240209	C19891	0.544542899
neg	770.568	HMDB0008974	C00350	0.5453025
pos	829.5654	HMDB0010165	C02737	0.555168902
neg	217.0116	HMDB0000126	C00093	0.574510616
neg	73.0289	HMDB0000131	C00116	0.596376835
neg	73.0289	HMDB0000237	C00163	0.596376835
neg	73.0289	HMDB0003052	C00424	0.596376835
pos	169.086	HMDB0001432	C00179	0.601219202
pos	113.0354	HMDB0000300	C00106	0.604688474
pos	220.0392	HMDB0000158	C00082	0.61606529
pos	286.1139	HMDB0000195	C00294	0.637218851
pos	242.102	HMDB0000210	C00864	0.656785463
pos	446.1774	HMDB0001846	C00101	0.662193935
pos	689.2126	HMDB0000757	C00182	0.702032453
pos	137.0462	HMDB0000157	C00262	0.710901786
pos	329.0042	HMDB0001068	C05382	0.716372078
pos	128.0708	HMDB0002434	C00530	0.723334362
pos	580.2947	HMDB0000241	C02191	0.780772512
pos	93.0549	HMDB0000131	C00116	0.806729128
neg	377.0849	HMDB0000163	C00208	0.820630772
neg	377.0849	HMDB0000258	C00089	0.820630772
neg	480.1397	HMDB0001846	C00101	0.850308917
pos	677.3733	HMDB0012187	C04216	0.854807062
pos	586.3356	HMDB0001097	C01079	0.875145634
pos	90.0546	HMDB0000056	C00099	0.910262098
pos	90.0546	HMDB0000161	C00041	0.910262098
pos	90.0546	HMDB0001310	C00133	0.910262098
pos	156.0057	HMDB0012249	C00441	0.91777617
pos	120.0657	HMDB0000167	C00188	0.940490691
pos	120.0657	HMDB0000719	C00263	0.940490691
pos	117.0545	HMDB0000019	C00141	0.950929763
pos	394.1684	HMDB0000244	C00255	0.957078826
neg	699.3797	HMDB0012187	C04216	0.986709286
neg	162.0553	HMDB0000158	C00082	0.996372866
